# Dynamic Microbiome Responses to Structurally Diverse Anthocyanin-Rich Foods in a Western Diet Context

**DOI:** 10.3390/nu17132201

**Published:** 2025-07-01

**Authors:** Mohammed F. Almatani, Giovanni Rompato, Eliza C. Stewart, Marcus Hayden, Jeremy Case, Samuel Rice, Korry J. Hintze, Abby D. Benninghoff

**Affiliations:** 1 Department of Animal, Dairy and Veterinary Sciences, Utah State University, 4815 Old Main Hill, Logan, UT 84322, USA; mohammed.almatani@usu.edu (M.F.A.); giovanni.rompato@usu.edu (G.R.);; 2 Department of Pharmacology, College of Pharmacy, King Khalid University, Al Fara, Abha 62223, Saudi Arabia; 3 Department of Nutrition, Dietetics and Food Sciences, Utah State University, 8700 Old Main Hill, Logan, UT 84322, USA; korry.hintze@usu.edu

**Keywords:** fecal microbiome, dynamic response, diversity, anthocyanins, black raspberry, black currant, bilberry, elderberry, tart cherry, cocoa polyphenols

## Abstract

**Background/Objectives:** Anthocyanin (ACN)-rich foods are known to influence the gut microbiota composition, but the temporal dynamics and structural specificity of these effects remain poorly understood. This study investigated how distinct ACN-rich fruit supplements impact the gut microbiome over time in the context of a Western-style diet. We hypothesized that ACN-induced microbial shifts would occur rapidly, differ by ACN source, and require continued intake to persist. **Methods**: C57BL/6J mice were fed the total Western diet (TWD) supplemented with freeze-dried powders from bilberry (BB), tart cherry (TC), chokeberry (CB), elderberry (EB), black currant (BC), or black raspberry (BRB) for 0, 1, 3, or 7 days. Cocoa polyphenols (CPs) were included as a comparator with a distinct polyphenol profile. Fecal microbiota were collected at 0, 1, 3, and 7 days post exposure and analyzed by 16S rRNA sequencing. **Results**: ACN-rich supplements induced rapid microbial shifts detectable within one day of exposure. However, most changes reverted toward the baseline within days of supplement withdrawal, indicating limited persistence. Among the ACNs, BRB produced the most sustained microbiome alterations. Microbial responses varied by ACN source, suggesting that differences in glycoside and aglycone structures influence the community composition. **Conclusions**: ACN-rich foods can induce rapid but largely transient alterations in the gut microbiome, with variability linked to the polyphenol structure. These findings highlight the ecological sensitivity of the microbiome to specific dietary components and underscore the importance of sustained intake for maintaining microbial shifts.

## 1. Introduction

The gut microbiome, comprising bacteria, archaea, and fungi, constitutes a complex microbial ecosystem residing along the gastrointestinal epithelium. These microorganisms carry out essential functions that influence the host’s digestion, metabolism, and immune regulation [1,2]. Microbial density increases along the gastrointestinal tract, reaching its highest concentration and metabolic activity in the colon, where microbial cell counts are estimated at approximately 100 trillion [3]. Although the gut microbiota typically supports the host’s homeostasis, perturbations such as inflammation, antibiotic administration, or dietary shifts can lead to dysbiosis, characterized by reduced microbial diversity, the loss of beneficial taxa, and the expansion of pro-inflammatory pathobionts [4]. The microbial composition is shaped by multiple factors, with diet emerging as a major modulator of both the short- and long-term microbial community structure [5,6]. Among dietary constituents, plant-derived polyphenols have attracted interest for their bidirectional interactions with the gut microbiome. While poorly absorbed in the upper gastrointestinal tract, polyphenols reach the colon, where they are metabolized by gut microbes and can, in turn, modulate microbial abundance and diversity [7,8,9].

ACNs are glycosylated flavonoids found in deeply pigmented fruits and vegetables, such as berries, cherries, and currants. Studies have shown that ACN-rich diets may promote microbial diversity and selectively enrich for taxa associated with beneficial metabolic or immune profiles [10,11,12,13,14]. However, the temporal dynamics of these changes, including the speed of the microbial response to supplementation, the persistence of effects after cessation, and the influences of specific ACN structural profiles, remain poorly understood. Most preclinical studies to date have employed continuous feeding paradigms and single-source ACNs, limiting insight into the acute responsiveness and resilience of the microbiome under more variable intake patterns reflective of human behavior.

Another gap lies in the comparative effects of distinct ACN sources, which can differ significantly in their molecular structures, glycosylation patterns, and overall polyphenol compositions. These chemical differences are likely to influence microbial metabolism and colonization dynamics, but systematic comparisons across multiple ACN-rich foods are rare. Moreover, the context of the host’s diet, particularly Western dietary patterns rich in fat, sugar, and processed ingredients, may strongly affect microbial responses to polyphenols. To address this, we utilized a total Western diet (TWD) mouse model that recapitulates the macronutrient and micronutrient profiles of the typical American diet, providing a translationally relevant background for evaluating dietary interventions [15].

Anthocyanins (ACNs) exhibit substantial chemical diversity, with over 500 naturally occurring ACNs derived from approximately 23 anthocyanidins [16]. Among these, six core anthocyanidins predominate in edible plants, including cyanidin, pelargonidin, delphinidin, peonidin, petunidin, and malvidin; cyanidin accounts for nearly half of the anthocyanidin content in most fruits [16]. ACNs differ not only in their aglycone structures but also in glycosylation patterns, such as 3-monosides, 3-biosides, and 3,5-diglycosides, which significantly influence their solubility, stability, and microbial metabolism in the gut [16,17]. For example, cyanidin-3-glucoside is among the most abundant and stable forms, while other ACNs may be more prone to degradation, depending on pH, temperature, and molecular substitutions on the B-ring [18,19,20,21]. These structural nuances affect both ACN bioavailability and their interactions with specific gut microbes, leading to potentially distinct microbial signatures across different ACN-rich foods. Understanding how structurally diverse ACNs influence the microbial composition under physiologically relevant dietary conditions remains an important step toward designing targeted nutritional interventions.

In this study, we investigated how the gut microbiome responds to short-term dietary supplementation with six ACN-rich fruit powders, including bilberry, tart cherry, elderberry, chokeberry, black currant, and black raspberry, each selected to capture a range of anthocyanin structures and glycosylation patterns (Figure S1). We assessed the microbial composition via 16S rRNA sequencing across three exposure durations (1, 3, and 7 days) and four post-intervention recovery timepoints (0, 1, 3, and 7 days). We hypothesized that (1) ACN supplementation would induce rapid and measurable changes in the gut microbial composition; (2) these effects would vary in persistence after ACN withdrawal; and (3) distinct ACN sources would elicit different microbial signatures. By focusing on the ecological dynamics of the microbiome in response to structurally diverse dietary supplements, this work provides foundational insight into how complex polyphenol mixtures may modulate microbial communities under Westernized dietary conditions.

## 2. Materials and Methods

### 2.1. Analysis of Anthocyanin Supplements

**Figure 1:**
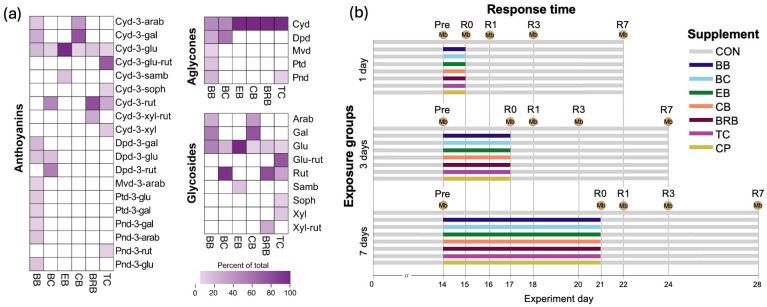
Experimental design for assessing the dynamic responses of mouse fecal microbiomes to diverse ACN diets. (**a**) Heatmaps illustrating the relative abundances of anthocyanins and their aglycone and glycoside components in ACN-rich food powders. The color scale represents the relative abundance (percentage of the total). (**b**) Mice were initially fed the total Western diet as the control basal diet and then fed supplements for 1, 3, or 7 days beginning on experiment day 14. Fecal microbiome samples (Mb) were collected at experiment day 0 (Pre), immediately following exposure (R0), or 1 day (R1), 3 days (R3), or 7 days (R7) following exposure. CON, control; BB, bilberry; BC, black currant; EB, elderberry; CB, chokeberry; BRB, black raspberry; TC, tart cherry; CPs, cocoa polyphenols; arab, arabinoside; cyd, cyanidin; dpd, delphinidin; gal, galactoside; glu, glucoside; glu-rut, glucosyl rutinoside; mvd, malvidin; pnd, pelargonidin; ptd, petunidin; rut, rutinoside; samb, sambubioside; soph, sophoroside; xyl-rut, xylosyl rutinoside; xyl, xyloside.

ACN-rich standardized berry extract powders for bilberry (BB), black currant (BC), elderberry (EB), chokeberry (aronia) (CB), and tart cherry (TC) were obtained from Artemis-International (Fort Wayne, IN, USA); black raspberry powder (BRB), a whole berry powder, was obtained from Berri Health (Corvallis, OR, USA) (Table S1). All the ACN-rich supplements were submitted to the Linus Pauling Institute Analytics Service Core (Oregon State University) for total ACN quantification by UV/vis spectrometry and for compositional analysis by high-performance liquid chromatography with photodiode array detection (HPLC PDA), as per methods described by Durst and Wrolstad [22] (Figure S2). The relative abundances of the ACNs and their aglycone and glycoside structural components in each powder supplement are provided in Figure 1a. We included cocoa polyphenols from CocoaVia Cardio Health^®^ powder (Mars Symbioscience, Germantown, MD, USA) as a part of the experimental design to compare ACN-rich supplements to a markedly different polyphenol profile. As per the manufacturer, CocoaVia powder contains 500 mg of total flavanols/6.4 g serving, of which 80 mg is (-)-epicatechin.

### 2.2. Animal and Experimental Diets

The Utah State University Institutional Animal Care and Use Committee (USU IACUC) approved all the procedures for the handling and treating of the mice used for this study (protocol 11605). Male C57BL/6J mice (*n* = 144) were obtained from Jackson Laboratories (Bar Harbor, ME, USA) at five weeks of age. The mice were housed in a specific pathogen-free vivarium in the Laboratory Animal Research Center (LARC) in the BioInnovations Center at Utah State University, an AAALAC-approved facility. The mice were singly housed in sterile micro isolator cages with Bed-o’Cobs^®^ ¼ bedding (Andersons, Cincinnati, OH, USA) supplied with HEPA-filtered micro isolator cages in an IVC Air-Handling Solutions ventilated housing system (Tecniplast, Buguggiate, Italy). Nesting material was provided as enrichment to reduce stress. The mice were maintained in a 12:12 h dark:light cycle, with 50% humidity, and in a specific pathogen-free vivarium with a temperature ranging from 18 to 23 °C. Following one week of quarantine, the mice were randomized and allocated to experimental groups, as outlined below. Fresh food was provided twice a week, and food consumption was monitored at each change (including accounting for spillage into the cage). The mice were provided with autoclaved drinking water ad libitum throughout the study. The cages were identified by supplement group and exposure time to facilitate the accurate administration of the experimental diets on the defined schedule.

The experimental diets were formulated by Envigo (Hackensack, NJ, USA) (Table S2), obtained from the vendor as one lot, and maintained at 4 °C for the duration of the study. For this study, we used the TWD (formulation previously published [15]) as the basal control diet, as this diet formulation emulates typical U.S. intakes of micro- and macronutrients on an energy density basis. All the supplement diets were designed to achieve a total ACN concentration of 0.2% (*w*/*w*) by varying the amount of the fruit powder added per kilogram of the diet, according to the measured total ACN. The resulting diets were as follows (all *w*/*w*): BB, 1.06% Std. Billberry Powder; BC, 2.2% Currant Craft; EB, 1.35% Elder Craft; CB, 0.29% Aronia Craft; BRB, 10.6% black raspberry powder; and TC, 0.89% Cherry Craft. The CP diet contained 2.56% (*w*/*w*) CocoaVia powder to provide 0.2% (*w*/*w*) total flavanols. The energy densities of the experimental diets were from 4.3 to 4.4 kcal/g (all within 0.1 kcal/g).

### 2.3. Experimental Design

The experimental design consisted of three primary factors: exposure group (1 day, 3 days, and 7 days), response time (R0, R1, R3, and R7), and supplement (CON, BB, BC, EB, CB, BRB, TC, and CPs) (Figure 1b). Following quarantine and acclimation, all the mice were weighed and then distributed by block randomization to each exposure group (*n* = 48), with *n* = 6 for each supplement within the exposure group. A power analysis was performed using the micropower R package (version 0.4), which first simulates beta-diversity distance matrices given population parameters computed from prior studies and then simulates a range of effect sizes and rarefaction curves to estimate the PERMANOVA power from the simulated distance matrices [23]. With *n* = 6 mice/group and a variance of 0.12, we have 80% power to detect an effect size (ω^2^) of 0.018 and 90% power to detect an effect size of 0.033. The power increases with more groups compared. This effect size is similar to that for other studies, as reported by Kelly et al. [23].

All the mice were provided the control basal diet (TWD) ad libitum for 14 days to acclimate the mice and their microbiomes to this Western basal diet. On day 14, the mice were then provided the respective supplement diets for different exposure durations consisting of 1, 3, or 7 days in total. Because we had previously observed that the mice provided the BRB-supplemented diet consumed more food than their control counterparts [24], beginning on day 14, the mice were provided from 2.5 to 3 g/day on average. The cages were monitored daily to ensure that the mice always had access to food. This feeding strategy did not impair the bodyweight gain (0.013 g/day over 4 weeks), which was at a similar rate to that in our prior work (0.011 g/day) in similarly aged male C57BL/6J mice fed the TWD basal diet [24]. At the end of each exposure period, all the mouse diets were switched back to the CON diet for 7 additional days.

Fresh fecal pellets were collected on experiment day 14, defined as the “Pre” group, representing the baseline microbiome before the supplement intervention. Then, fresh fecal pellets were obtained at response times 0 (immediately after exposure) and 1, 3, and 7 days following exposure (R0, R1, R3, and R7, respectively). The food intake was monitored with every fresh food change, and the bodyweight was monitored weekly. The body composition was determined by MRI scans (EchoMRI-700; EchoMRI, Houston, TX, USA) at day 14 (Pre), after each exposure time, and just prior to necropsy (Figure 1b). At the conclusion of the study, all the mice were anesthetized by CO_2_ asphyxiation and necropsied, and their ceca were weighed.

### 2.4. Microbiota Profiling by 16S rRNA Sequencing

The complete methods for the sample preparation, sequencing, and data processing have been previously described by Rodriguez et al. [25]. The samples were blinded to experimental groups prior to processing, with only the project director (A.D.B.) and a student researcher (M.F.A.) having access to the sample identification code and experimental group key. The samples remained blinded throughout preparation, sequencing, and data processing.

Briefly, DNA was extracted from mouse fecal pellets using the QIAamp DNA Stool Mini Kit (Qiagen, Frederick, MD, USA) following the manufacturer’s protocol, with the addition of mechanical disruption using zirconia/silica beads. The DNA quality and concentration were assessed by UV spectrophotometry, and the samples were diluted to 20 ng/μL in tris–EDTA (TE) buffer (pH 8.0). The V4 region of the 16S rRNA gene was amplified using primers 515F and 806R [25] and a two-step PCR protocol with platinum HS reagents (Thermo Fisher Scientific, Waltham, MA, USA). The amplicon size (~254 bp) was verified by gel electrophoresis. PCR products were purified using AMPure XP beads (Beckman Coulter, Indianapolis, IN, USA), quantified using the Quant-IT Picogreen dsDNA Assay (Thermo Fisher Scientific), and normalized to 1 ng/μL. The samples were pooled and stored at –20 °C prior to sequencing. Paired-end sequencing (2 × 250 bp) was performed using the Illumina MiSeq platform with the MiSeq Reagent Kit v2 (Illumina, San Diego, CA, USA).

Microbiota sequences were processed using QIIME 2 [26], which implements the full amplicon workflow (filtering, dereplication, chimera identification, and the merging of paired end reads) and generates an amplicon sequence variant (ASV) table and representative sequences. To assign the taxonomy, the Qiime feature classifier’s classify-sklearn command was used with a classifier pretrained for the V4 region, silva-138-99-515-806-nbclassifier.qza, and the Silva database (138 SSU) [27]. The resulting sequence count data aligned to the taxonomy are provided in File S1.

### 2.5. Microbiome Sequencing Data Analysis

Because the mice were singly housed, the individual mouse was considered as the biological unit. The taxonomy and alpha- and beta-diversity analyses were performed using the Microbiome Analyst Marker Data-Profiling module [28] with a minimum count of four, a low count filter of 20% prevalence, and low variance filter of 10% based on the inter-quantile range. The sequencing libraries were rarefied to the minimum library size with total sum scaling.

Given the complexity of the experimental design with three key factors of interest (exposure group, response time, and supplement), we determined a priori specific analytical questions that addressed our overarching objective and hypothesis (Figure S3). After first determining the main effects for each experimental factor, analyses proceeded in a stepwise fashion as follows: Q1—For each supplement, is the microbiome response dependent on the exposure group? In other words, does a longer exposure to the supplement lead to a more robust change in the microbiome? Q2—For each supplement, is the microbiome response dependent on the response time? In other words, does the change in the microbiome composition persist? Q3—For each exposure group, is the microbiome response dependent on the supplement? Q4—For each response time, is the microbiome response dependent on the supplement? For Q3 and Q4, in other words, how does the ACN profile of the supplement affect the microbiome response? And, lastly, Q5—For an exposure of 1 day with a response time of 0 days (i.e., immediate measurement), is the microbiome rapidly responsive to the supplement? In other words, is an effect of the ACN-rich food powder observed right away?

Measures of the α-diversity included the Chao1 richness (number of species represented) and Shannon index (the weighted abundance of the species present. Alpha-diversity scores were analyzed for the entire dataset to identify potential outlier microbiome profiles by applying the robust outlier test (ROUT) with a conservative *Q* value of 0.1% (GraphPad Prism v. 10.4.1, San Diego, CA, USA), meaning that there is a ≤ 0.1% chance of excluding a data point as an outlier in error; this approach was determined a priori. Based on the results of the ROUT test, 17 samples, or only 2.3% of the entire sample set, were identified as likely outliers with very low alpha-diversity scores and excluded from further analyses (Figure S4). Additionally, one sample failed to amplify during the processing.

The beta-diversity was determined using unweighted (a qualitative measure, which is sensitive to low-abundance features) and weighted (accounts for the abundance of species) UniFrac distances and was represented as principal coordinate plots (PCoAs) of the first two coordinates. A PERMANOVA *R*^2^ value of >0.1 with *p* < 0.01 was considered as statistically significant and biologically relevant for β-diversity analyses. Euclidean centroid distances were calculated for the response time within each supplement group to visualize the shift in the microbiomes with increasing time post exposure. Taxonomic relative abundance data were first analyzed using MetagenomeSeq fitZIG to determine the main effects of the experimental factors of the exposure group, response time, and supplement. Subsequent stepwise analyses were preformed using MaAsLan2 multivariate analyses (LM model), controlling for covariates as appropriate for each analytical question (e.g., for Q1, by considering the effect of the exposure group for each supplement, the model controlled for the response time). A false discovery rate (FDR)-adjusted *p*-value of < 0.05 for the MaAsLan2 post hoc tests was considered as statistically significant. The complete results of these statistical analyses are available in File S2. To visualize the effect of the supplement on the microbiome composition, for response time = R0, heat trees were generated to demonstrate taxonomic differences within pairwise comparisons of interest. The heat tree analysis leverages the hierarchical structure of taxonomic classifications to quantitatively (using the median abundance) and statistically (using the nonparametric Wilcoxon rank sum test) depict taxonomic differences between microbial communities [29].

### 2.6. Other Data Analyses

The data for the energy intake, bodyweight, body composition (percentage of the fat mass), cecum content, alpha-diversity, and the F:B ratio were analyzed using a generalized linear model with the restricted maximum likelihood estimation and the Tukey HSD post hoc test for multiple comparisons. Suspected outliers were verified using the robust outlier test (ROUT) with *Q* = 0.1% (Prism). For these analyses, an adjusted *p*-value of *<* 0.05 was considered as a significant effect of the test variable.

## 3. Results

### 3.1. Energy Intake, Bodyweight, Body Composition, and Cecum Weight

The energy intake decreased initially across all the groups (*p* < 0.001) yet stabilized after experiment day 7 (Figure S5), with no apparent effect of the supplement (*p* > 0.05). The bodyweight increased with increasing experimental duration, with a slight but not significant decrease in the 3-day exposure group. Although the bodyweight gain in the mice fed EB appeared to increase from days 14 to 21, in mice belonging to the 7-day exposure group, there were no significant main effects of the supplement (Figure S5a) and no significant differences among the supplement groups for the final bodyweight or the percentage of the fat mass, as determined by MRI (Figure S5b). Lastly, the cecum content as a percentage of the bodyweight did not significantly differ among the supplement groups.

### 3.2. Taxonomic Relative Abundance

Sequencing resulted in a total of 3.0 × 10^7^ reads of the 16S rRNA V4 region amplicon for all the samples combined. Following quality filtering and chimera removal, 2.1 × 10^7^ sequences were assigned to ASVs (Silva database version 138 SSU) using QIIME2, resulting in an average of 29,710 sequences per sample assigned to 2358 ASVs. After filtering for low prevalence and variance, as described in our methods, the sequence library was rarefied to a sequencing depth of ~5500 sequences (Figure S6). The fecal microbiome compositions and relative abundances of the identified bacteria are shown in Figures S7 and S8).

We first analyzed bacterial family-level abundances to determine how the exposure duration, response time, and supplement type shaped the gut microbiome composition (Figure S9). Marked shifts in the microbial composition were observed primarily for the exposure group (e.g., Akkermansiaceae, Atopobiaceae, Butyricicoccaceae, Eggerthellaceae, Monoglobaceae, Oscillospiraceae, Peptostreptococcaceae, Ruminococcaceae, and Sutterellaceae) and response time (e.g., Bacteroidaceae, Christensenellaceae, the Clostridia_vadinBB60_group, Monoglobaceae, Rikenellaceae, and Sutterellaceae). In contrast, fewer taxa exhibited notable differences due to the supplement alone, with only Monoglobaceae, Ruminococcaceae, and UCG_010 showing meaningful variations. These findings suggest that the exposure group and response time exert a greater influence on the microbiome composition than the supplement alone, emphasizing the importance of considering these variables in the experimental design.

**Figure 2:**
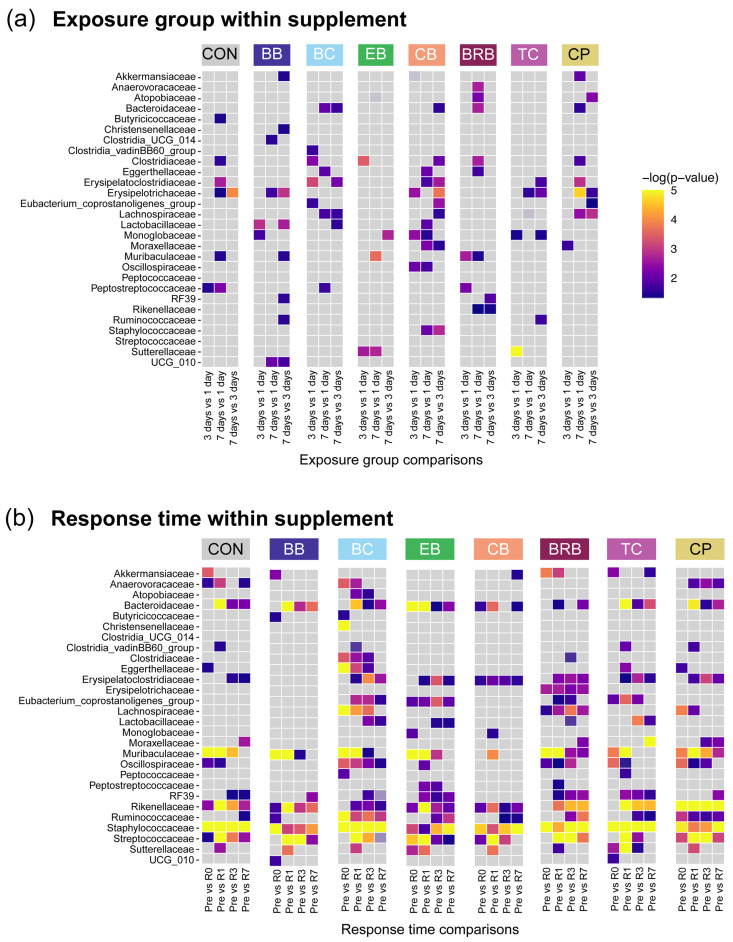
Statistical summary for the multivariate analysis of the bacterial family relative abundances for exposure groups and response times within each supplement. Heatmaps show the −log *p*-values for pairwise comparisons for (**a**) exposure groups, controlling for response times (*n* = 27 to 30) and (**b**) response times, controlling for exposure groups within each supplement (*n* = 16 to 18), as determined by MaAsLan2 multivariate analysis. Complete statistical results are available in File S2. CON, control; BB, bilberry; BC, black currant; EB, elderberry; CB, chokeberry; BRB, black raspberry; TC, tart cherry; CPs, cocoa polyphenols.

To determine whether longer exposures led to more robust microbiome changes (Q1), we compared bacterial family abundances across the 1-, 3-, and 7-day exposure groups, controlling for the response time (Figure 2a). No consistent trends were observed across the supplements, and no increasing pattern of significance was evident in the 3-day vs. 1-day or 7-day vs. 1-day comparisons. For example, the Erysipelotrichaceae abundance in the CON group differed significantly at 7 days, despite no dietary change, indicating a natural microbiome drift. Overall, these findings suggest that extended exposure alone did not intensify the microbiome changes.

In contrast, the response time (Q2) had a stronger influence (Figure 2b). Akkermansiaceae, for example, increased significantly at R0 in the BRB-fed (19.3% of the reads; *p* = 0.0166) and CP-fed (22.3%; *p* = 8.63 × 10^−4^) mice compared to CON (12.3%) and remained elevated at R1 in both groups (BRB: 15.5%, *p* = 0.0043; CPs: 20.8%, *p* = 8.94 × 10^−5^). However, this effect declined by R3 and R7. Similarly, the Bacteroidaceae abundance rose significantly at R0 in the BRB-fed (21.3%; *p* = 1.21 × 10^−5^), CP-fed (19.3%; *p* = 8.08 × 10^−4^), and BB-fed (15.9%; *p* = 0.0355) mice compared to CON (10.2%), but these changes were no longer detectable at later response times. Conversely, the Clostridiaceae abundance decreased sharply at R0 in the BRB-fed mice (3.7% vs. 16.8% in CON; *p* = 2.72 × 10^−4^). By R1, the differences narrowed but remained detectable between the BRB and other ACN groups (e.g., EB: *p* = 0.0015), again disappearing at R3 and R7. Erysipelatoclostridiaceae also decreased significantly at R0 in several groups, including BB (1.18%; *p* = 0.0198), EB (0.70%; *p* = 0.0038), BRB (0.90%; *p* = 0.0038), and CPs (1.56%; *p* = 0.0038), compared to CON (2.34%).

**Figure 3:**
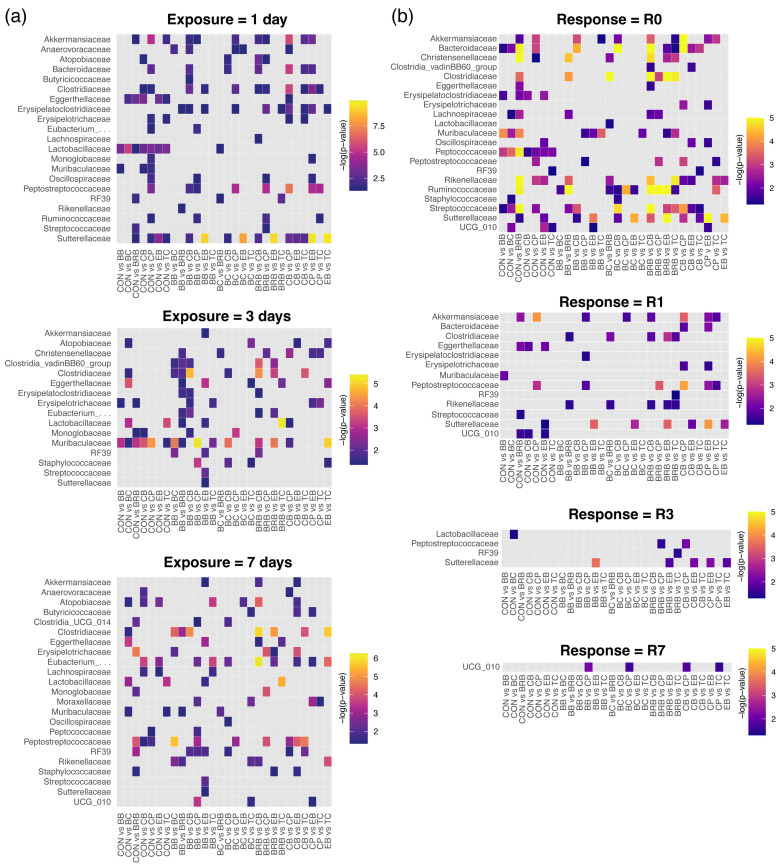
Statistical summary for the multivariate analysis of the bacterial family relative abundances for exposure groups and response times within each supplement. Heatmaps show the −log *p*-values for pairwise comparisons for (**a**) exposure groups (controlling for response times within each supplement) (*n* = 27 to 30) and (**b**) response times (controlling for exposure groups within each supplement) (*n* = 16 to 18), as determined by MaAsLan2 multivariate analysis. “Eubacterium_…” refers to Eubacterium_coprostanoligenes_group. Complete statistical results are available in File S2.

**Figure 4:**
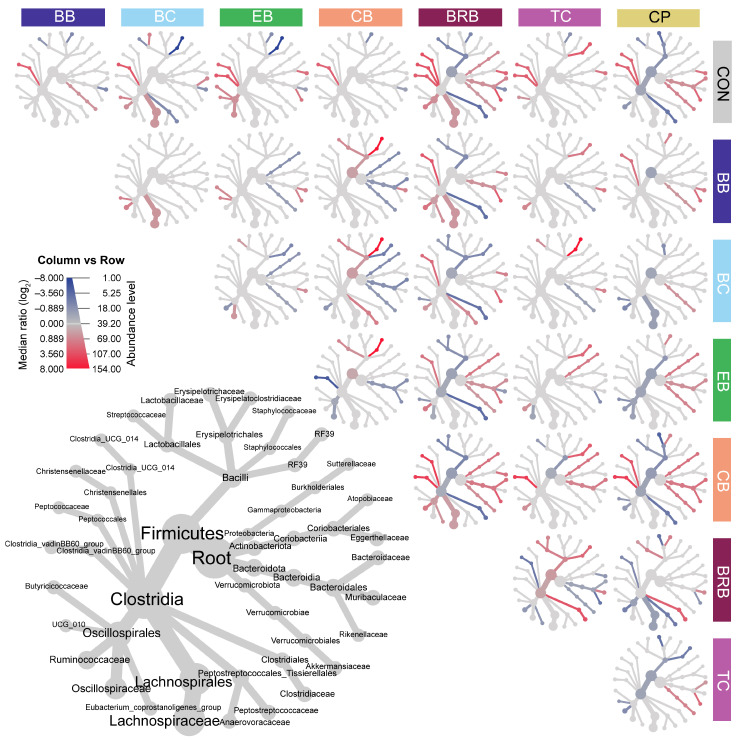
Fecal microbiome family-level community structures shown as heat trees, displaying the relative abundance ratios for pairwise comparisons within supplements for response time = R0 (*n* = 16 to 18). The data are read as column vs. row (e.g., BB vs. CON), with red branches indicating taxa more abundant in the column compared to the row label (e.g., Lachnospiraceae (bright red) relative abundance was higher in the BB-fed mice compared to the control, whereas Clostridiaceae (dark blue) was less abundant in the BRB-fed mice compared to those fed CB). CON, control; BB, bilberry; BC, black currant; EB, elderberry; CB, chokeberry; BRB, black raspberry; TC, tart cherry; CPs, cocoa polyphenols.

**Figure 5:**
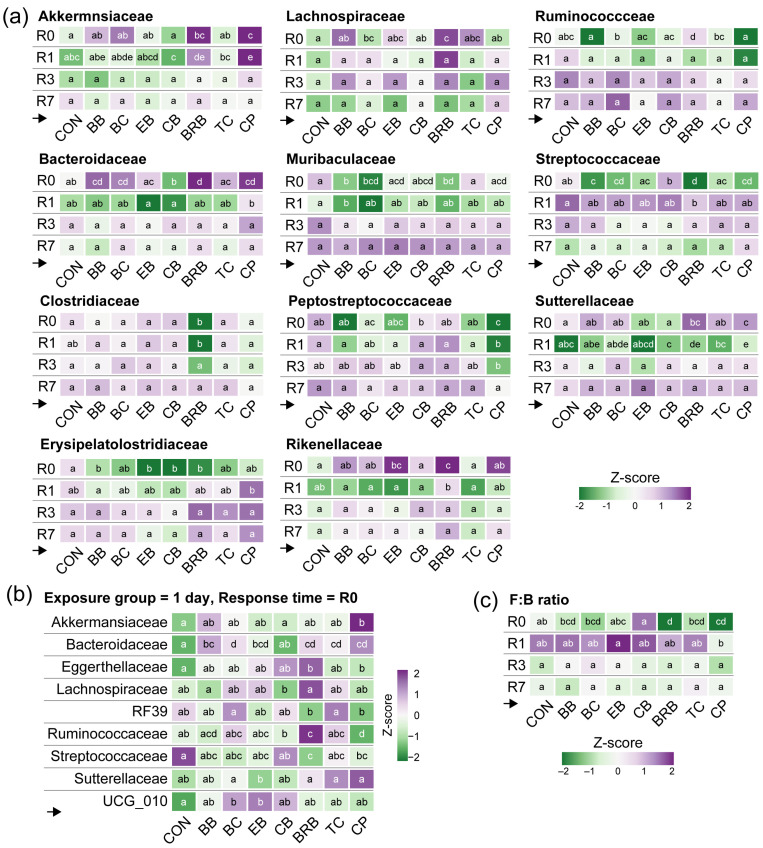
Effect of the supplement on the microbiome composition as a factor of the response time. (**a**) Heatmaps show the Z-scaled relative abundances of selected bacteria families by supplement within each response time (*n* = 16 to 18), controlling for the exposure group. (**b**) Heatmaps show the Z-scaled relative abundances for all the bacteria families significantly affected by the supplement, with exposure group = 1 day and response time = R0 (*n* = 6). (**c**) Heatmap showing the Z-scaled log_10_ Firmicutes-to-Bacteroidetes (F:B) ratio for the supplement groups within each response time. Different letters indicate that the supplement groups are significantly different (FDR *p* < 0.05) (within rows), as determined by MaAsLan2 multivariate analysis (**a**,**b**) or a generalized linear model with a Tukey post hoc test (**c**). The complete statistical results are available in File S2. Figures S10–S15 show the relative abundance data as individual Tukey box–whisker plots; Figures S16 and S17 show the F:B ratios for the main effects and all the subgroup analyses. CON, control; BB, bilberry; BC, black currant; EB, elderberry; CB, chokeberry; BRB, black raspberry; TC, tart cherry; CPs, cocoa polyphenols. The small black arrow → indicates that the heatmaps should be read across rows.

When examining the supplement effects within each exposure group (Q3), we observed scattered significances among the taxa but no exposure-dependent patterns (Figure 3a). Instead, strong differences appeared at early response times (Q4), particularly R0 (Figure 3b and Figure 4). For instance, the Streptococcaceae abundance was the lowest in the BRB-fed mice (2.74% vs. 10.36% in CON; *p* = 3.81 × 10^−7^) and significantly reduced in BC (*p* = 0.0035), CPs (*p* = 0.0049), and BB (*p* = 0.0387) (Figure 5a). These differences largely disappeared by R1. Additional examples of transient shifts include Muribaculaceae, which dropped sharply at R0 in BB (1.81%; *p* = 1.05 × 10^−4^), BC (2.37%; *p* = 0.0028), and BRB (2.32%; *p* = 1.25 × 10^−4^) relative to CON (5.13%), and Lachnospiraceae, which increased at R0 in BRB (16.5%; *p* = 0.0038) and BC (16.0%; *p* = 0.0038) compared to CON (12.5%) (Figure 5a). One of the few taxa to show short-term persistence was Peptostreptococcaceae. In the CP-fed mice, the abundance dropped significantly at R0 (5.20% vs. 13.0% in CON; *p* = 0.0028) and remained reduced at R1 (5.77% vs. 12.4%; *p* = 0.0014), although the differences disappeared by R3.

To determine how quickly the gut microbiome can respond to dietary interventions (*Q5*), we examined the microbial composition immediately following a single day of supplementation (Figure 5b). A single day of dietary exposure was sufficient to induce significant shifts in the microbiome composition, demonstrating the rapid responsiveness of gut bacterial communities to dietary changes. Akkermansiaceae and Bacteroidaceae were elevated in the CP-fed mice, while Streptococcaceae was reduced in the BRB- and CB-fed mice. These results confirm that the microbiome responds rapidly to even a single day of dietary supplementation, although most changes were not sustained.

Collectively, these findings demonstrate that dietary ACNs and CPs induce strong but largely transient changes in the bacterial community composition. While a few taxa showed short-term persistence, most effects diminished within a few days, emphasizing the microbiome’s capacity for rapid adaptation and the importance of continued intake to sustain compositional shifts.

### 3.3. Firmicutes-to-Bacteroidetes Ratio

The Firmicutes-to-Bacteroidetes (F:B) ratio varied significantly by exposure group, response time, and supplement (Figure S16, Table S3). The mice exposed for 7 days had a higher F:B ratio than those in the 1- or 3-day groups, suggesting that longer exposure or experimental duration altered the microbial composition (Figure S16a, Table S4). Across all the groups, the F:B ratio increased after supplementation, peaking at R1 and declining by R3 and R7 but never returning to the baseline (Figure S16b, Table S5), indicating a lasting microbiome shift. When averaged across exposure and response times, only CPs significantly differed from the control, showing a lower F:B ratio (*p* = 0.0290) (Figure S16c, Table S6).

The exposure duration (Q1) had a limited impact on the F:B ratio. For instance, the CB-fed mice showed a modest increase with longer exposures (Figure S16d), but this trend was not statistically significant across the board. Interestingly, even the control (CON) mice showed an unexplained dip at day 3, suggesting a time-related drift unrelated to supplementation. In contrast, the response time (Q2) exerted a clearer effect (Figure S16e). At R0, the F:B ratios increased significantly in several groups relative to the baseline, such as BB (*p* = 0.0301), EB (*p* = 0.0127), CB (*p* = 0.0193), and TC (*p* = 0.0412). These increases peaked at R1, where all the supplement groups differed significantly from the pre-DSS time point (Pre) (*p* < 0.05 for all the supplement groups), before trending downward by R3 and R7. Still, most groups remained slightly elevated at R7 compared to the baseline.

To evaluate whether the supplementation effects on the F:B ratio varied by exposure duration (Q3), we compared the supplements within each exposure group (Figure S17, Table S9). In the 1-day group, the CB-fed mice had a significantly higher F:B ratio than those fed CPs (*p* < 0.0001), BRB (*p* = 0.0164), and TC (*p* = 0.0489). No significant differences were observed in the 3- or 7-day groups, indicating that the supplementation effects were not strongly dependent on the exposure duration. When comparing the supplements within each response time (Q4) while excluding the exposure, the most pronounced differences occurred at R0 (Figure 5c, Table S10). At this point, the CB-fed mice had F:B ratios similar to those of the controls, while the BRB (*p* = 0.0019) and CP (*p* = 0.0085) groups showed significantly lower ratios. The CB-fed mice also had higher F:B ratios than all the other supplement groups (*p* < 0.05). By R1, only the CP-fed mice maintained a significantly lower F:B ratio compared to EB (*p* = 0.0217). No significant differences were observed at pre-supplementation, R3, or R7. At R0, after only one day of supplementation (Q5), several supplements induced rapid changes (Figure S17). The mice fed BB, BC, BRB, and CPs all had significantly lower F:B ratios than those of the CON (*p* < 0.05), while the CB-fed mice had the highest levels. These shifts occurred without longer exposures, highlighting the microbiome’s sensitivity to even short-term dietary interventions.

Together, these results demonstrate that the F:B ratio responds rapidly to ACN-rich and polyphenol supplementations, with the response time and supplement type having greater influence than the exposure duration. Although most effects were transient, CPs consistently induced a lower F:B ratio, suggesting a potentially more persistent impact on the microbial balance.

### 3.4. Alpha-Diversity of the Fecal Microbiomes

The alpha-diversity varied significantly by supplement, with minimal influence from the exposure duration or response time (Figure S18; Tables S12–S20). The Chao1 and Shannon diversities did not differ across the exposure groups, though interactions with the supplement indicated some variability. Across timepoints, Chao1 remained stable, while the Shannon diversity increased at R0 compared to Pre (*p* = 0.0490), returning to the baseline by R1. A significant exposure × response time interaction was detected, but no clear pairwise differences emerged. Among the supplements, Chao1 was higher in the BB-fed mice than in the controls (*p* = 0.0396), though not significantly different from the other supplements. The Shannon diversity was higher in the EB-fed mice than in CON (*p* = 0.0460) and CPs (*p* = 0.0003) and in BB (*p* = 0.0063) and BC (*p* = 0.0006) compared to CPs. Overall, supplementation modestly affected the alpha-diversity, with BB and EB showing the most notable increases.

The exposure duration (Q1) had the minimal impact on the alpha-diversity. No significant differences in the Chao1 or Shannon diversities were observed across the exposure groups for most supplements (Figure S19). One exception was CPs, where the Shannon diversity increased slightly with longer exposures, rising at 3 days (*p* = 0.0198) and 7 days (*p* = 0.0392) compared to 1 day, suggesting a mild exposure-dependent effect. No similar trends were observed for the ACN-rich supplements. For the response time (Q2), the diversity changes were generally transient. In the TC-fed mice, both the Chao1 and Shannon diversities increased at R0 (*p* = 0.0069 and *p* = 0.0009, respectively) but returned to the baseline by R1. A similar pattern was observed for EB, where the Shannon diversity trended higher at R0 (*p* = 0.0973) but declined thereafter. These results suggest early shifts in the microbial diversity that do not persist without continued supplementation.

**Figure 6:**
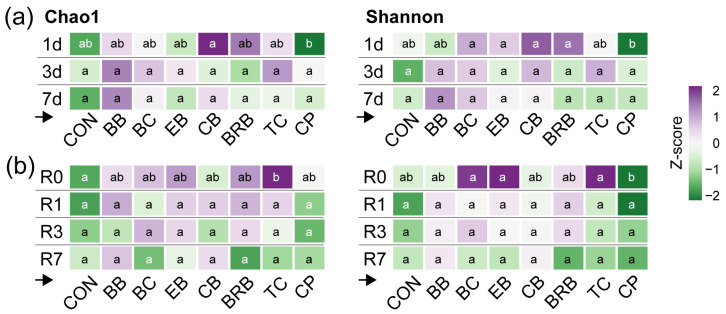
Effects of the supplements on the alpha-diversity as a factor of the (**a**) exposure group or (**b**) response time. Heatmaps show the Z-scaled Chao1 and Shannon alpha-diversity scores for the supplements within each exposure group (*n* = 27 to 30) or response time (*n* = 16 to 18). Different letters indicate that supplement groups are significantly different (FDR *p* < 0.05) (within rows), as determined by a generalized linear model with a Tukey post hoc test. CON, control; BB, bilberry; BC, black currant; EB, elderberry; CB, chokeberry; BRB, black raspberry; TC, tart cherry; CPs, cocoa polyphenols. The small black arrow → indicates that the heatmaps should be read across rows.

To evaluate whether the supplement effects on the alpha-diversity depended on the exposure duration (Q3), we analyzed the diversity within each exposure group (Figure S20). After 1 day of supplementation, the Chao1 diversity was higher in the EB-fed mice than in the CP-fed mice (*p* = 0.0407). The Shannon diversity was also higher in the BC, BRB, CB, and EB groups compared to CPs (all *p* < 0.01), though none differed from that in CON. No significant differences were observed among the supplements after 3 or 7 days of exposure, indicating that the diversity effects were more pronounced with brief supplementation. To assess the variation by response times (Q4), we compared the alpha-diversities across the supplements within each timepoint (Figure 6 and Figure S20). Differences emerged only at R0: The Chao1 diversity was higher in the TC-fed mice than in CON (*p* = 0.0028), and the Shannon diversity was higher in BC, EB, and TC compared to CPs (all *p* < 0.01) but not relative to CON. No differences were detected at later timepoints, suggesting that the supplement-induced changes in the diversity were limited to early responses.

To assess the immediate effects (Q5), we examined the alpha-diversity at R0 following a 1-day exposure (Figure S21). While the Chao1 diversity showed a non-significant trend toward higher richness in the TC-fed mice (*p* = 0.0856), the Shannon diversity was significantly higher in the EB-fed mice compared to BB (*p* = 0.0225) and CPs (*p* = 0.0003) and elevated in TC (*p* = 0.0071) and BC (*p* = 0.0048) relative to CPs. Still, none of the supplements significantly differed from CON. In summary, the alpha-diversity was modestly and transiently affected by ACN and CP supplementation, with the clearest effects observed at early response times. The CP-fed mice consistently showed lower diversities than several other groups, while BB, EB, and TC produced short-lived increases in microbial diversity, particularly in the Shannon index.

### 3.5. Beta-Diversity of the Fecal Microbiomes

The beta-diversity, assessed using unweighted and weighted UniFrac distances, revealed that the response time, supplement, and exposure duration all significantly influenced the microbial community composition (PERMANOVA *p* < 0.001) (Figure S22). However, the effect sizes were small in most cases, with *R*^2^ values below 0.1 for unweighted analyses, indicating that these variables explained only a limited proportion of the total variance. In contrast, the response time had a more substantial impact in the weighted UniFrac analysis, particularly reflecting the shift from pre-supplementation to R0 (*R*^2^ = 0.178, *p* = 0.001), followed by the gradual convergence of the community structure at later timepoints.

The exposure duration (Q1) had the minimal impact on the microbiome composition, as the exposure groups (1, 3, and 7 days) showed substantial overlap within each response time and supplement (Figures S23 and S24, Table S24). The pairwise PERMANOVA unweighted and weighted UniFrac tests were all insignificant (*R*^2^ < 0.1, *p* > 0.05), save for a marginal divergence for 7 days vs. 1 day of exposure to TC (*R*^2^ = 0.103, *p* = 0.003). Alternatively, the fecal microbiome profiles differed markedly for response times (*Q2*) for both the unweighted and weighted UniFrac beta-diversities (Table S25). To better visualize how the microbiome composition changed over time for each supplement, we plotted PCoA centroids for each response timepoint (from R0 to R7) relative to the pre-supplementation baseline.

**Figure 7:**
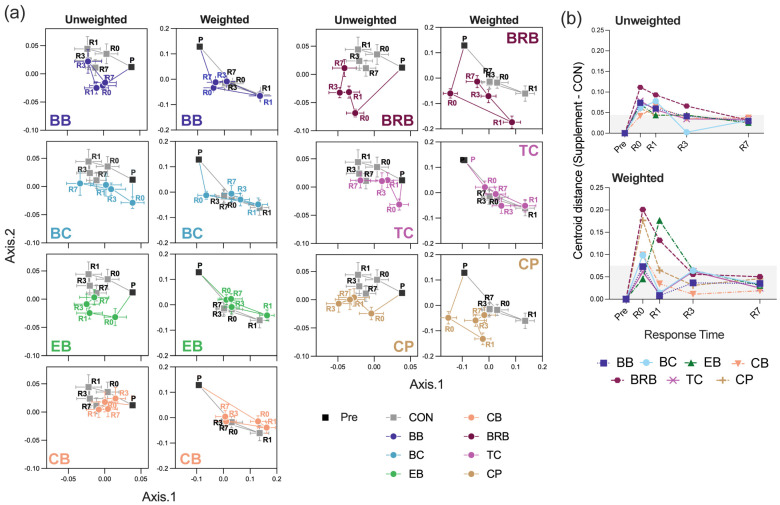
Temporal effects of anthocyanin- and cocoa-polyphenol-supplemented diets on the gut microbiome composition. (**a**) Principal coordinate analysis (PCoA) plots based on unweighted and weighted UniFrac distances for each supplement group (BB, BC, EB, CB, BRB, TC, and CPs) at response times Pre, R0, R1, R3, and R7, irrespective of the exposure group (*n* = 16 to 18). The data are shown as the Euclidean distance centroid for each supplement group at each response time, with error bars indicating SEMs for axis 1 and axis 2. Vectors indicate the progression of timepoints for each supplement group. Gray markers indicate response-time-matched control (CON) samples, and the baseline microbiome profile is labeled as “P”. (**b**) Line plots showing centroid distances between each supplement group and time-matched control (CON) samples across the response times (Pre, R0, R1, R3, and R7), based on unweighted (top) and weighted (bottom) UniFrac metrics. Distance values exceeding 1 SD of the Pre mean centroid (region colored in gray) were considered as biologically relevant. CON, control; BB, bilberry; BC, black currant; EB, elderberry; CB, chokeberry; BRB, black raspberry; TC, tart cherry; CPs, cocoa polyphenols.

In both the unweighted and weighted UniFrac plots, the largest centroid shifts were observed at R0, indicating an immediate response to supplementation (Figure 7a,b). These shifts were especially pronounced for BRB, EB, and CPs in both distance metrics, suggesting strong early changes in the microbial composition. For some supplements, such as BB and BC, the shift was more evident in unweighted UniFrac, suggesting a greater influence of rarer taxa. In contrast, weighted UniFrac revealed more pronounced shifts for CB and CPs, indicating changes among more abundant community members. Notably, the microbiome composition of the CON-fed mice also shifted over time, indicating that temporal drift occurred, even in the absence of dietary intervention. This underscores the importance of comparing each supplement group directly to its corresponding control at each timepoint. As with other endpoints in this study, longer exposure did not consistently produce greater shifts in microbiome populations vs. CON. These observations suggest that microbiome responses to supplementation may occur rapidly and do not require prolonged exposure to be detectable.

**Figure 8:**
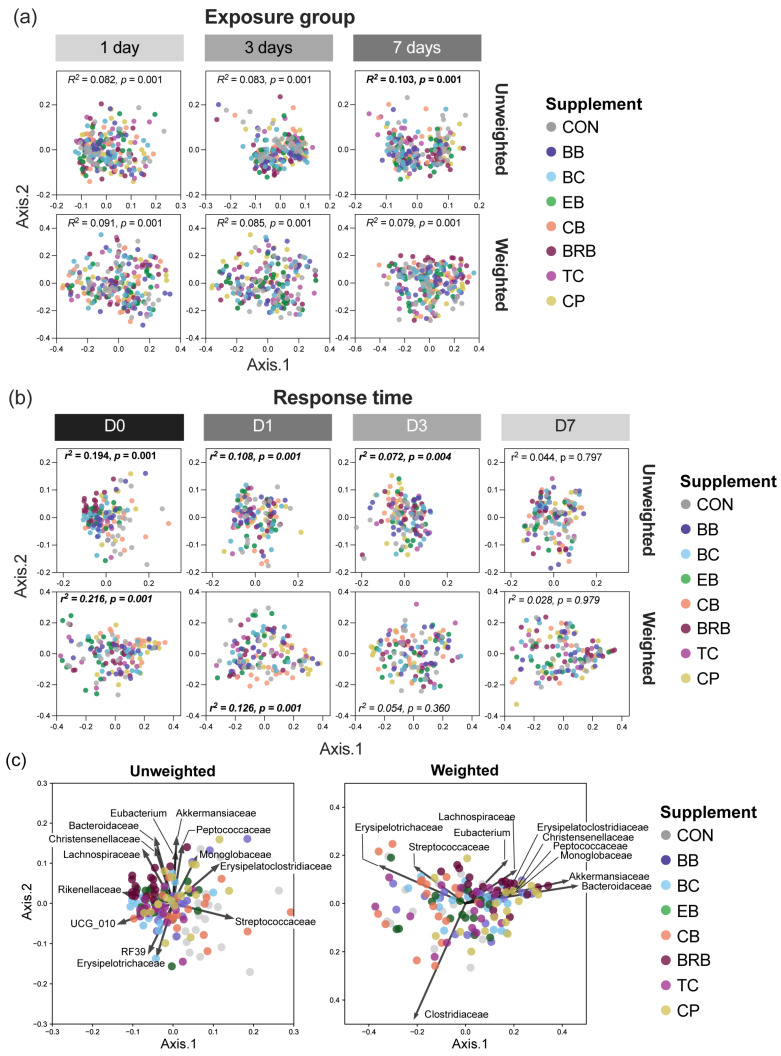
Beta-diversities of mouse fecal microbiomes for supplements within each exposure group or response time. The principal coordinate plots show the weighted or unweighted UniFrac distances for (**a**) supplements within each exposure group (*n* = 27 to 30) or (**b**) supplements within each response time (*n* = 16 to 18). The PERMANOVA *R*^2^ and *p*-values are shown. FDR-adjusted *p*-values of <0.05 were considered as statistically significant, with a meaningful effect size set at *R*^2^ > 0.1. PEMANOVA pairwise comparison results are provided in Tables S26 and S27. (**c**) PCoA biplots at response time = R0, displaying family-level vectors overlaid on unweighted (left) and weighted (right) UniFrac PCoA plots. Biplots were created using Spearman correlations between the first two principal coordinates and the relative abundance of each taxon across all the samples at response time R0. Arrows indicate taxa contributing to differences in the microbial composition. “Eubacterium_…” indicates the bacteria family Eubacterium_coprostanoligenes_group. CON, control; BB, bilberry; BC, black currant; EB, elderberry; CB, chokeberry; BRB, black raspberry; TC, tart cherry; CPs, cocoa polyphenols.

We next considered the effect of the supplement within each exposure group (*Q3*) and found little evidence of strong clustering by exposure group for either unweighted or weighted UniFrac, with *R*^2^ < 0.103 for all the comparisons (Figure 8a, Table S26). We therefore focused on the response time (Q4), where the clearest differences among the supplements emerged at R0 (Figure 8b, Table S27). Unweighted UniFrac showed significant separations of BRB, BC, EB, and TC from CON (*R*^2^ > 0.15, *p* = 0.001), with CB clustering with CON and the CP group intermediate. BRB diverged significantly from CB, CPs, EB, and TC (*R*^2^ = 0.115–0.334, *p* < 0.008). At R1, some distinctions persisted, most notably between BRB and CPs (*R*^2^ = 0.143, *p* = 0.007) and between CB and CPs (*R*^2^ = 0.199, *p* = 0.008). No significant differences were observed at R3 or R7 (*R*^2^ < 0.1 or *p* > 0.05).

Weighted UniFrac analyses similarly showed clear separations at R0. BRB significantly differed from CB (*R*^2^ = 0.334, *p* = 0.004), CPs (*R*^2^ = 0.115, *p* = 0.005), EB (R^2^ = 0.229, *p* = 0.004), and TC (*R*^2^ = 0.200, *p* = 0.004). Significant differences also emerged between CB and CPs (*R*^2^ = 0.295, *p* = 0.004) and between BC and CB (*R*^2^ = 0.190, *p* = 0.004). At R1, BRB remained distinct from CPs (*R*^2^ = 0.143, *p* = 0.007) and EB (*R*^2^ = 0.132, *p* = 0.007), and CB differed from CPs (*R*^2^ = 0.199, *p* = 0.008). No significant differences were detected at R3 or R7, with all the comparisons below the *R*^2^ > 0.1 threshold.

The taxon abundance played a key role in driving beta-diversity patterns across the supplement groups at R0, with notable differences observed between unweighted and weighted UniFrac analyses (Figure 8c). In unweighted UniFrac, BRB, EB, BC, and BB were associated with Rikenellaceae, Lachnospiraceae, and Bacteroidaceae, while CON and CB aligned with Streptococcaceae and Erysipelotrichaceae. TC grouped with UCG_010 and Monoglobaceae; Eubacterium was more broadly distributed. In the weighted analysis, Lachnospiraceae and Eubacterium were the key drivers for BRB and CPs, while Clostridiaceae characterized EB, BB, CB, TC, and CON. Erysipelotrichaceae was prominent in CB. Overall, BRB, EB, and BC were associated with higher relative abundances of Rikenellaceae and Lachnospiraceae, while CON and CB reflected greater abundances of Streptococcaceae and Erysipelotrichaceae, highlighting diet-specific shifts in both rare and abundant taxa.

To test for rapid responses (Q5), we assessed the beta-diversity after 1 day of supplementation at response time R0 (Figure S25). The microbial communities were clearly separated by supplement, particularly for weighted UniFrac (*R*^2^ = 0.370, *p =* 0.001). Significant differences were detected between multiple supplements, with BRB, CPs, and EB exhibiting the most distinct profiles. For example, BRB differed from CB (*R*^2^ = 0.299, *p =* 0.047), CPs (*R*^2^ = 0.243, *p =* 0.067), and CON (*R*^2^ = 0.309, *p =* 0.048) by weighted UniFrac. CPs also differed from CB (*R*^2^ = 0.406, *p =* 0.047) and BC (*R*^2^ = 0.262, *p =* 0.047). These findings confirm that microbiome shifts can occur rapidly after a single day of supplementation, with several supplement groups already diverging significantly from each other and from the controls.

### 3.6. Anthocyanin Structure and Microbiome Changes

**Figure 9:**
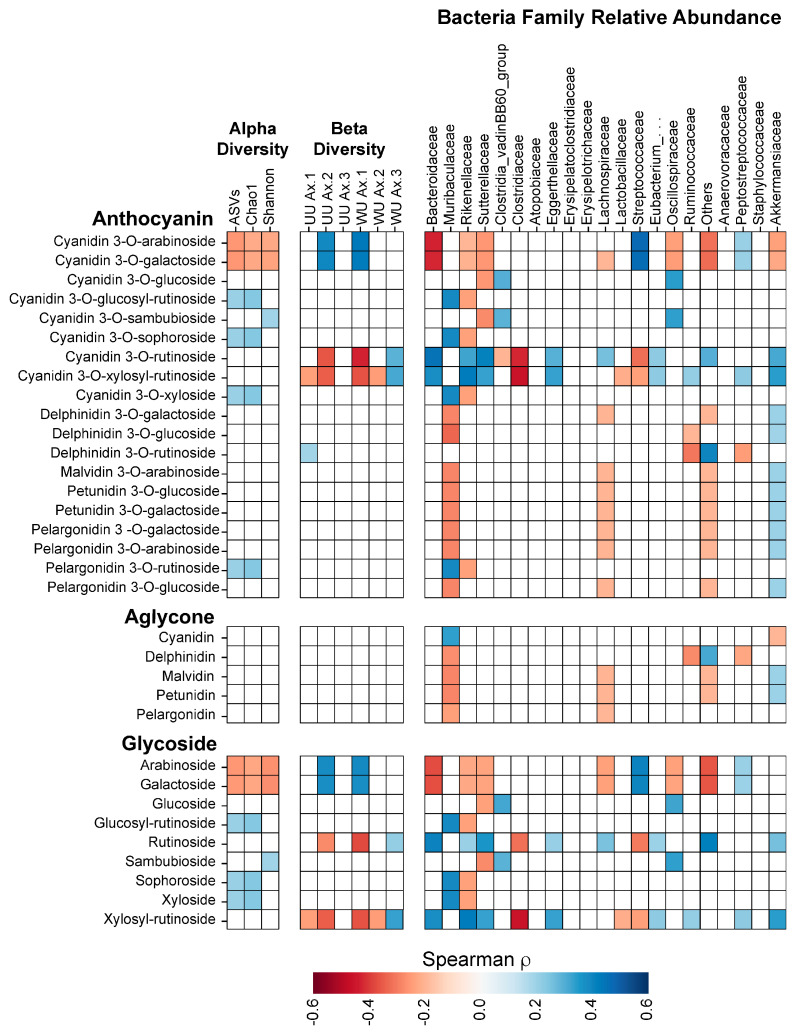
Correlation between anthocyanin profiles and microbiome metrics. Spearman correlation coefficients (ρ) were calculated between the relative abundances of individual anthocyanins (grouped by full compound, aglycone, or glycoside structure) and various microbiome outcomes. Correlated outcomes include the alpha-diversity (observed ASVs, Chao1 richness, and Shannon diversity), beta-diversity (principal coordinate axes from unweighted (UU) and weighted (WU) UniFrac distance matrices), and the relative abundances of selected bacterial families. Only statistically significant correlations (*p* < 0.05) are shown. The color intensity indicates the strength and direction of the correlation, with red indicating negative and blue indicating positive correlations. “Eubacterium_…” indicates the bacteria family Eubacterium_coprostanoligenes_group.

According to the HPLC analysis, each fruit powder, except for CPs and CON, used to supplement the TWD has a different profile of anthocyanins (Figure S1a), each of which has a unique combination of the polyphenol–aglycone structure with different glycosides. To determine whether microbial responses were driven by the aglycone or sugar moiety, we correlated individual anthocyanins with bacterial family abundances (Figure 9). Glycosides showed stronger and more consistent associations than aglycones. Notably, xylosyl-rutinoside and rutinoside were negatively correlated with Clostridiaceae and Streptococcaceae and positively associated with Ruminococcaceae, patterns most evident in BRB and BC diets. Bacteroidaceae was negatively associated with cyanidin 3-O-arabinoside and 3-O-galactoside, found in CB and BB; though, only CB showed the expected reduction. Akkermansiaceae negatively correlated with cyanidin, aligning with reduced abundances of EB and TC. Among aglycones, delphinidin was negatively associated with Ruminococcaceae, potentially explaining its lower abundances in BB and BC. Overall, the glycoside composition appeared to play a dominant role in shaping the microbiota, with aglycones exerting more modest, supplement-specific effects.

Glycosides were also stronger predictors of the alpha-diversity (Figure 9). Arabinoside and galactoside were negatively correlated with all the diversity indices, consistent with lower diversities in CB and BB at R0. In contrast, glucosyl-rutinoside, sophoroside, and xyloside were positively associated with richness, aligning with TC, the only supplement containing all three. Sambubioside, present in EB, was the only sugar positively linked to the Shannon diversity. Aglycones showed no meaningful associations with any alpha-diversity metric.

Similarly, glycosides, not aglycones, were significantly correlated with the beta-diversity (Figure 9). In unweighted UniFrac, arabinoside and galactoside positively correlated with axis 2, while rutinoside and xylosyl-rutinoside showed negative correlations. In weighted UniFrac, rutinoside and xylosyl-rutinoside were also negatively associated with axis 1, reflecting the microbial shifts seen in BC, BRB, and TC. These findings suggest that glycosylation patterns, rather than the polyphenol backbone, primarily drive changes in the microbial diversity and community structure.

## 4. Discussion

**Figure 10:**
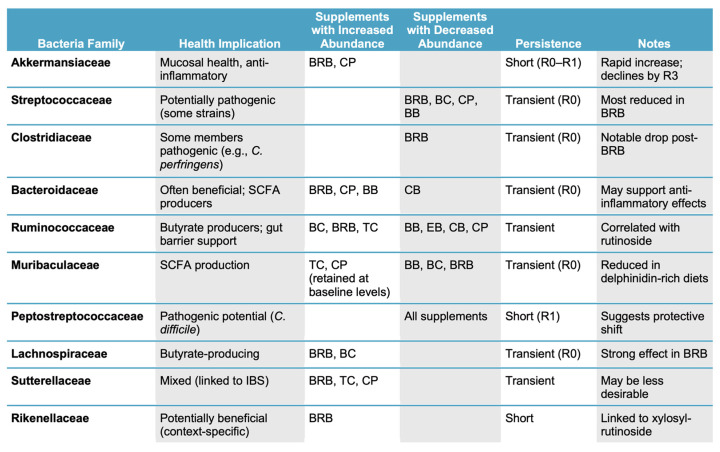
Summary of the primary findings for the effect of the supplement on the fecal microbiome composition, focusing on the duration of the response and health implications. CON, control; BB, bilberry; BC, black currant; EB, elderberry; CB, chokeberry; BRB, black raspberry; TC, tart cherry; CPs, cocoa polyphenols; R, response time; IBS, irritable bowel syndrome; SCFA, short-chain fatty acid.

The gut microbiome plays critical roles in regulating intestinal health and inflammation, and ACN-rich foods have emerged as promising modulators of microbial composition. However, most preclinical studies rely on extended periods of supplementation and rarely assess how rapidly microbiome shifts occur or whether they persist after dietary exposure ends. In this study, we systematically characterized short-term microbiome responses to a panel of structurally diverse ACN-rich fruit powders, delivered for just 1, 3, or 7 days, in the context of a Western-style diet. Remarkably, a single day of supplementation was sufficient to induce pronounced changes in the microbial community structure, including shifts in the β-diversity and the relative abundances of families such as *Akkermansiaceae* and *Bacteroidaceae*. These effects were largely transient, with most microbial communities reverting toward the baseline within 3 to 7 days post intervention. The duration and specificity of the microbial shifts varied by supplement and appeared to be influenced by anthocyanin glycosylation patterns. A diet enriched in CPs, which contains flavanols rather than anthocyanins, also induced rapid shifts, supporting the idea that multiple polyphenol classes can modulate the microbiome on short timescales. Together, these findings highlight the plasticity of the microbiome and its sensitivity to short-term dietary input (Figure 10).

The microbiome shifts were not only rapid but also diet- and taxon-specific, with variable patterns depending on the exposure duration. The analysis of the centroid distances suggested that 1-day exposures often triggered the greatest divergence from the control at early response times, while 3- and 7-day exposures showed more modest changes, possibly reflecting microbial adaptation or stabilization. The relative abundance data revealed that some bacterial families responded exclusively to short-term exposure. For example, Sutterellaceae exhibited elevated abundance only after 1 day, consistent with a fast but transient response. In contrast, other bacteria families, such as Atopobiaceae, Monoglobaceae, and Erysipelotrichaceae, did not show changes until after 3 days of supplementation, and in some cases (e.g., the BRB-fed mice), persisted through R7, suggesting a cumulative effect of sustained dietary input. The BB and BRB diets, for example, increased Erysipelotrichaceae after 3 days, with BRB effects continuing at 7 days. The Lactobacillaceae abundance was elevated in the BC group across all the exposure durations, suggesting that some taxa may be less sensitive to the exposure time and instead respond consistently to the supplement itself. These results highlight that while some bacteria respond immediately but transiently, others require sustained exposure for establishment. Therefore, the rapid response of the gut microbiome to these ACN-rich diets highlights its high plasticity, indicating that microbial communities can quickly adapt to dietary inputs and suggesting that certain bacterial taxa can expand rapidly when exposed to anthocyanins, potentially due to their ability to metabolize polyphenols and produce bioactive metabolites.

We next examined whether the microbiome changes observed in response to ACN-rich diets persisted after the dietary intervention ceased. In line with our hypothesis, the fecal microbiomes of the mice fed most of the supplemented diets reverted to a composition resembling that of the baseline by 7 days post intervention (R7), suggesting that the diet-driven microbiome shifts were transient. Among the ACN-rich supplements, BRB consumption led to the most sustained effects, with a distinct microbial composition still evident at 1 or 3 days after the exposure ended. However, this effect dissipated by 7 days post exposure. BB and EB also induced noticeable shifts at R1, but these were no longer apparent by R3. Interestingly, despite the marked and rapid microbiome changes seen with CPs immediately following exposure, these populations changes tended not to persist beyond 1 day post exposure, further emphasizing the short-lived nature of the microbial response to this diet with a differing polyphenol profile, enriched in (-)-epicatechin.

In line with our findings, a longitudinal intervention study showed that individuals fed black elderberry extract for three weeks showed a shifted fecal microbiome composition compared to that of the baseline [30]. However, during the washout period, the shift in the microbiome composition lasted for one week and then resolved similarly to the baseline composition for the remaining two weeks in the study. Also, Chacar et al. [31] reported that the dietary intake of a mixture of phenolic compounds for 14 months modulated the gut microbiome in rats, including the suppression of potentially harmful taxa, like *Clostridium (cluster I)*, and the enrichment of beneficial bacteria, such as *Bifidobacterium*. However, the researchers did not assess whether the microbial changes persisted after the dietary intervention was withdrawn. Collectively, these findings support the importance of consistent ACN-rich food consumption to maintain microbiome alterations and suggest that short-term dietary interventions may be insufficient to induce lasting changes in gut microbial communities.

The correlation analyses in this study provided insight into how differences in anthocyanin profiles across the tested diets influenced the gut microbiome. Among the diets, BRB induced the most robust shifts in the microbial composition and was positively correlated with the abundances of several bacterial families that appeared to drive this response. These effects were associated with BRB’s high content of rutinoside sugars, which showed positive correlations with bacterial families such as Bacteroidaceae, Lachnospiraceae, and Rikenellaceae. A similar pattern was observed for the BC diet, which also contains elevated levels of rutinoside. Notably, the aglycone component of the anthocyanins did not appear to influence the alpha- or beta-diversity metrics.

In contrast to the ACN-rich diets, the CP diet contains polyphenolic compounds and proanthocyanidins, primarily catechins, rather than anthocyanins. While CPs also altered the microbiome composition, the effects may have been driven by different microbial pathways, favoring bacteria that metabolize flavanols rather than anthocyanins. Although our correlation analysis focused specifically on the anthocyanin structure, considering both aglycone and sugar moieties, it is likely that other components of the food matrix also contributed to the observed differences in the microbial responses. These findings highlight the importance of sugar conjugation in anthocyanins and suggest that dietary effects on the microbiome may extend beyond the polyphenols themselves to include broader interactions with the food matrix.

Cocoa polyphenols, rich in flavanols and proanthocyanidins, such as (–)-epicatechin, have been previously shown to modulate the gut microbiota in both animals and humans. In vitro and in vivo studies have reported that CPs stimulate the growth of beneficial bacteria, like *Lactobacillus* and *Bifidobacterium*, while reducing pathogenic *Clostridium* species [32,33]. These effects accompany improved gut barrier function, reductions in inflammatory markers, such as C-reactive protein, and enhanced production of short-chain fatty acids. In our study, CP supplementation produced similar early increases in Akkermansiaceae and Bacteroidaceae, aligning with its recognized prebiotic effects. However, unlike anthocyanin-rich diets, CPs’ impact was not sustained beyond R1 and did not enrich taxa like Lachnospiraceae or Erysipelotrichaceae. This pattern is consistent with the structural specificity of polyphenol-driven microbial modulation, where CPs’ flavanol-rich composition may preferentially target the initial responders but lacks durable effects on fiber- and glycoside-metabolizing bacteria that anthocyanins influence.

Several bacterial families showed distinct responses to ACN-rich diets, particularly at the R0 timepoint, where the most pronounced shifts were observed. Among the most striking was the increase in Akkermansiaceae, especially in the mice fed the BRB and CP diets. This family, which includes *A. muciniphila*, has been extensively studied for its roles in maintaining the mucosal barrier integrity and gut homeostasis by stimulating mucin production [34]. Similar increases in *Akkermansia* have been reported in mice fed cranberry powder, supporting its association with beneficial gut modulation [35]. These findings align with our previous research, in which BRB supplementation increased Akkermansiaceae abundance in mice fed a healthy diet prior to colitis induction, though, interestingly, this was not observed in the context of a Western diet [24]. However, these two studies employed different designs, which could explain the contradictory finding.

Bacteroidaceae abundance was also elevated at R0 in several groups, including BRB, CPs, TC, and BC. Members of this family, such as *Bacteroides thetaiotaomicron*, have demonstrated anti-inflammatory effects in preclinical models of IBD [36]. These results are consistent with studies showing that berry-based diets can enhance Bacteroidaceae abundance [37], further supporting the potential for ACN-rich foods to beneficially modulate the microbiota. In contrast, Clostridiaceae, specifically, *Clostridium sensu stricto 1*, a group of bacteria that includes pathogenic species, like *C. perfringens* and *C. tetani* [38,39], was reduced in the BRB group, potentially reflecting a health-promoting shift.

Muribaculaceae, a bacteria family linked to short-chain fatty acid production and gut barrier maintenance [40], was largely reduced across the diets at response time R0 compared to CON, with the exception of the CP and TC groups, which retained higher abundances. This may be due to differences in the ACN sugar composition; our correlation analysis indicated that cyanidin-3-O-glucosyl-rutinoside was positively associated with Muribaculaceae levels, while delphinidin-rich diets (e.g., BB and BC) showed reduced abundances, consistent with earlier findings [41].

Ruminococcaceae, another beneficial family important for butyrate production and intestinal health [42], was elevated in the BC, BRB, and TC groups at R0. These changes correlated with higher rutinoside and glucosyl-rutinoside contents, which were positively linked to Ruminococcaceae abundance in our correlation analysis [43]. Lower levels were seen in BB, EB, CB, and CPs, possibly due to negative associations with delphinidin. Peptococcaceae, though less well characterized, also increased across all the diets; prior research has linked polyphenol-rich extracts, such as those from Chinese bayberry leaves, with increased Peptococcaceae levels [44]. On the other hand, Peptostreptococcaceae, which includes opportunistic pathogens, like *Clostridioides difficile*, was significantly reduced at R1 in all the diet groups. Given this family’s association with IBD and colorectal cancer [45,46,47], its reduction may signal a protective microbial response to ACN-rich diets.

Lastly, the correlation analysis identified a positive association between the glycoside xylosyl-rutinoside and the abundance of Rikenellaceae, particularly in the microbiomes of the mice fed BRB. Prior studies using blueberry or cranberry extracts, which lack xylosyl-rutinoside, reported reductions in Rikenellaceae, supporting the importance of the glycoside composition in modulating this family [48,49,50]. Notably, a study using black goji berry (*Lycium ruthenicum* Murray), which contains cyanidin 3-O-xylosyl-rutinoside, observed a similar increase in the Rikenellaceae abundance [51]. These findings suggest that xylosyl-rutinoside may promote growth of this bacterial family. In contrast, Sutterellaceae was elevated in the fecal microbiomes of the mice provided BRB, TC, and CPs, with the genus *Parasutterella* specifically identified. This genus has been previously linked to irritable bowel syndrome (IBS) and may have mixed implications for gut health [52]. Considered all together, these findings suggest that the observed microbiome shifts include both health-promoting and potentially protective bacterial taxa and that specific anthocyanin structures, particularly sugar moieties, may play a key role in shaping microbial communities in response to dietary polyphenols.

Several limitations of this study warrant consideration. First, we observed unintended shifts in the microbiome of the control group over time, potentially due to feed intake variability or stress from cage transition. Time-matched CON controls were essential to isolate supplement effects from the baseline drift. Second, the study was conducted in healthy male mice only, which limits generalizability given known sex-based microbiome differences. Future work should incorporate both sexes to better reflect the spectrum of host responses. Third, the study focused on the taxonomic composition using 16S rRNA sequencing, which provides a taxonomic resolution commonly limited to higher ranks (e.g., genus or family) but does not capture the microbial function. Metagenomics or metabolomics would offer deeper insights into metabolic outputs and host interactions. Finally, our reliance on relative abundance data may obscure shifts in the absolute microbial loads.

Translational relevance is also a key consideration. Although the anthocyanin doses were standardized across the diets, these levels may exceed what is typically achieved through whole-food consumption. Some supplements, like BRB, were whole powders, while others were extracts, further complicating extrapolation to human diets. Importantly, this study was designed as the first step in a broader research program. Most preclinical studies employ prolonged, continuous dosing, unlike human eating-habits, where ACN-rich foods are consumed intermittently and variably. Our aim was to identify supplements that produce robust and/or complementary microbiome effects and to evaluate whether brief exposure could drive lasting microbial change. The findings here suggest that most effects are rapid but transient, raising questions about the efficacy of inconsistent intakes. Future studies will evaluate selected supplements alone and in combination in models of colitis or colorectal cancer, using feeding regimens that mimic realistic dietary patterns. These studies will incorporate host-level measures, including inflammatory markers, short-chain fatty acids, and histology, to assess functional consequences of microbiome modulation.

## 5. Conclusions

In summary, this study provides new insight into how short-term supplementation with anthocyanin-rich fruit powders rapidly alters the gut microbiome composition in the context of a Western-style diet. Significant changes in the microbial diversity and relative abundances emerged within 24 h of exposure, underscoring the high responsiveness of the gut microbiota to dietary polyphenols. However, these shifts were largely transient, with most communities reverting toward the baseline within a week of supplement withdrawal. The structural features of the anthocyanins, particularly the glycosylation patterns, influenced both the magnitude and persistence of the response, with whole-food BRB powder producing the most sustained effects. These findings emphasize the importance of consistent intake to maintain microbiome modulation and suggest that intermittent consumption may be insufficient for lasting benefits. Looking forward, these results establish a foundation for targeted dietary strategies that leverage polyphenol structure–activity relationships. Future studies will assess host-level impacts and explore whether strategic combinations or dosing patterns can support long-term gut health in disease-relevant contexts.

## Data Availability

Supporting sequencing data for this manuscript are available to the public at the Utah State University Digital Commons repository, https://doi.org/10.26078/jhzh-ef50 (deposited on 15 April 2025). Available files include the .txt mapping file with sample attribute information, the .csv file with 16S rRNA sequence count data with ASV identifiers, the .csv file with taxonomy mapped to the ASV identifier, and the phylogenetic tree file. All the other data are contained within the article or the accompanying Supplementary Material.

## Supplementary Materials

The following supporting information can be downloaded at https://www.mdpi.com/article/10.3390/nu17132201/s1, Figure S1: Chemical structures of selected anthocyanins; Figure S2: HPLC PDA analysis of anthocyanin-rich berry powders; Figure S3: Schematic illustrating stepwise analysis scheme aligned with overarching experimental questions; Figure S4: Outlier analysis of all the microbiome samples using alpha-diversity; Figure S5: Energy intake, bodyweight gain, final bodyweight, body composition, and relative cecal content weights of the mice; Figure S6: Rarefaction curve analysis by experimental diet; Figure S7: Taxonomic classification of mouse fecal bacteria; Figure S8: Heatmap of bacterial family-level composition in mouse fecal samples; Figure S9: Main effects of exposure group, response time, and supplement on relative abundance of bacteria families; Figure S10: Relative abundances of Akkermansiaceae and Bacteroidaceae for each exposure group, response time, and supplement; Figure S11: Relative abundances of Clostridiaceae and Erysipelatoclostridiaceae for each exposure group, response time, and supplement; Figure S12: Relative abundances of Lachnospiraceae and Muribaculaceae for each exposure group, response time, and supplement; Figure S13: Relative abundances of Peptostreptococcaceae and Rikenellaceae for each exposure group, response time, and supplement; Figure S14: Relative abundances of Ruminococcaceae and Streptococcaceae for each exposure group, response time, and supplement; Figure S15: Relative abundance of Sutterellaceae for each exposure group, response time, and supplement; Figure S16: Firmicutes-to-Bacteroidetes (F:B) ratio for exposure group, response time, and supplement; Figure S17: Firmicutes-to-Bacteroidetes (F:B) ratio for supplement within exposure group and response time; Figure S18: Alpha-diversity for exposure group, response time, and supplement; Figure S19: Alpha-diversity for exposure group or response time within each supplement group; Figure S20: Alpha-diversity for supplement within exposure group and response time; Figure S21: Alpha-diversity for supplement with exposure group = 1 day and response time = R0; Figure S22: UniFrac beta-diversity of mouse fecal microbiomes for exposure group, response time, or supplement; Figure S23: Unweighted UniFrac beta-diversity of mouse fecal microbiomes for exposure group, response time, or supplement; Figure S24: Weighted UniFrac beta-diversity of mouse fecal microbiomes for exposure group, response time, or supplement; Figure S25: Beta-diversity of mouse fecal microbiomes for supplement with exposure group = 1 day and response time = R0; Table S1: Measured anthocyanin contents for selected berry supplements; Table S2: Formulation of experimental diets; Table S3: *P*-values for main effects and interactions for all the experimental factors for F:B ratio; Table S4: Adjusted *p*-values for pairwise comparisons among exposure groups for F:B ratio, irrespective of response time or supplement; Table S5: Adjusted *p*-values for pairwise comparisons among response times for F:B ratio, irrespective of exposure or supplement; Table S6: Adjusted *p*-values for pairwise comparisons among supplement groups for F:B ratio, irrespective of exposure group or response time; Table S7: Adjusted *p*-values for pairwise comparisons among exposure groups within each supplement group for F:B ratio (Q1); Table S8: Adjusted *p*-values for pairwise comparisons among response times within each supplement group for F:B ratio (Q2); Table S9: Adjusted *p*-values for effect of supplement within each exposure group for F:B ratio (Q3); Table S10: Adjusted *p*-values for effect of supplement within each response time for FB ratio (Q4); Table S11: Adjusted *p*-values for effects of supplement for exposure group = 1 day and response time = R0 for F:B ratio (Q5); Table S12: *p*-values for main effects and interactions of all the experimental factors for alpha-diversity; Table S13: Adjusted *p*-values for pairwise comparisons among exposure groups for alpha-diversity, irrespective of response time or supplement; Table S14: Adjusted *p*-values for pairwise comparisons among response times for alpha-diversity, irrespective of exposure group or supplement; Table S15: Adjusted *p*-values for pairwise comparisons among supplement groups for alpha-diversity, irrespective of exposure group or response time; Table S16: Adjusted *p*-values for pairwise comparisons among exposure groups within each supplement group for alpha-diversity (Q1); Table S17: Adjusted *p*-values for pairwise comparisons among response times within each supplement group for alpha-diversity (Q2); Table S18: Adjusted *p*-values for effect of supplement within each exposure group for alpha-diversity (Q3); Table S19: Adjusted *p*-values for effect of supplement within each response time for alpha-diversity (Q4); Table S20: Adjusted *p*-values for effects of supplement for exposure group = 1 day and response time = R0 for alpha-diversity (Q5); Table S21: *R*^2^ and adjusted *p*-values for pairwise comparisons among exposure groups for UniFrac beta-diversity, irrespective of response time or supplement; Table S22: *R*^2^ and adjusted *p*-values for pairwise comparisons among response times for UniFrac beta-diversity, irrespective of exposure group or supplement; Table S23: *R*^2^ and adjusted *p*-values for pairwise comparisons among supplements for UniFrac beta-diversity, irrespective of exposure group or response time; Table S24: *R*^2^ and adjusted *p*-values for beta-diversity pairwise PERMANOVA tests for exposure group within each supplement group (Q1); Table S25: *R*^2^ and adjusted *p*-values for beta-diversity pairwise PERMANOVA tests for response time within each supplement (Q2); Table S26: *R*^2^ and adjusted *p*-values for beta-diversity pairwise PERMANOVA tests for supplement within each exposure group (Q3); Table S27: *R*^2^ and adjusted *p*-values for beta-diversity pairwise PERMANOVA tests for supplement within each response time (Q4); Table S28: *R*^2^ and adjusted *p*-values for beta-diversity pairwise PERMANOVA tests for supplement with exposure group = 1 day and response time = R0 (Q5); File S1: Microbiome count data.xlsx (https://doi.org/10.26078/jhzh-ef50); File S2: MetagenomeSeq statistics.xlsx (https://doi.org/10.26078/jhzh-ef50).
