# Supplementary material for: Dynamic Microbiome Responses to Structurally Diverse Anthocyanin-Rich Foods in a Western Diet Context
Source: Nutrients. 2025 Jul 1;17(13):2201. doi: 10.3390/nu17132201 (PMC12252082; doi:10.3390/nu17132201)
Supplement: Supplementary file 1 [file nutrients-17-02201-s001.zip › nutrients-3701180-supplementary.pdf]

Supplementary Material

Dynamic Microbiome Responses to Structurally Diverse Anthocyanin-Rich Foods in a Western Diet Context

Mohammed F. Almatani, Giovanni Rompato, Eliza C. Stewart, Marcus Hayden, Jeremy Case, Samuel Rice, Korry J. Hintze and Abby D. Benninghoff*

* Correspondence: abby.benninghoff@usu.edu; Tel.: +1-435-797-8649

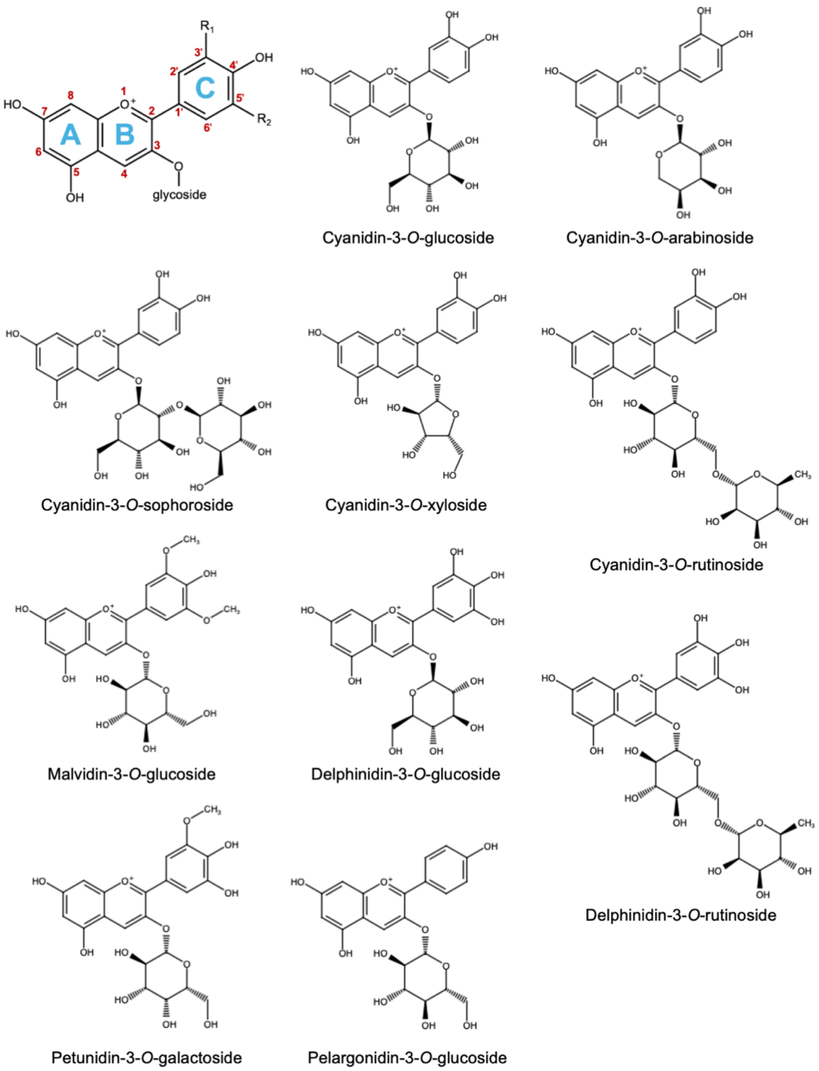

Figure S1. Chemical structures of selected anthocyanins. *Top left*, generic structure for anthocyanin highlighting the three-ring structure (A, B, and C), with functional groups R1 and R2 on the C ring and various glycosides contributing to structural diversity. Other typical anthocyanin structures are shown.

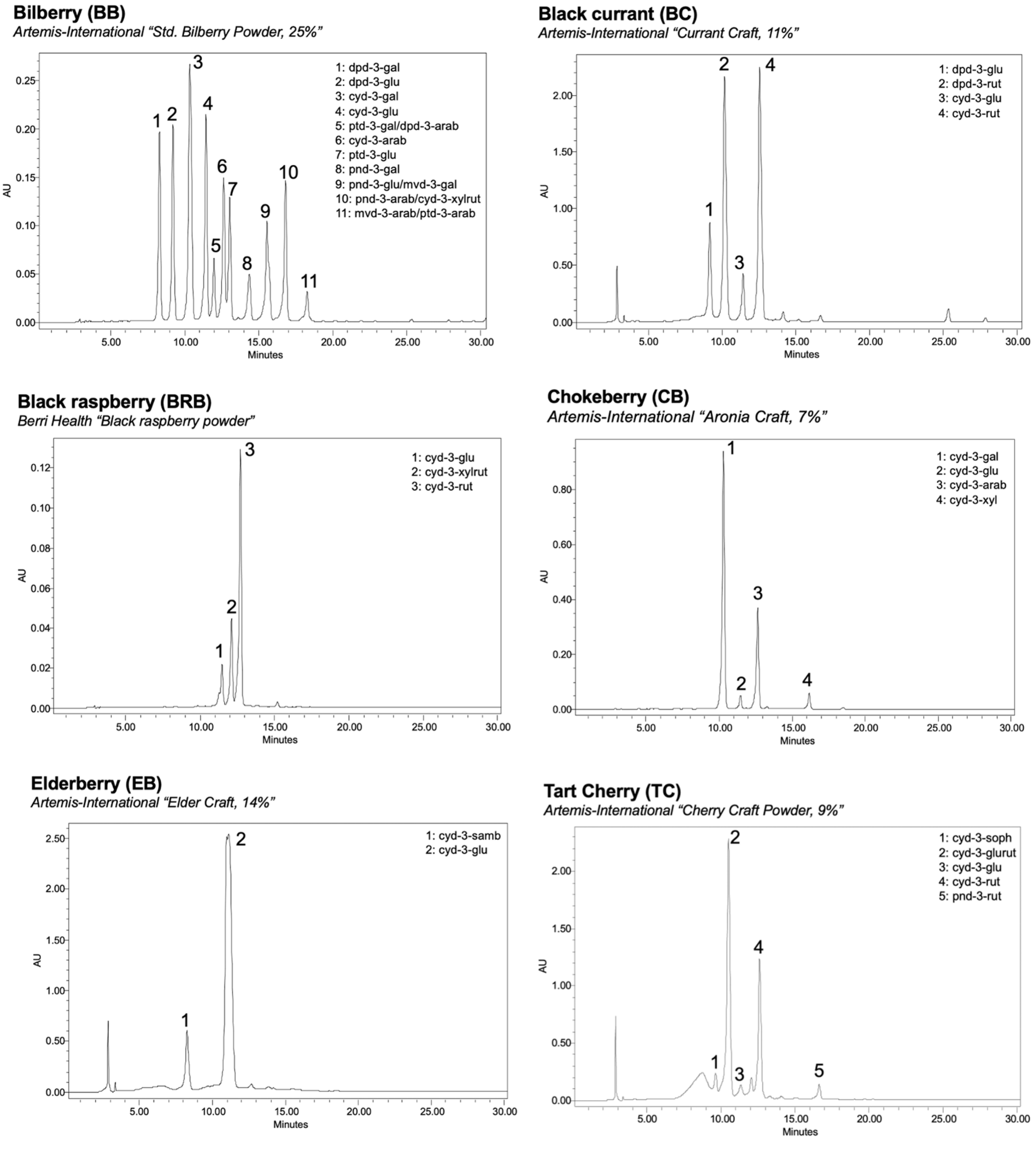

Figure S2. HPLC PDA analysis of anthocyanin-rich berry powders. Chromatograms indicate the identified anthocyanins for each food powder. The relative abundance of the individual chemical components was determined by measuring the area under the curve of the HPLC trace. BB, bilberry; BC, black currant; BRB, black raspberry; CB, chokeberry; EB, elderberry; TC, tart cherry; arab, arabinoside; cyd, cyanidin; dpd, delphinidin; gal, galactoside; glu, glucoside; glurut, glucosyl rutinoside; mvd, malvidin; pnd, pelargonidin; ptd, petunidin; rut, rutinoside; samb, sambubioside; soph, sophoroside; xylrut, xylosyl rutinoside; xyl, xyloside.

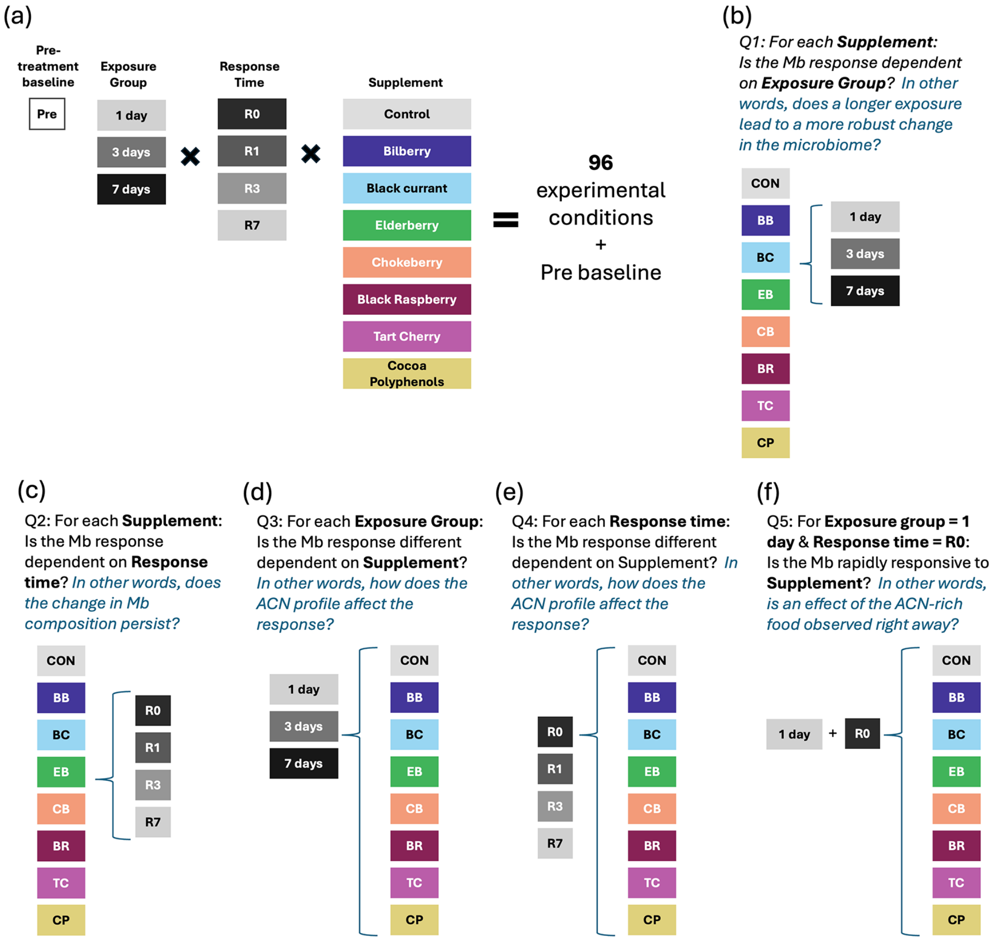

Figure S3. Schematic illustrating stepwise analysis scheme aligned with overarching experimental questions. (**a**) Overview of the experimental setup showing three exposure groups (1, 3, and 7 days) and four response timepoints (R0, R1, R3, and R7) across eight dietary supplements, including six anthocyanin (ACN)-rich foods (bilberry, black currant, elderberry, chokeberry, black raspberry, and tart cherry), one polyphenol-rich food (cocoa powder), and a control group (CON). Fecal samples were also collected prior to dietary intervention (Pre). The full design yielded 96 experimental conditions plus a pre-treatment baseline. (**b–f**) Analytical framework outlining the experimental questions (*Q1* – *Q5*) addressed through stepwise comparisons. Each panel highlights the subset of groups compared to evaluate microbiome (Mb) response patterns with respect to exposure duration (*Q1*), response time (*Q2*), supplement identity (*Q3* and *Q4*), and early responsiveness to intervention (*Q5*). CON, control; BB, bilberry; BC, black currant; EB, elderberry; CB, chokeberry; BRB, black raspberry; TC, tart cherry; CP, cocoa polyphenols.

(a)

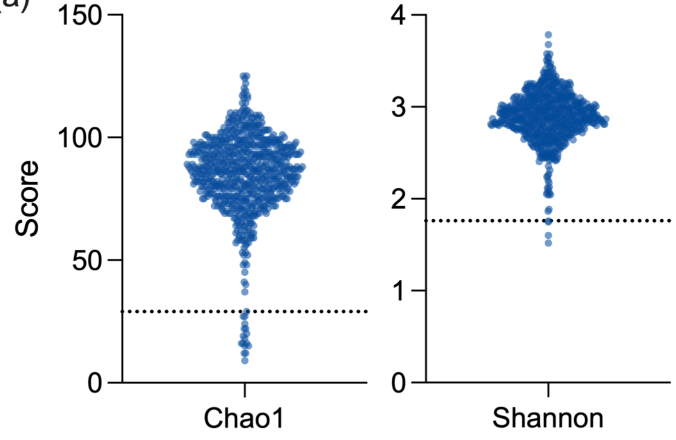

(b)

Excluded samples

ID	Exposure group	Response time	Supplement
Based on alpha diversity outlier analysis			
O23	1 day	R1	BC
O303	3 days	R1	BB
O309	7 days	Pre	CON
O354	3 days	R1	BRB
O399	7 days	R1	BRB
O404	7 days	R7	BC
O411	1 day	R7	EB
O414	7 days	Pre	EB
O415	1 day	R3	BC
O421	3 days	R3	TC
O423	7 days	R7	CB
O437	1 day	R1	CP
O439	1 day	R1	TC
O473	3 days	R3	CP
O475	7 days	R1	CB
O478	7 days	R3	BRB
O479	3 days	Pre	TC
Based on failed squencing			
O340	1 day	R1	BC

(c)

Final sample sizes for microbiome analyses

Exposure group *	Supplement								
Response time	CON	BB	BC	EB	CB	BRB	TC	CP	Total
1 day	30	30	27	29	30	30	29	29	234
Pre	6	6	6	6	6	6	6	6	48
R0	6	6	6	6	6	6	6	6	48
R1	6	6	4	6	6	6	5	5	44
R3	6	6	5	6	6	6	6	6	47
R7	6	6	6	5	6	6	6	6	47
3 days	30	29	30	30	30	29	28	29	235
Pre	6	6	6	6	6	6	6	6	48
R0	6	6	6	6	6	6	5	6	47
R1	6	5	6	6	6	5	6	6	46
R3	6	6	6	6	6	6	5	5	46
R7	6	6	6	6	6	6	6	6	48
7 days	29	30	29	29	28	28	30	30	233
Pre	6	6	6	6	6	6	6	6	48
R0	5	6	6	5	6	6	6	6	46
R1	6	6	6	6	5	5	6	6	46
R3	6	6	6	6	6	5	6	6	47
R7	6	6	5	6	5	6	6	6	46
Total	89	89	86	88	88	87	87	88	702

Figure S4. Outlier analysis of all microbiome samples using alpha diversity. (**a**) Chao1 and Shannon alpha diversity scores for all 719 sequencing samples. (**b**) Table indicating the samples that were excluded following application of the ROUT outlier test using Q=0.01. (**c**) Final sample size for all microbiome analyses. BB, bilberry; BC, black currant; EB, elderberry; CB, chokeberry; BRB, black raspberry; TC, tart cherry.

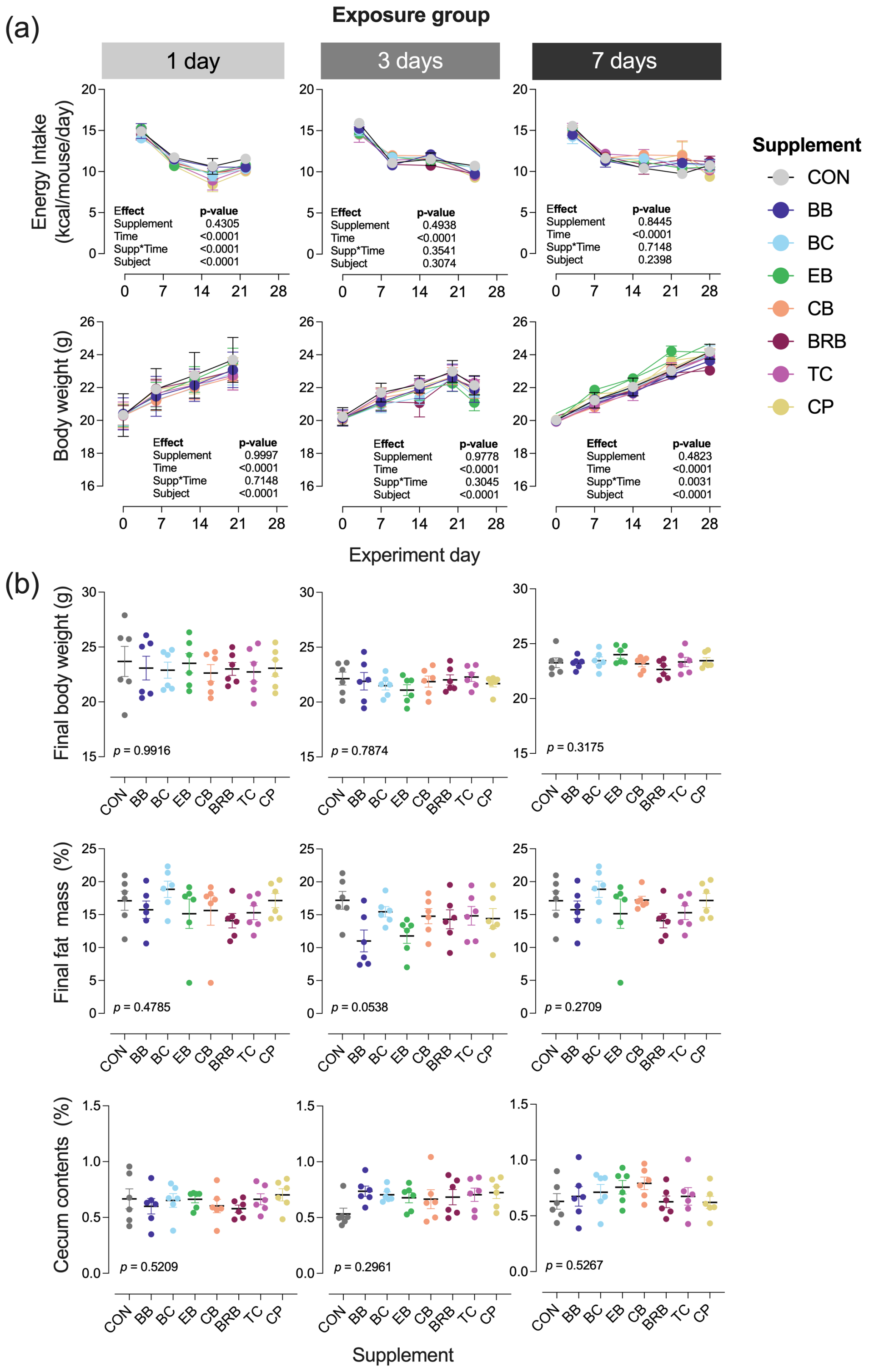

Figure S5. Energy intake, body weight gain, final body weight, body composition, and relative cecal contents weight of mice. (**a**) For each supplement group, estimated daily energy intake and body weight gain within each exposure group. Values shown are the mean ± SEM. (**b**) Body weight, relative fat mass, and relative cecum content mass at the terminal time point for each supplement group within each exposure group. Individual data are shown with the mean ± SEM. CON, control; BB, bilberry; BC, black currant; EB, elderberry; CB, chokeberry; BRB, black raspberry; TC, tart cherry; CP, cocoa polyphenols.

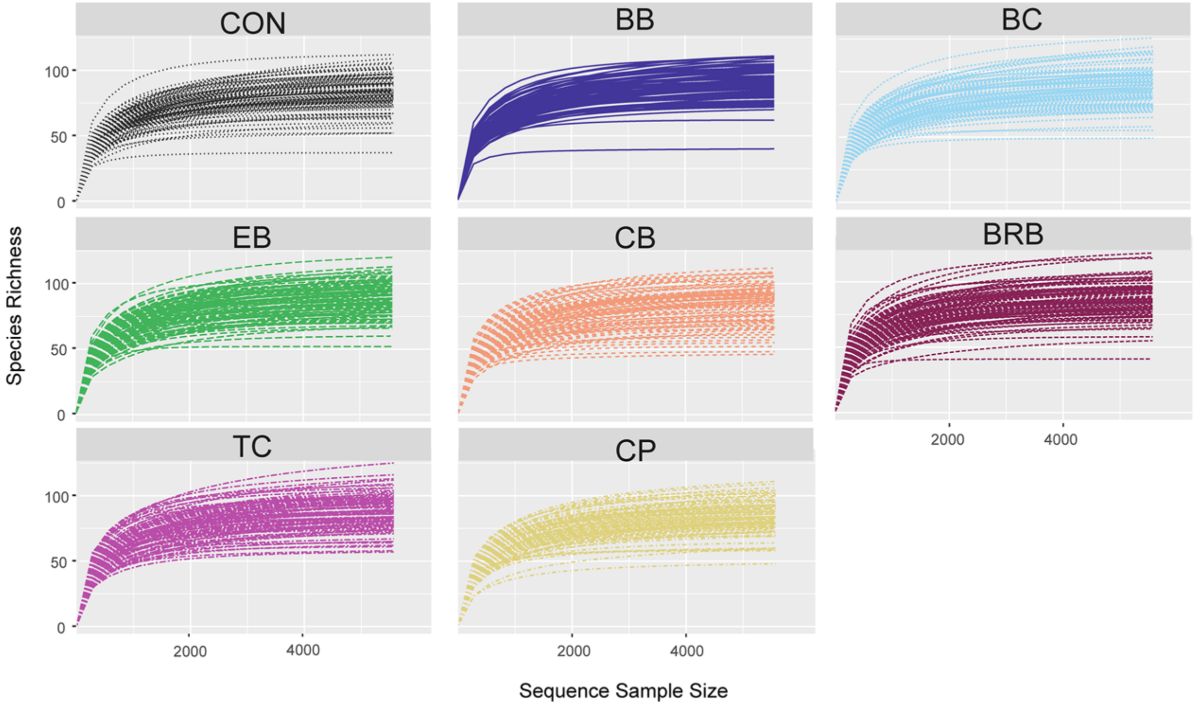

Figure S6. Rarefaction curve analysis by experimental diet. Curves depict species richness as a function of sequencing depth. To ensure consistent comparisons across experimental groups, data were rarefied to approximately 5,500 sequences, corresponding to the lowest sequencing depth among all samples. CON, control; BB, bilberry; BC, black currant; EB, elderberry; CB, chokeberry; BRB, black raspberry; TC, tart cherry; CP, cocoa polyphenols.

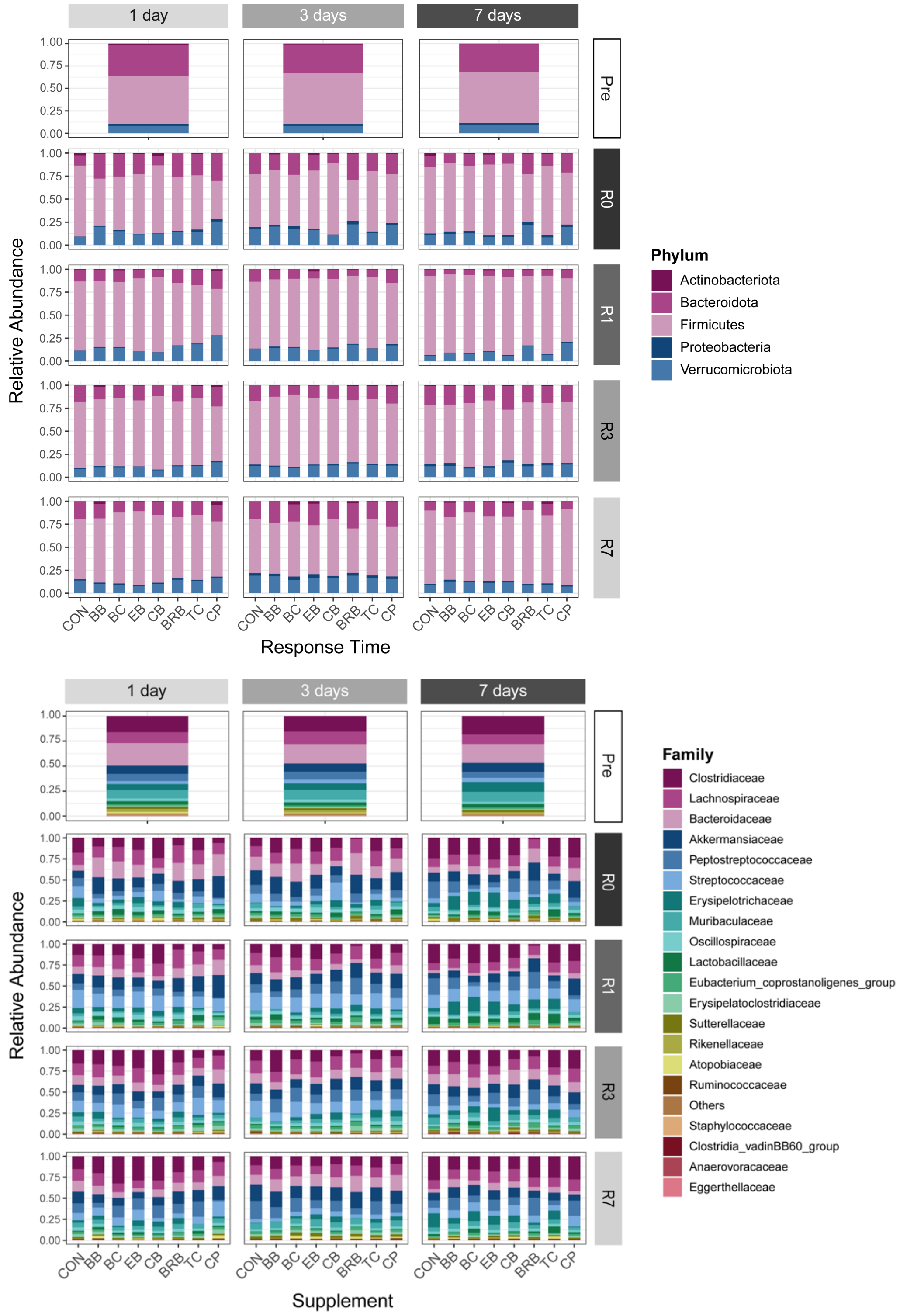

Figure S7. Taxonomic classification of mouse fecal bacteria. Data shown are the relative normalized abundance of bacteria annotated to the phylum (*top*) and family (*bottom*) taxonomic levels for each response time within exposure group for each supplement. At the Pre time point, mice had only been provided the CON diet; thus, only one stacked bar is shown. CON, control; BB, bilberry; BC, black currant; EB, elderberry; CB, chokeberry; BRB, black raspberry; TC, tart cherry; CP, cocoa polyphenols.

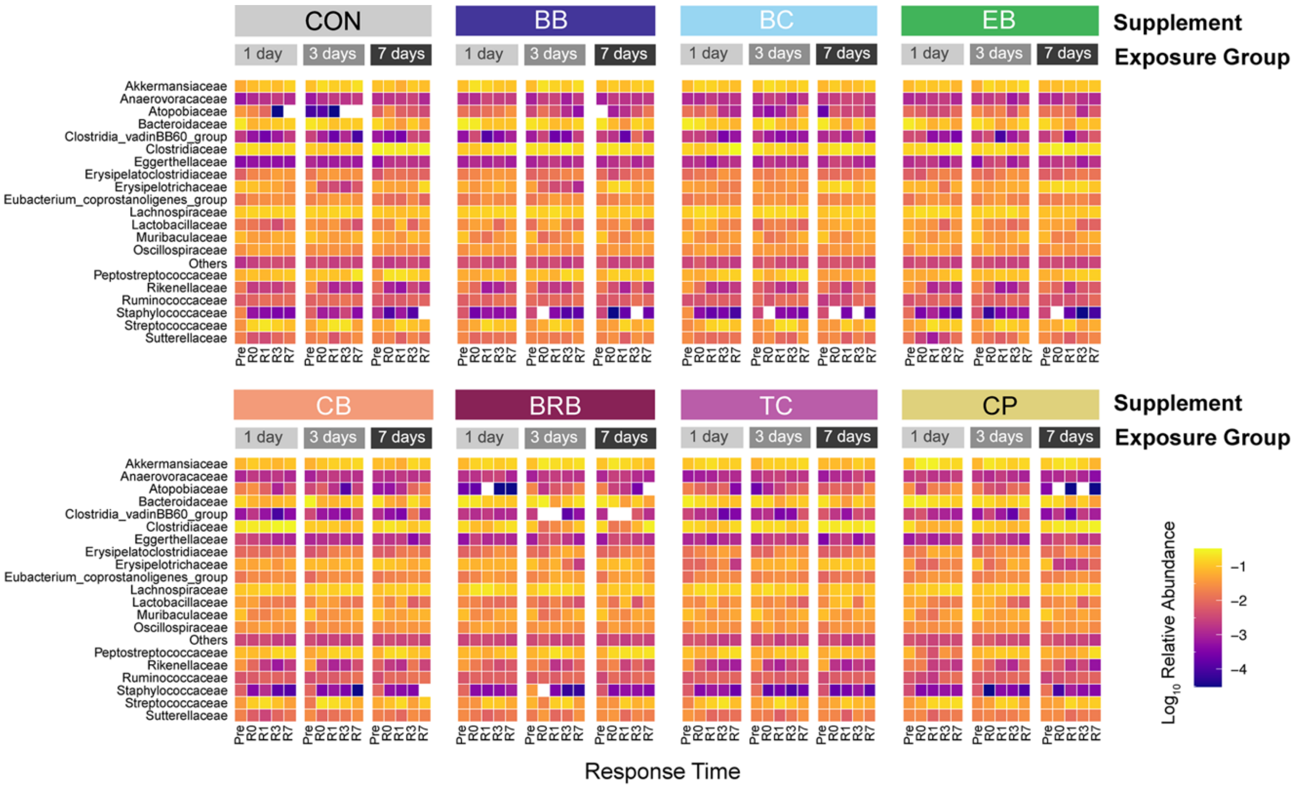

Figure S8. Heatmap of bacterial family-level composition in mouse fecal samples. The color gradient represents the log_{10}-transformed relative abundance, with yellow indicating higher abundance and dark purple indicating lower abundance. Data are grouped by response time, exposure group, and supplement. CON, control; BB, bilberry; BC, black currant; EB, elderberry; CB, chokeberry; BRB, black raspberry; TC, tart cherry; CP, cocoa polyphenols.

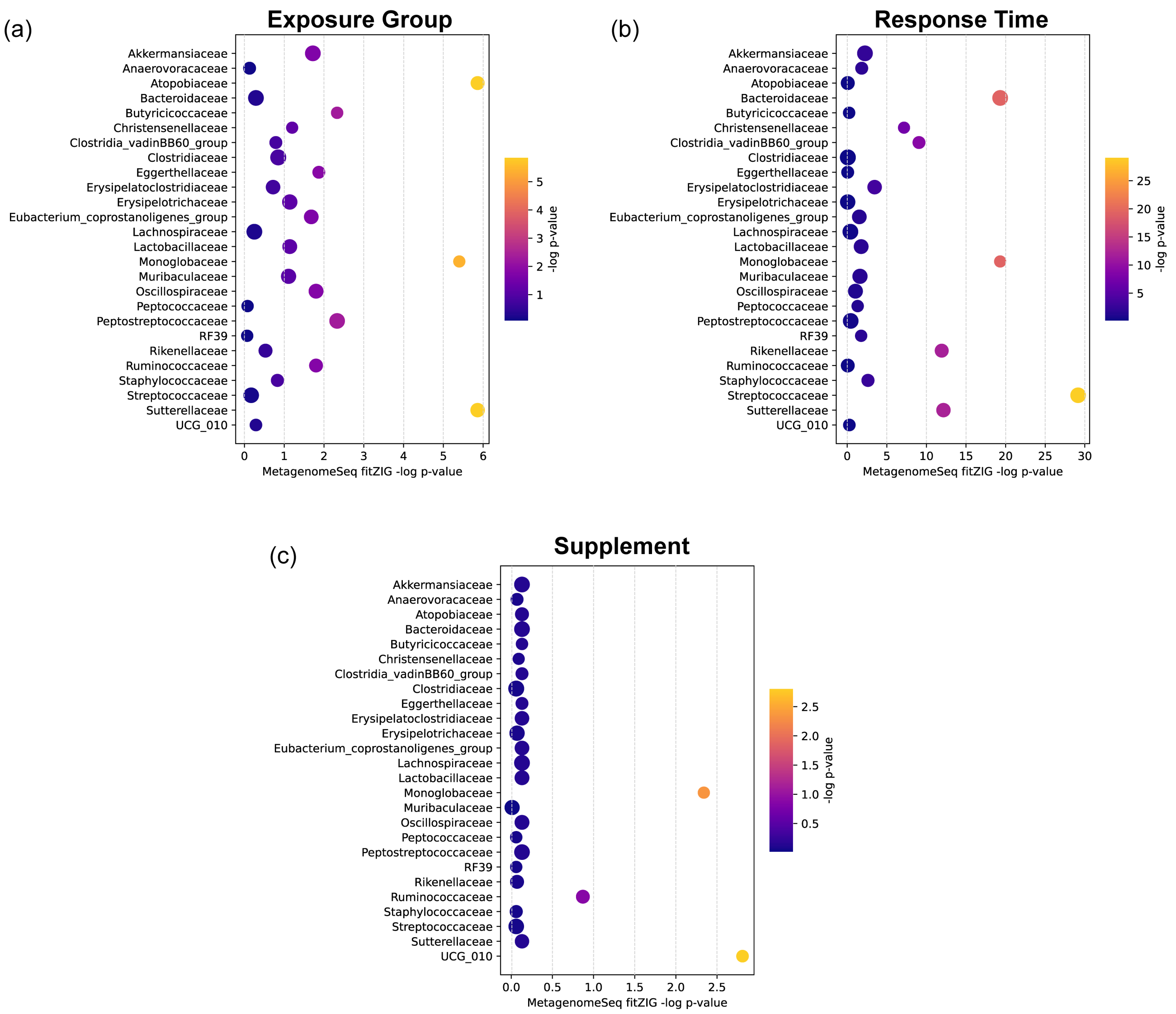

Figure S9. Main effects of exposure group, response time, supplement on relative abundance of bacteria families. Plots show the -log *p*-values following MetagenomeSeq fitZIG analysis for main effects of (**a**) exposure group, (**b**) response time, and (**c**) supplement. Dot color indicates the level of significance and size reflects the relative abundance of the bacteria family in the microbiome population.

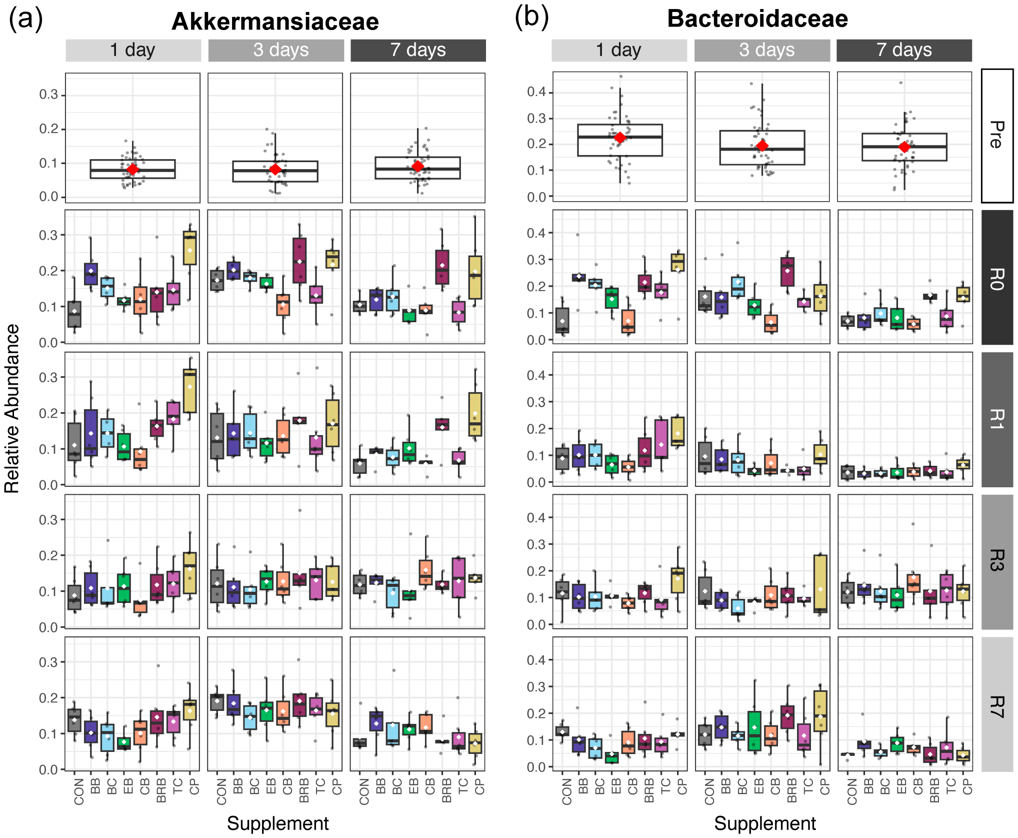

(a) Akkermansiaceae

Supplement within response time

Supplement	R0	R1	R3	R7
CON	ABC	A	A	A
BB	ADE	AB	A	A
BC	ABDE	AB	A	A
EB	ABCD	AB	A	A
CB	C	A	A	A
BRB	DE	BC	A	A
TC	BC	AB	A	A
CP	E	C	A	A

Supplement within exposure group

Supplement	1 day	3 days	7 days
CON	AB	AB	ABC
BB	AC	A	AB
BC	ABC	AB	ABC
EB	ABC	B	C
CB	B	AB	A
BRB	C	AB	BC
TC	CD	AB	ABC
CP	D	AB	ABC

(b) Bacteroidaceae

Supplement within response time

Supplement	R0	R1	R3	R7
CON	AB	AB	A	A
BB	CD	AB	A	A
BC	CD	AB	A	A
EB	AC	A	A	A
CB	B	A	A	A
BRB	D	AB	A	A
TC	AC	AB	A	A
CP	CD	B	A	A

Supplement within exposure group

Supplement	1 day	3 days	7 days
CON	AB	A	A
BB	AC	A	A
BC	AC	A	A
EB	AB	A	A
CB	B	A	A
BRB	AC	A	A
TC	AC	A	A
CP	C	A	A

Figure S10. Relative abundance of Akkermansiaceae and Bacteroidaceae for each exposure group, response time, and supplement. Data are shown as Tukey box plots (box, 25th to 75th percentiles; whiskers, 1.5 IQR; ◇, mean) for relative abundance of (**a**) Akkermansiaceae and (**b**) Bacteroidaceae. Tables below the plots provide the connected letters for statistical analyses of supplement within exposure group (controlling for exposure) and within response time (controlling for exposure). Different letters indicate that the supplement groups are significantly different (FDR $p < 0.05$). Complete statistical results are available in File 2. CON, control; BB, bilberry; BC, black currant; EB, elderberry; CB, chokeberry; BRB, black raspberry; TC, tart cherry; CP, cocoa polyphenols.

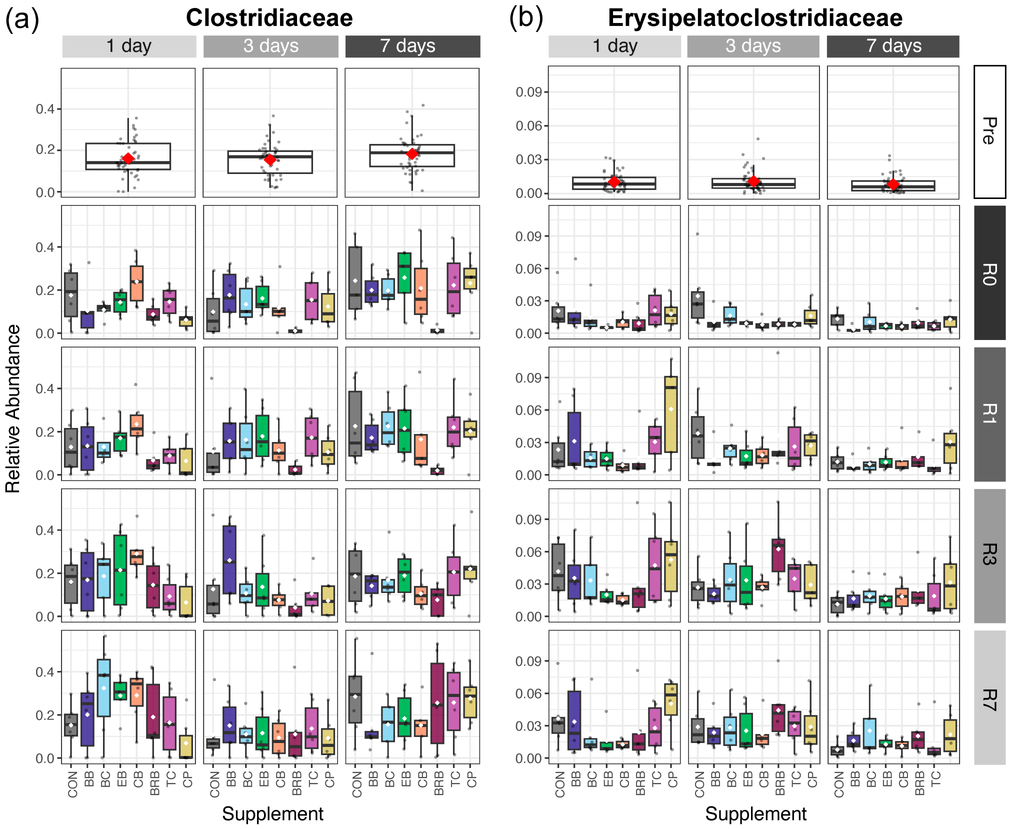

(a)

Supplement within response time

Supplement	R0	R1	R3	R7
CON	A	AB	A	A
BB	A	A	A	A
BC	A	A	A	A
EB	A	A	A	A
CB	A	A	A	A
BRB	B	B	A	A
TC	A	A	A	A
CP	A	AB	A	A

Supplement within exposure group

Supplement	1 day	3 days	7 days
CON	AB	ABC	ABCD
BB	AC	A	AB
BC	ABD	DE	EF
EB	BD	ABD	ACE
CB	D	B	CE
BRB	AB	CE	DF
TC	AB	ACDE	BDF
CP	C	ABCDE	ABCDEF

(b)

Supplement within response time

Supplement	R0	R1	R3	R7
CON	A	AB	A	A
BB	B	A	A	A
BC	AB	AB	A	A
EB	B	AB	A	A
CB	B	AB	A	A
BRB	B	AB	A	A
TC	AB	AB	A	A
CP	AB	B	A	A

Supplement within exposure group

Supplement	1 day	3 days	7 days
CON	ABC	ABC	A
BB	AB	AB	A
BC	AC	AC	A
EB	C	C	A
CB	C	C	A
BRB	C	C	A
TC	AB	AB	A
CP	B	B	A

Figure S11. Relative abundance of Clostridiaceae and Erysipelatoclostridiaceae for each exposure group, response time, and supplement. Data are shown as Tukey box plots (box, 25th to 75th percentiles; whiskers, 1.5 IQR; ◇, mean) for relative abundance of (**a**) Clostridiaceae and (**b**) Erysipelatoclostridiaceae. Tables below the plots provide the connected letters for statistical analyses of supplement within exposure group (controlling for response time) and within response time (controlling for exposure). Different letters indicate that the supplement groups are significantly different (FDR $p < 0.05$). Complete statistical results are available in File 2. CON, control; BB, bilberry; BC, black currant; EB, elderberry; CB, chokeberry; BRB, black raspberry; TC, tart cherry; CP, cocoa polyphenols.

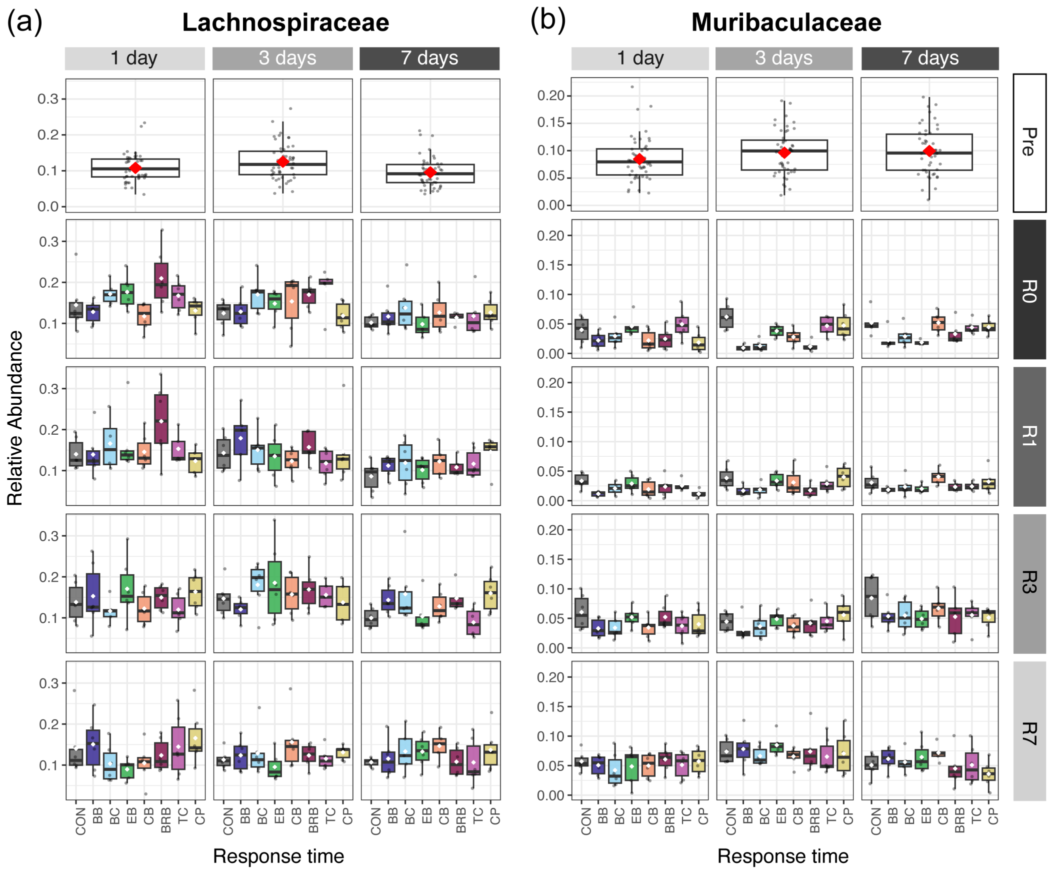

(a) Lachnospiraceae

Supplement within response time

Supplement	R0	R1	R3	R7
CON	A	A	A	A
BB	AB	A	A	A
BC	BC	A	A	A
EB	ABC	A	A	A
CB	AB	A	A	A
BRB	C	A	A	A
TC	ABC	A	A	A
CP	AB	A	A	A

Supplement within exposure group

Supplement	1 day	3 days	7 days
CON	AB	A	AB
BB	AB	A	AC
BC	AB	A	ABCD
EB	AB	A	CD
CB	A	A	CD
BRB	B	A	ABCD
TC	AB	A	BD
CP	AB	A	ABCD

(b) Muribaculaceae

Supplement within response time

Supplement	R0	R1	R3	R7
CON	A	A	A	A
BB	B	B	A	A
BC	BCD	AB	A	A
EB	ACD	AB	A	A
CB	ABCD	AB	A	A
BRB	BD	AB	A	A
TC	A	AB	A	A
CP	ACD	AB	A	A

Supplement within exposure group

Supplement	1 day	3 days	7 days
CON	A	A	AB
BB	B	B	AC
BC	AB	CD	C
EB	AB	ABC	ABCD
CB	B	BE	ABCD
BRB	AB	D	BD
TC	AB	DE	CD
CP	B	CD	ABCD

Figure S12. Relative abundance of Lachnospiraceae and Muribaculaceae for each exposure group, response time, and supplement. Data are shown as Tukey box plots (box, 25th to 75th percentiles; whiskers, 1.5 IQR; ◇, mean) for relative abundance of (**a**) Lachnospiraceae and (**b**) Muribaculaceae. Tables below the plots provide the connected letters for statistical analyses of supplement within response time (controlling for exposure) and within exposure group (controlling for response time). Different letters indicate that the supplement groups are significantly different (FDR $p < 0.05$). Complete statistical results are available in File 2. CON, control; BB, bilberry; BC, black currant; EB, elderberry; CB, chokeberry; BRB, black raspberry; TC, tart cherry; CP, cocoa polyphenols.

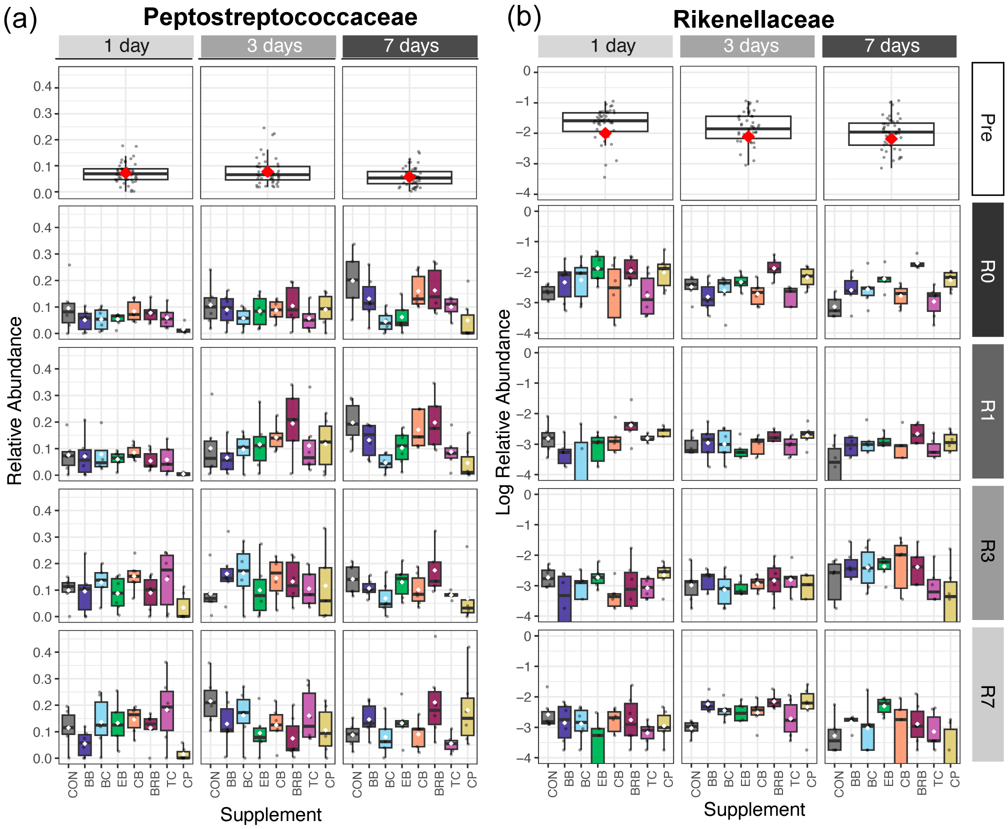

(a) Supplement within response time

Supplement	R0	R1	R3	R7
CON	AB	A	AB	A
BB	AB	A	AB	A
BC	AC	AB	AB	A
EB	ABC	A	AB	A
CB	B	A	A	A
BRB	AB	A	A	A
TC	AB	A	AB	A
CP	C	B	B	A

(a) Supplement within exposure group

Supplement	1 day	3 days	7 days
CON	AB	A	AB
BB	A	A	ACD
BC	AB	A	BEF
EB	AB	A	ABCEG
CB	B	A	DF
BRB	AB	A	CDEF
TC	AB	A	ABCEG
CP	C	A	G

(b) Supplement within response time

Supplement	R0	R1	R3	R7
CON	A	AB	A	A
BB	AB	A	A	A
BC	AB	A	A	A
EB	BC	A	A	A
CB	A	A	A	A
BRB	C	B	A	A
TC	A	A	A	A
CP	BC	AB	A	A

(b) Supplement within exposure group

Supplement	1 day	3 days	7 days
CON	AB	A	ABCDE
BB	A	A	AB
BC	AB	A	CD
EB	AB	A	ACD
CB	AB	A	ABC
BRB	B	A	DE
TC	AB	A	BE
CP	AB	A	ABCDE

Figure S13. Relative abundance of Peptostreptococcaceae and Rikenellaceae for each exposure group, response time, and supplement. Data are shown as Tukey box plots (box, 25th to 75th percentiles; whiskers, 1.5 IQR; ◇, mean) for relative abundance of (**a**) Peptostreptococcaceae and (**b**) Rikenellaceae. Tables below the plots provide the connected letters for statistical analyses of supplement within response time (controlling for exposure) and within exposure group (controlling for response time). Different letters indicate that the supplement groups are significantly different (FDR $p < 0.05$). Complete statistical results are available in File 2. CON, control; BB, bilberry; BC, black currant; EB, elderberry; CB, chokeberry; BRB, black raspberry; TC, tart cherry; CP, cocoa polyphenols.

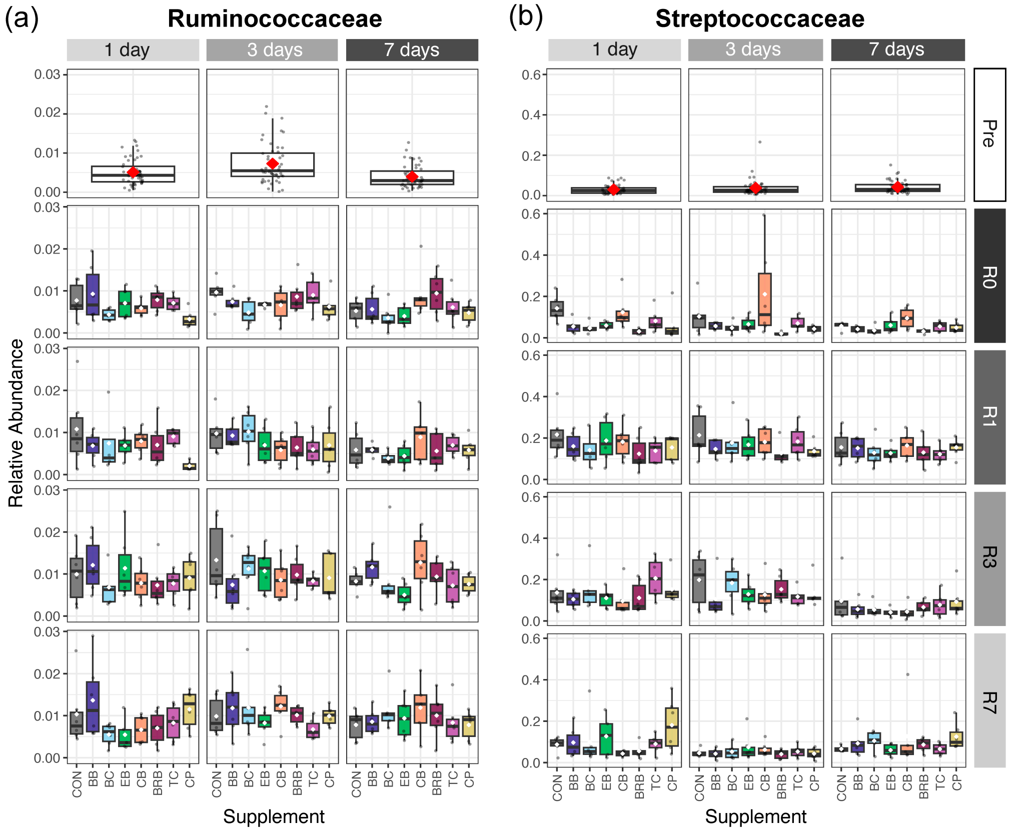

Supplement within response time (a)

Supplement	R0	R1	R3	R7
CON	ABC	A	A	A
BB	A	A	A	A
BC	B	A	A	A
EB	AC	A	A	A
CB	AC	A	A	A
BRB	D	A	A	A
TC	BC	A	A	A
CP	A	A	A	A

Supplement within exposure group (a)

Supplement	1 day	3 days	7 days
CON	A	A	A
BB	A	A	A
BC	AB	A	A
EB	AB	A	A
CB	AB	A	A
BRB	A	A	A
TC	A	A	A
CP	B	A	A

Supplement within response time (b)

Supplement	R0	R1	R3	R7
CON	AB	A	A	A
BB	C	AB	A	A
BC	CD	AB	A	A
EB	AC	AB	A	A
CB	B	AB	A	A
BRB	D	B	A	A
TC	AC	AB	A	A
CP	CD	AB	A	A

Supplement within exposure group (b)

Supplement	1 day	3 days	7 days
CON	A	AB	AB
BB	AB	A	A
BC	AB	AB	AB
EB	AB	B	B
CB	AB	AB	AB
BRB	B	AB	AB
TC	AB	AB	AB
CP	A	AB	AB

Figure S14. Relative abundance of Ruminococcaceae and Streptococcaceae for each exposure group, response time, and supplement. Data are shown as Tukey box plots (box, 25th to 75th percentiles; whiskers, 1.5 IQR; ◇, mean) for relative abundance of (**a**) Ruminococcaceae and (**b**) Streptococcaceae. Tables below the plots provide the connected letters for statistical analyses of supplement within response time (controlling for exposure) and within exposure group (controlling for response time). Different letters indicate that the supplement groups are significantly different (FDR $p < 0.05$). Complete statistical results are available in File 2. CON, control; BB, bilberry; BC, black currant; EB, elderberry; CB, chokeberry; BRB, black raspberry; TC, tart cherry; CP, cocoa polyphenols.

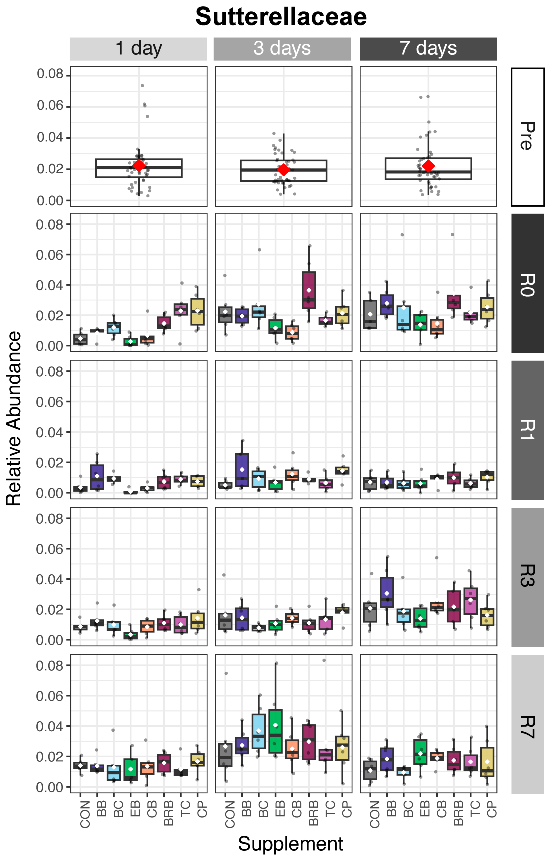

Supplement within response time

Supplement	R0	R1	R3	R7
CON	AB	A	AB	A
BB	AC	A	A	A
BC	AC	A	AB	A
EB	D	B	B	A
CB	BD	A	A	A
BRB	C	A	A	A
TC	AC	A	A	A
CP	AC	A	A	A

Supplement within exposure group

Supplement	1 day	3 days	7 days
CON	AB	AB	AB
BB	AC	A	A
BC	AC	AB	AB
EB	D	B	B
CB	B	AB	AB
BRB	C	AB	AB
TC	C	AB	AB
CP	C	AB	AB

Figure S15. Relative abundance of Sutterellaceae for each exposure group, response time, and supplement. Data are shown as Tukey box plots (box, 25th to 75th percentiles; whiskers, 1.5 IQR; ◇, mean) for relative abundance of Sutterellaceae. Tables below the plots provide the connected letters for statistical analyses of supplement within response time (controlling for exposure) and within exposure group (controlling for response time). Different letters indicate that the supplement groups are significantly different (FDR *p*< 0.05). Complete statistical results are available in File 2. CON, control; BB, bilberry; BC, black currant; EB, elderberry; CB, chokeberry; BRB, black raspberry; TC, tart cherry; CP, cocoa polyphenols.

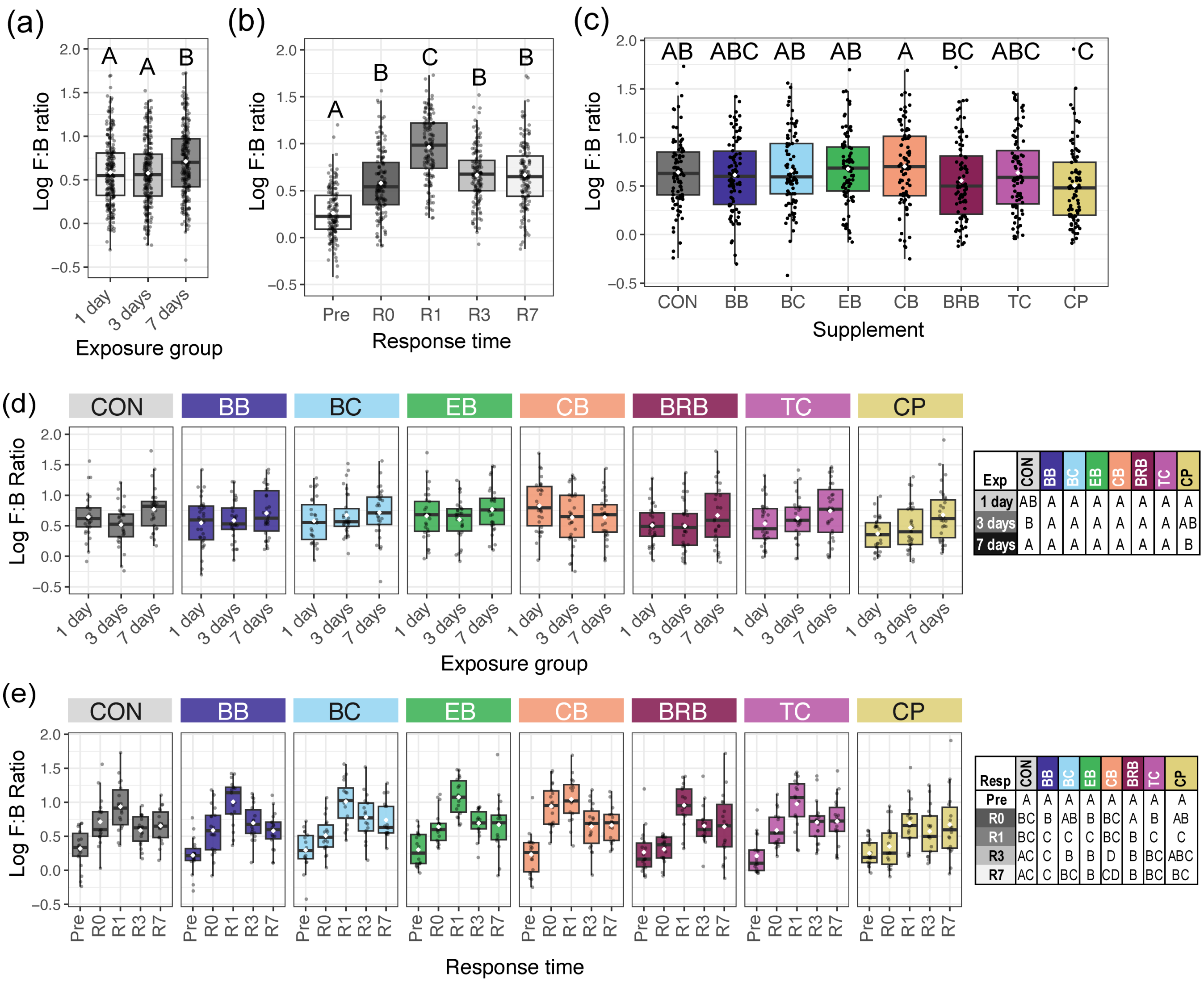

Exp	CON	BB	BC	EB	CB	BRB	TC	CP
1 day	AB	A	A	A	A	A	A	A
3 days	B	A	A	A	A	A	A	AB
7 days	A	A	A	A	A	A	A	B

Resp	CON	BB	BC	EB	CB	BRB	TC	CP
Pre	A	A	A	A	A	A	A	A
R0	BC	B	AB	B	BC	A	B	AB
R1	BC	C	C	C	BC	B	C	C
R3	AC	C	B	B	D	B	BC	ABC
R7	AC	C	BC	B	CD	B	BC	BC

Figure S16. Firmicutes-to-Bacteroidetes (F:B) ratio for exposure group, response time, and supplement. F:B ratio data are shown as Tukey box plots (box, 25th to 75th percentiles; whiskers, 1.5 IQR; ◇, mean) for (**a**) main effect of exposure group, (**b**) main effect of response time, (**c**) main effect of supplement, (**d**) effect of exposure groups within each supplement, and (**e**) effect of response times within each supplement. (**d-e**) Tables provide the connected letters for statistical analyses of supplement within exposure group (controlling for exposure) and within response time (controlling for exposure). For all panels, different letters indicate that the supplement groups are significantly different (FDR $p < 0.05$). Complete statistical results are provided in Tables S3-S8. CON, control; BB, bilberry; BC, black currant; EB, elderberry; CB, chokeberry; BRB, black raspberry; TC, tart cherry; CP, cocoa polyphenols.

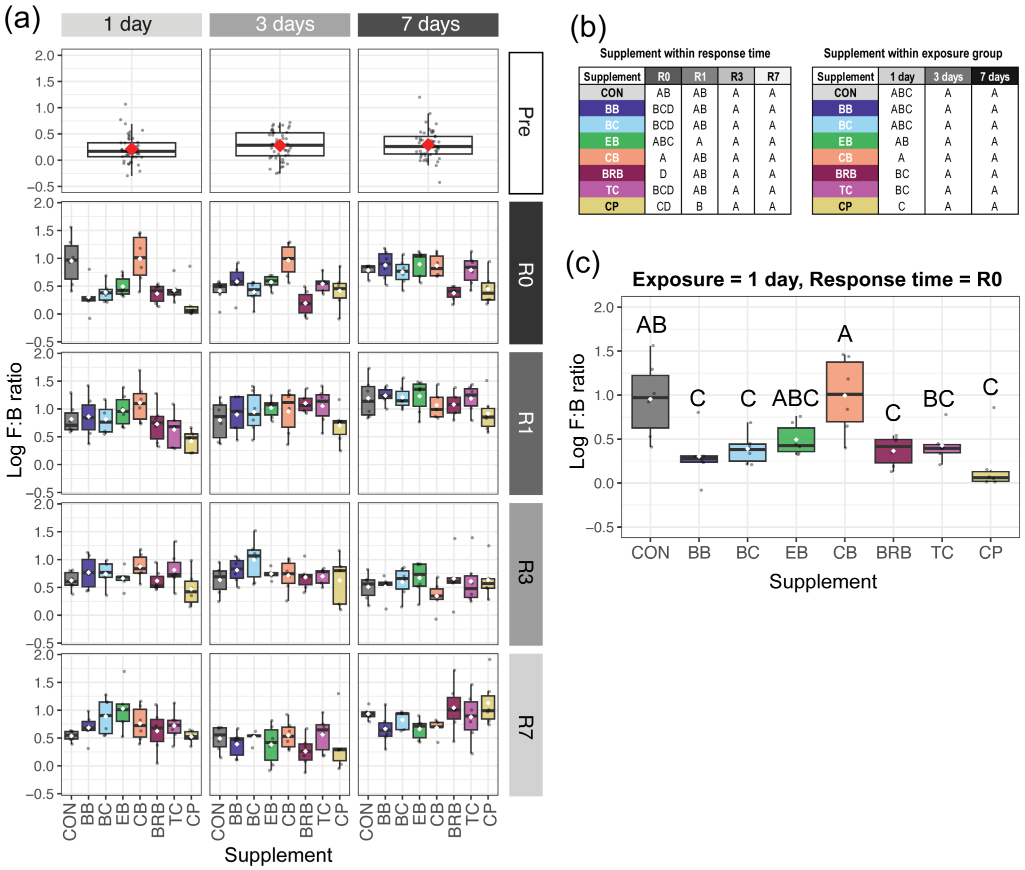

Supplement within response time

Supplement	R0	R1	R3	R7
CON	AB	AB	A	A
BB	BCD	AB	A	A
BC	BCD	AB	A	A
EB	ABC	A	A	A
CB	A	AB	A	A
BRB	D	AB	A	A
TC	BCD	AB	A	A
CP	CD	B	A	A

Supplement within exposure group

Supplement	1 day	3 days	7 days
CON	ABC	A	A
BB	ABC	A	A
BC	ABC	A	A
EB	AB	A	A
CB	A	A	A
BRB	BC	A	A
TC	BC	A	A
CP	C	A	A

Figure S17. Firmicutes-to-Bacteroidetes (F:B) ratio for supplement within exposure group and response time. (**a**) F:B ratio data are shown as Tukey box plots (box, 25th to 75th percentiles; whiskers, 1.5 IQR; ◇, mean). (**b**) Table provides the connected letters for statistical analyses of supplement within exposure group (controlling for exposure) and within response time (controlling for exposure). (**c**) F:B ratio shown as Tukey plots for supplement with exposure group = 1 day and response time = R0. For all panels, different letters indicate that the supplement groups are significantly different (FDR $p<0.05$). Complete statistical results are provided in Tables S9-S11. CON, control; BB, bilberry; BC, black currant; EB, elderberry; CB, chokeberry; BRB, black raspberry; TC, tart cherry; CP, cocoa polyphenols.

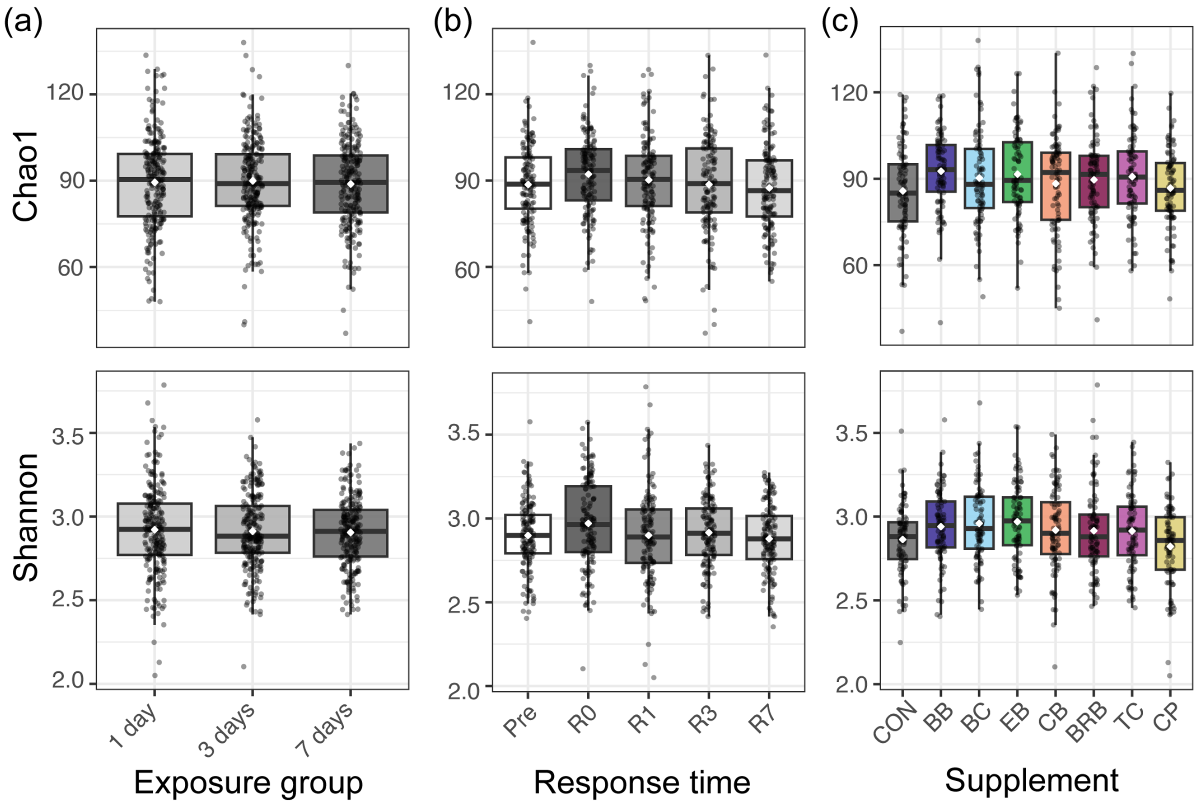

Exposure	Chao1	Shannon
1 day	A	A
3 days	A	A
7 days	A	A

Response	Chao1	Shannon
Pre	A	A
R0	A	B
R1	A	AB
R3	A	AB
R7	A	A

Supplement	Chao1	Shannon
CON	A	AC
BB	B	A
BC	AB	A
EB	AB	B
CB	AB	AC
BRB	AB	AC
TC	AB	AC
CP	AB	C

Figure S18. Alpha diversity for exposure group, response time, and supplement. Chao1 and Shannon alpha diversity score data are shown as Tukey box plots (box, 25th to 75th percentiles; whiskers, 1.5 IQR; ◇, mean) for (**a**) main effect of exposure group, (**b**) main effect of response time, and (**c**) main effect of supplement. Tables provide the connected letters for pairwise comparisons of levels within each factor, and different letters indicate that the levels are significantly different (FDR $p < 0.05$). Complete statistical results are provided in Tables S12-S15. CON, control; BB, bilberry; BC, black currant; EB, elderberry; CB, chokeberry; BRB, black raspberry; TC, tart cherry; CP, cocoa polyphenols.

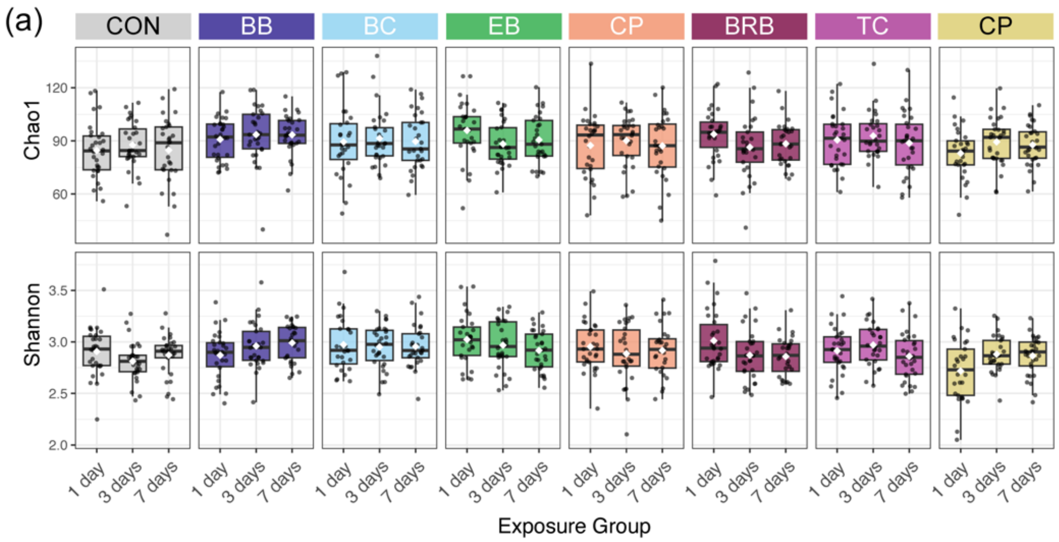

Exp	CON	BB	BC	EB	CB	BRB	TC	CP
1 day	A	A	A	A	A	A	A	A
3 days	A	A	A	A	A	A	A	A
7 days	A	A	A	A	A	A	A	A

Exp	CON	BB	BC	EB	CB	BRB	TC	CP
1 day	A	A	A	A	A	A	A	A
3 days	A	A	A	A	A	A	A	B
7 days	A	A	A	A	A	A	A	B

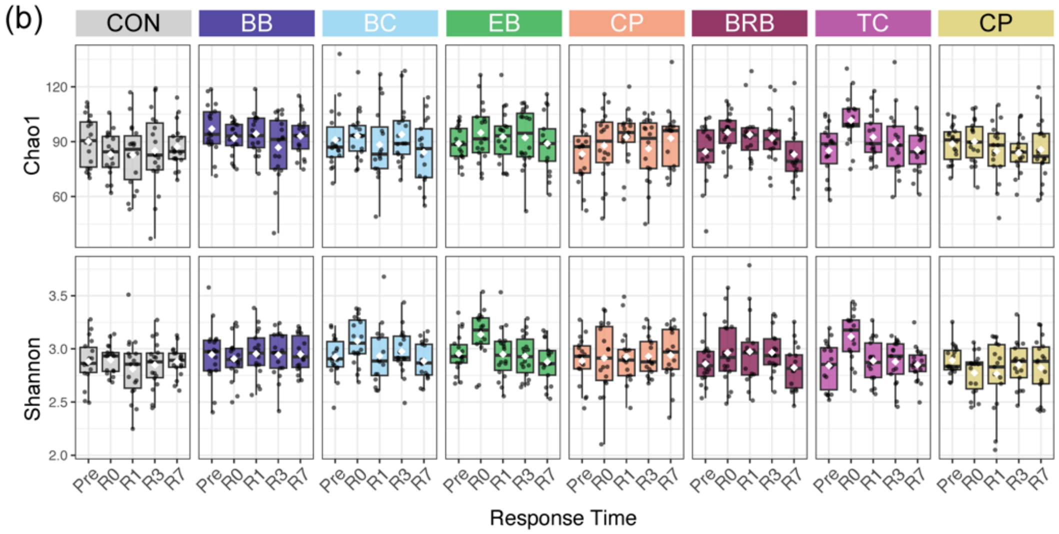

Resp	CON	BB	BC	EB	CB	BRB	TC	CP
Pre	A	A	A	A	A	A	A	A
R0	A	A	A	A	A	A	B	A
R1	A	A	A	A	A	A	AB	A
R3	A	A	A	A	A	A	AB	A
R7	A	A	A	A	A	A	A	A

Resp	CON	BB	BC	EB	CB	BRB	TC	CP
Pre	A	A	A	AB	A	A	A	A
R0	A	A	A	A	A	A	B	A
R1	A	A	A	AB	A	A	A	A
R3	A	A	A	B	A	A	A	A
R7	A	A	A	B	A	A	A	A

Figure S19. Alpha diversity for exposure group or response time within each supplement group. Chao1 and Shannon alpha diversity score data are shown as Tukey box plots (box, 25th to 75th percentiles; whiskers, 1.5 IQR; ◇, mean) for (**a**) effect of exposure group within each supplement or (**b**) effect of response time within each supplement. Tables provide the connected letters for pairwise comparisons of levels within each supplement, and different letters indicate that the levels are significantly different (FDR $p < 0.05$). Complete statistical results are provided in Tables S16-S17. CON, control; BB, bilberry; BC, black currant; EB, elderberry; CB, chokeberry; BRB, black raspberry; TC, tart cherry; CP, cocoa polyphenols.

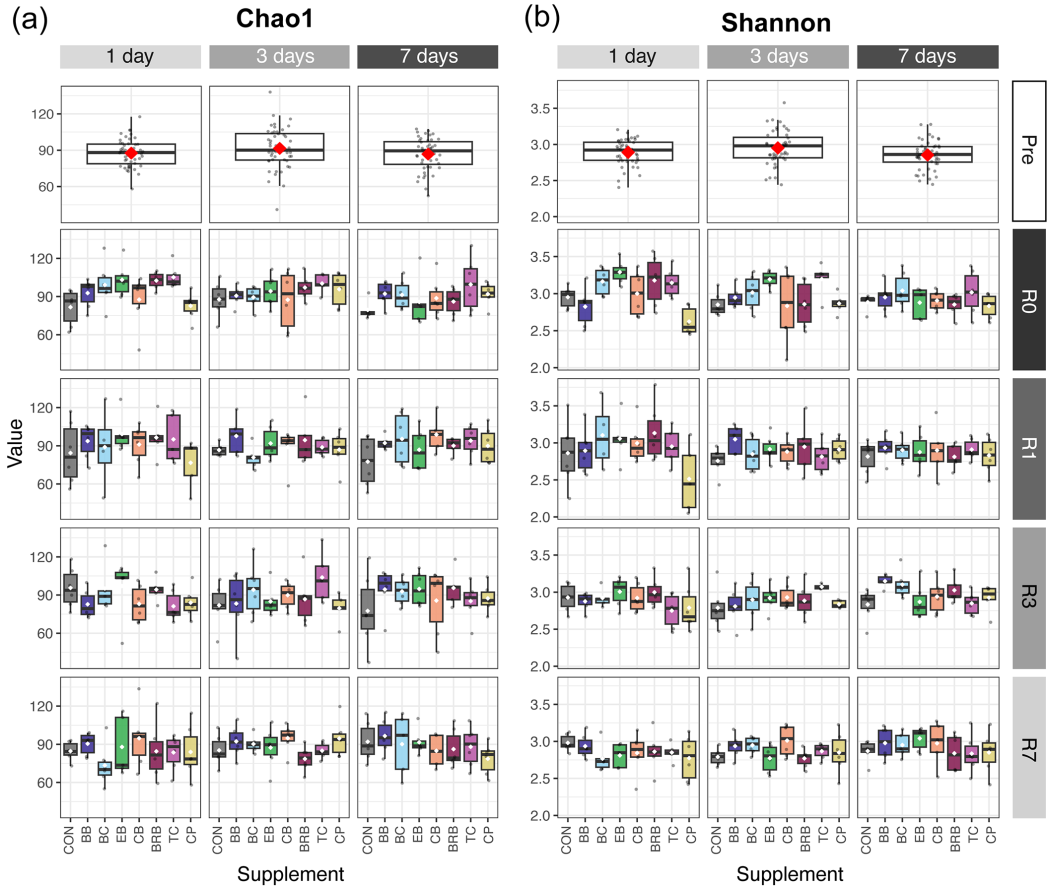

(a) Chao1

Supplement within response time

Supplement	R0	R1	R3	R7
CON	A	A	A	A
BB	AB	A	A	A
BC	AB	A	A	A
EB	AB	A	A	A
CB	AB	A	A	A
BRB	AB	A	A	A
TC	B	A	A	A
CP	AB	A	A	A

Supplement within exposure group

Supplement	1 day	3 days	7 days
CON	AB	A	A
BB	AB	A	A
BC	AB	A	A
EB	A	A	A
CB	AB	A	A
BRB	AB	A	A
TC	AB	A	A
CP	B	A	A

(b) Shannon

Supplement within response time

Supplement	R0	R1	R3	R7
CON	AB	A	A	A
BB	AB	A	A	A
BC	A	A	A	A
EB	A	A	A	A
CB	AB	A	A	A
BRB	AB	A	A	A
TC	A	A	A	A
CP	B	A	A	A

Supplement within exposure group

Supplement	1 day	3 days	7 days
CON	AB	A	A
BB	AB	A	A
BC	A	A	A
EB	A	A	A
CB	A	A	A
BRB	A	A	A
TC	AB	A	A
CP	B	A	A

Figure S20. Alpha diversity for supplement within exposure group and response time. (**a**) Chao1 and (**b**) Shannon alpha diversity score data are shown as Tukey box plots (box, 25th to 75th percentiles; whiskers, 1.5 IQR; ◇, mean). Tables provide the connected letters for statistical analyses of supplement within exposure group (controlling for exposure) and within response time (controlling for exposure). Different letters indicate that the supplement groups are significantly different (FDR $p < 0.05$). Complete statistical results are provided in Tables S18-S19. CON, control; BB, bilberry; BC, black currant; EB, elderberry; CB, chokeberry; BRB, black raspberry; TC, tart cherry; CP, cocoa polyphenols.

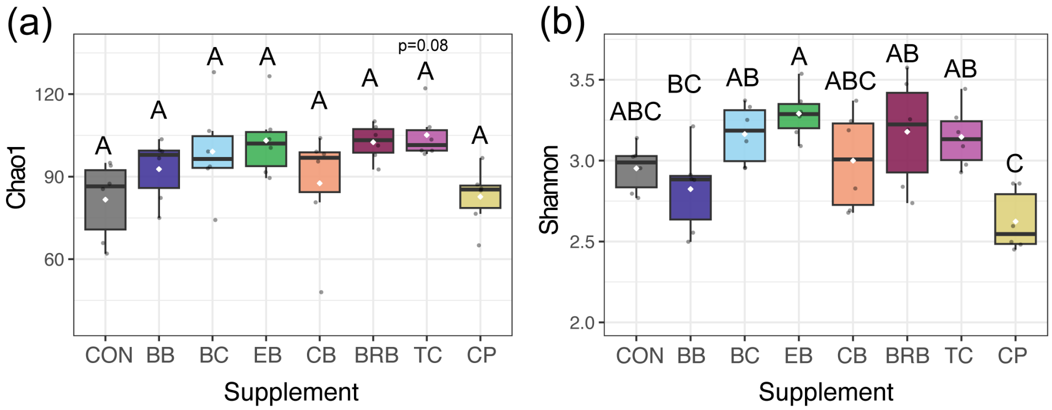

Figure S21. Alpha diversity for supplement with exposure group = 1 day and response time = R0. (**a**) Chao1 and (**b**) Shannon alpha diversity score data are shown as Tukey box plots (box, 25th to 75th percentiles; whiskers, 1.5 IQR; ◇, mean). Different letters indicate that the supplement groups are significantly different (FDR p<0.05). A trend for significant effect of TC compared to CON for Chao1 is noted (p = 0.08). Complete statistical results are provided in Tables S20. CON, control; BB, bilberry; BC, black currant; EB, elderberry; CB, chokeberry; BRB, black raspberry; TC, tart cherry; CP, cocoa polyphenols.

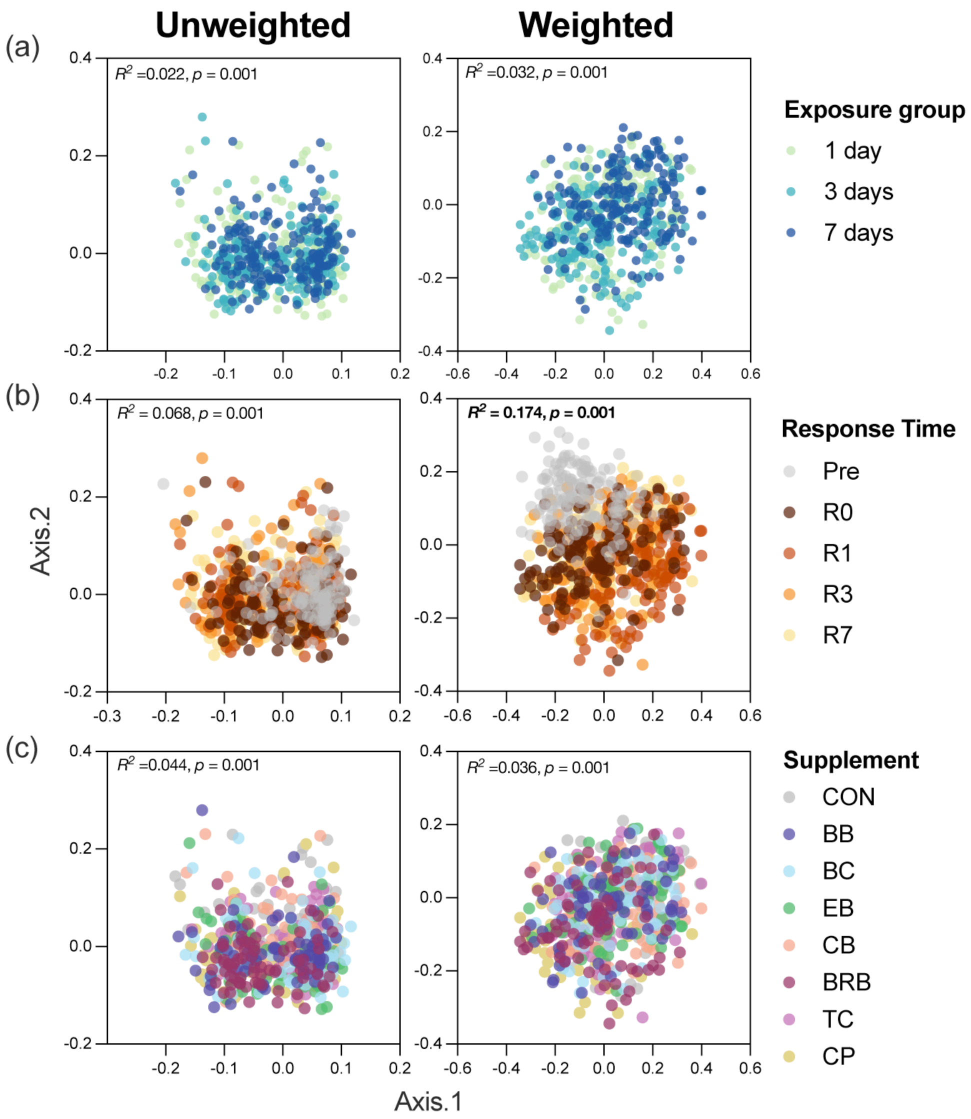

Figure S22. UniFrac beta diversity of mouse fecal microbiomes for exposure group, response time, or supplement. Principal coordinate plots show the unweighted or weighted UniFrac distances for (**a**) exposure group, (**b**) response time, (**c**) or supplement using the first two coordinates. PERMANOVA R^2 and p-values shown. FDR adjusted $p < 0.05$ was considered statistically significant, with a meaningful effect size set at $R^2 > 0.1$. PEMANOVA pairwise comparison results are provided in Tables 2.21-23.

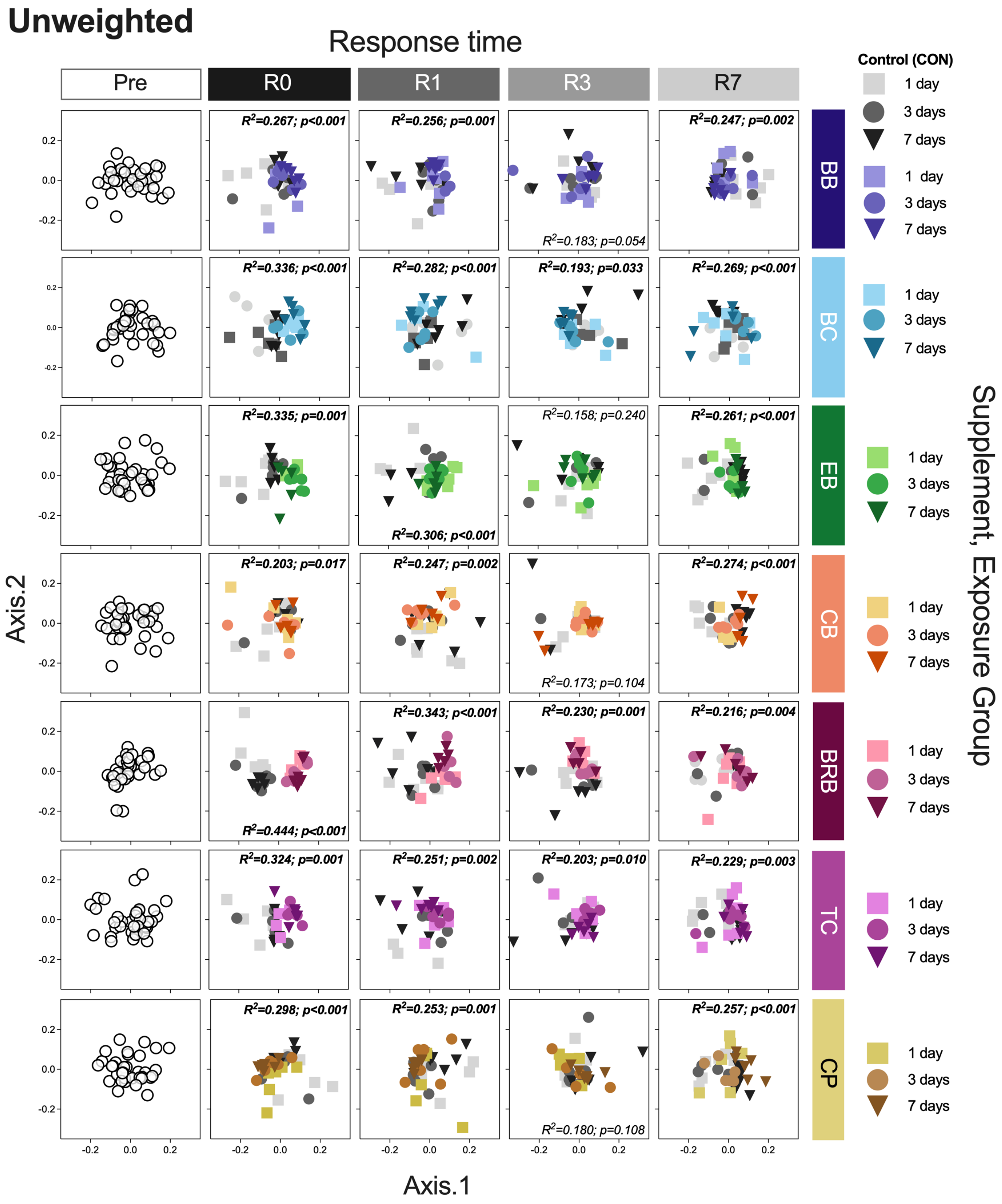

Figure S23. Unweighted UniFrac beta diversity of mouse fecal microbiomes for exposure group, response time, or supplement. Principal coordinate plots show the unweighted UniFrac distances for CON vs each supplement with data stratified by exposure group (■, 1 day; ●, 3 days; ▼, 7 days) or response time (in columns). PERMANOVA R^2 and p-values shown. FDR adjusted $p < 0.05$ was considered statistically significant, with a meaningful effect size set at $R^2 > 0.1$. PEMANOVA pairwise comparison results are provided in Table 2.24.

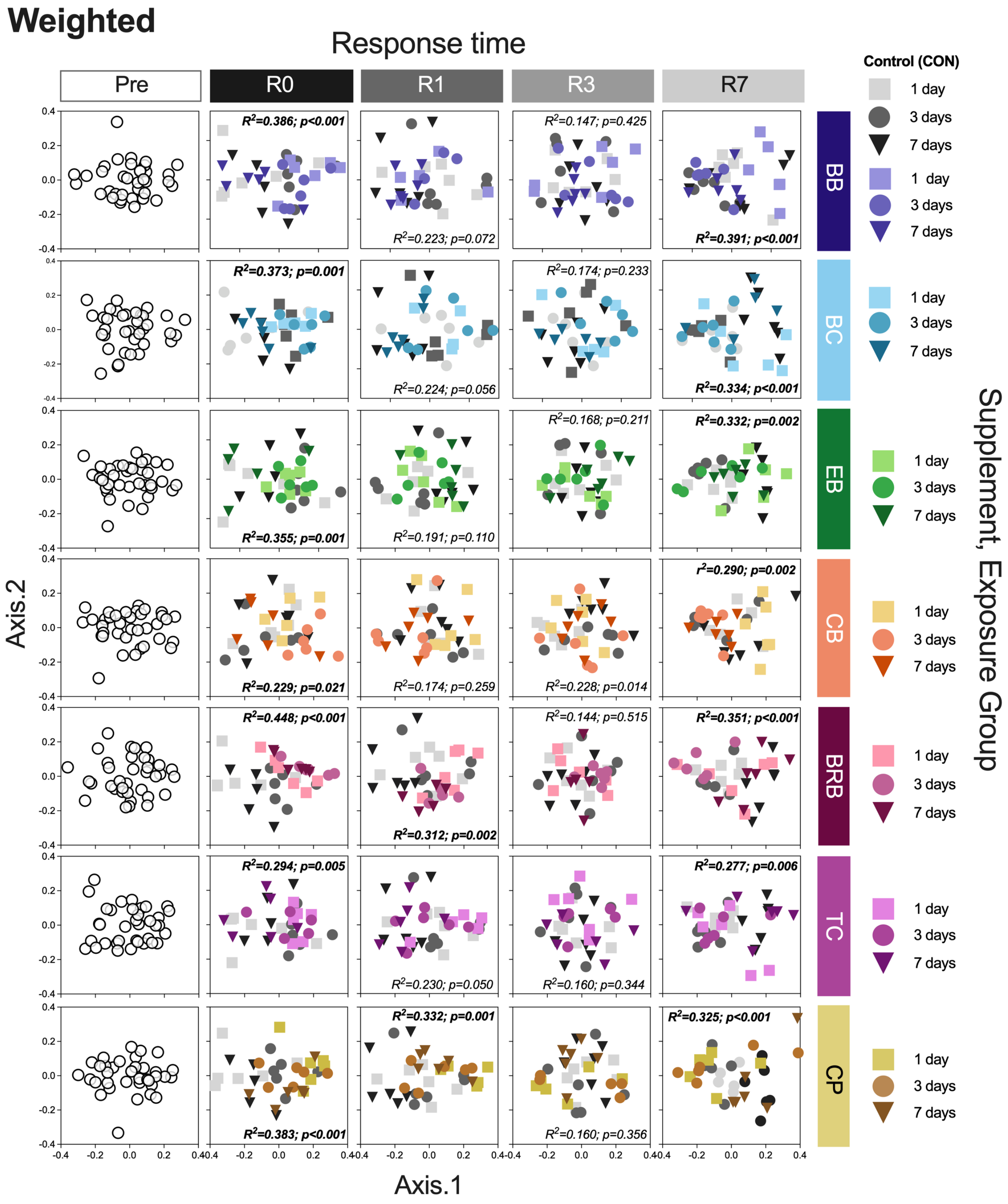

Figure S24. Weighted UniFrac beta diversity of mouse fecal microbiomes for exposure group, response time, or supplement. Principal coordinate plots show the weighted UniFrac distances for CON vs each supplement with data stratified by exposure group (■, 1 day; ●, 3 days; ▼, 7 days) or response time (in columns). PERMANOVA R^2 and *p*-values shown. FDR adjusted $p < 0.05$ was considered statistically significant, with a meaningful effect size set at $R^2 > 0.1$. PEMANOVA pairwise comparison results are provided in Table 2.25.

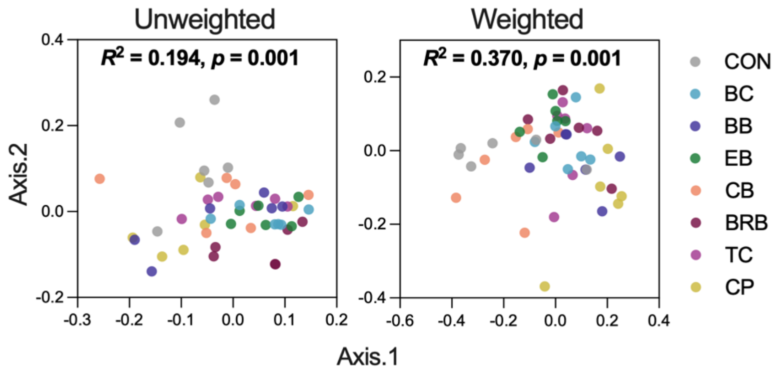

Figure S25. Beta diversity of mouse fecal microbiomes for supplement with exposure group = 1 day and response time = R0. Principal coordinate plots show the unweighted or unweighted UniFrac distances, and PERMANOVA R^2 and p-values shown. FDR adjusted $p < 0.05$ was considered statistically significant, with a meaningful effect size set at $R^2 > 0.1$. PEMANOVA pairwise comparison results are provided in Tables S28. CON, control; BB, bilberry; BC, black currant; EB, elderberry; CB, chokeberry; BRB, black raspberry; TC, tart cherry; CP, cocoa polyphenols.

Table S1. Measured anthocyanin contents for selected berry supplements.

Source	Brand name	Type	Supplier	g ACN/100 g powder
Bilberry (BB)	Std. Bilberry Powder, 25%	Solvent extract	Artemis-International	18.81
Black currant (BC)	Currant Craft, 11%	Spray-dried extract	Artemis-International	9.10
Elderberry (EB)	Elder Craft, 14%	Spray-dried extract	Artemis-International	14.8
Chokeberry (CB)	Aronia Craft, 7%	Spray-dried extract	Artemis-International	68.8
Black raspberry (BRB)	Black Raspberry Powder	Whole fruit powder	BerriHealth	1.87
Tart cherry (TC)	Cherry Craft, 9%	Membrane extract	Artemis-International	22.40

Table S2. Formulation of experimental diets.

	**CON**	**BC**	**BB**	**EB**	**CB**	**BRB**	**TC**	**CP**
Envigo Catalog No.	TD.180497	TD.220270	TD.220266	TD.220269	TD.220268	TD.220271	TD.220267	TD.220271
Energy density (kcal/g)	4.4	4.3	4.4	4.4	4.4	4.3	4.4	4.3
Supplement	0	21.98	10.63	13.51	2.91	106.95	8.93	26.5
Carbohydrates (g/kg diet)								
Corn Starch	230	230	230	230	230	200	230	219.9
Maltodextrin	70	70	70	70	70	70	70	70
Sucrose	261.2	239.8	250.7	248.8	258.4	229.4	252.4	261.2
Cellulose	30	30	30	30	30	0	30	18
Kcal (% of total)	50%	50%	50%	50%	50%	49.8%	50%	49.8%
Proteins (g/kg)								
Casein	190	189.5	190	189	190	175	190	186.6
L-cysteine	2.85	2.85	2.85	2.85	2.85	2.85	2.85	2.85
Kcal (% of total)	15.5%	15.5%	15.5%	15.5%	15.5%	15.6%	15.5%	15.5%
Fats (g/kg)								
Soybean oil	31.4	31.4	31.4	31.4	31.4	31.4	31.4	31.4
Anhydrous milkfat	36.3	36.3	36.3	36.3	36.3	36.3	36.3	36.3
Olive Oil	28	28	28	28	28	28	28	28
Lard	28	28	28	28	28	28	28	28
Beef Tallow	24.8	24.8	24.8	24.8	24.8	24.8	24.8	24.8
Corn Oil	16.5	16.5	16.5	16.5	16.5	16.5	16.5	16.5
Cholesterol	0.4	0.4	0.4	0.4	0.4	0.4	0.4	0.4
Kcal (% of total)	34.5%	34.5%	34.5%	34.5%	34.5%	34.6%	34.5%	34.7%

[1] Basal diet formulation based on the total Western diet (TWD) with 35 g/kg mineral mix nTWD (1110422), 10 g/kg vitamin mix nTWD (110423), 4 g/kg sodium chloride, 1.4 g/kg choline bitartrate, and 0.028 g/kg TBHQ antioxidant.

Table S3. *P*-values for main effects and interactions for all experimental factors for F:B ratio.

Effect	*p*-value
Supplement	**<0.0001**
Exposure group	**<0.0001**
Supplement*Exposure group	**<0.0001**
Response time	**0.0056**
Supplement*Response time	**<0.0001**
Exposure group*Response time	**<0.0001**
Supplement*Exposure group*Response time	**0.0435**

Table S4. Adjusted *p*-values for pairwise comparisons among exposure groups for F:B ratio, irrespective of response time or supplement.

Comparison	p-value
1 day vs 3 days	0.9494
1 day vs 7 days	**<0.0001**
3 days vs 7 days	**<0.0001**

Table S5. Adjusted *p*-values for pairwise comparisons among response times for F:B ratio, irrespective of exposure or supplement.

Comparison	*p*-value
Pre vs R0	**<0.0001**
Pre vs R1	**<0.0001**
Pre vs R3	**<0.0001**
Pre vs R7	**<0.0001**
R0 vs R1	**<0.0001**
R0 vs R3	0.0915
R0 vs R7	0.0694
R1 vs R3	**<0.0001**
R1 vs R7	**<0.0001**
R3 vs R7	1.0000

Table S6. Adjusted *p*-values for pairwise comparisons among supplement groups for F:B ratio, irrespective of exposure group or response time.

Comparison	*p*-value
BB vs BC	0.9580
BB vs BRB	0.9484
BB vs CB	0.4249
BB vs CON	0.9985
BB vs CP	0.1596
BB vs EB	0.7790
BB vs TC	0.9998
BC vs BRB	0.3462
BC vs CB	0.9781
BC vs CON	0.9998
BC vs CP	**0.0067**
BC vs EB	0.9998
BC vs TC	0.9984
BRB vs CB	**0.0306**
BRB vs CON	0.6457
BRB vs CP	0.8357
BRB vs EB	0.1325
BRB vs TC	0.7591
CB vs CON	0.8289
CB vs CP	**0.0001**
CB vs EB	0.9995
CB vs TC	0.7406
CON vs CP	**0.0290**
CON vs EB	0.9837
CON vs TC	1.0000
CP vs EB	**0.0011**
CP vs TC	0.0509
EB vs TC	0.9600

Table S7. Adjusted *p*-values for pairwise comparisons among exposure groups within each supplement group for F:B ratio (*Q1*).

	Supplement							
**Comparison**	**CON**	**BB**	**BC**	**EB**	**CB**	**BRB**	**TC**	**CP**
1 day vs 3 days	0.3324	0.9265	0.6117	0.8195	0.1769	0.9965	0.6682	0.5513
1 day vs 7 days	0.3442	0.2548	0.3664	0.5240	0.1971	0.2813	0.1140	**0.0053**
3 days vs 7 days	**0.0165**	0.4531	0.9026	0.2114	0.9996	0.2523	0.4949	0.0853

Table S8. Adjusted *p*-values for pairwise comparisons among response times within each supplement group for F:B ratio (*Q2)*.

	Supplement							
**Comparison**	**CON**	**BB**	**BC**	**EB**	**CB**	**BRB**	**CP**	**TC**
Pre vs R0	**0.0021**	**0.0034**	0.1952	**0.0062**	**0.0001**	0.9943	0.9120	**0.0062**
Pre vs R1	**0.0001**	**0.0001**	**0.0001**	**0.0001**	**0.0001**	**0.0001**	**0.0032**	**0.0001**
Pre vs R3	0.0696	**0.0001**	**0.0001**	**0.0008**	**0.0014**	**0.0075**	0.0617	**0.0001**
Pre vs R7	**0.0119**	**0.0044**	**0.0003**	**0.0022**	**0.0009**	**0.0086**	**0.0051**	**0.0001**
R0 vs R1	0.2036	**0.0007**	**0.0001**	**0.0002**	0.8689	**0.0001**	**0.0396**	**0.0053**
R0 vs R3	0.7341	0.8060	0.0644	0.9815	**0.0412**	**0.0242**	0.3461	0.8089
R0 vs R7	0.9764	1.0000	0.1649	0.9978	0.0703	**0.0277**	0.0589	0.7337
R1 vs R3	**0.0078**	**0.0235**	0.1823	**0.0010**	**0.0026**	0.0757	0.8536	0.1062
R1 vs R7	**0.0493**	**0.0006**	0.0749	**0.0006**	**0.0053**	0.0557	0.9997	0.1283
R3 vs R7	0.9662	0.7636	0.9939	0.9993	0.9998	1.0000	0.9228	1.0000

Table S9. Adjusted *p*-values for effect of supplement within each exposure group for F:B ratio (*Q3*).

	**Exposure Group**		
**Comparison**	**1 day**	**3 days**	**7 days**
BB vs BRB	0.9998	0.9849	1.0000
BB vs CP	0.5423	0.9965	0.9986
BB vs TC	1.0000	0.9314	1.0000
BC vs BB	1.0000	0.9853	1.0000
BC vs BRB	0.9952	0.6055	0.9999
BC vs CP	0.3752	0.9999	0.9969
BC vs TC	0.9999	0.7284	1.0000
BRB vs CP	0.8277	0.4084	1.0000
CB vs BB	0.0644	0.9961	0.9989
CB vs BC	0.1676	0.9997	0.9996
CB vs BRB	**0.0164**	1.0000	0.9836
CB vs CON	0.5170	0.9996	0.9311
CB vs CP	**<0.0001**	0.8396	0.9881
CB vs EB	0.6560	0.9172	1.0000
CB vs TC	**0.0489**	0.6685	1.0000
CON vs BB	0.9716	1.0000	1.0000
CON vs BC	0.9974	1.0000	1.0000
CON vs BRB	0.8199	1.0000	0.9992
CON vs CP	0.0728	0.9997	0.9997
CON vs TC	0.9490	1.0000	0.9860
EB vs BB	0.9326	0.9560	0.9381
EB vs BC	0.9891	0.9851	0.9900
EB vs BRB	0.7191	0.8593	1.0000
EB vs CON	1.0000	0.9999	1.0000
EB vs CP	**0.0466**	0.8928	1.0000
EB vs TC	0.8948	0.9506	0.9972
TC vs BRB	1.0000	0.7489	0.9784
TC vs CP	0.6375	1.0000	0.9984

Table S10. Adjusted *p*-values for effect of supplement within each response time for FB ratio (*Q4*).

	Response time			
**Comparison**	**R0**	**R1**	**R3**	**R7**
BB vs BC	0.9942	1.0000	0.9898	0.8962
BB vs BRB	0.0946	0.9998	0.9998	0.9995
BB vs CB	**0.0094**	1.0000	0.9994	0.9974
BB vs CON	0.9046	0.9984	0.9534	0.9986
BB vs CP	0.2447	0.1253	0.9170	0.9946
BB vs EB	0.9995	0.9987	1.0000	0.9956
BB vs TC	1.0000	1.0000	1.0000	0.9401
BC vs BRB	0.4435	0.9998	0.9055	0.9934
BC vs CB	**0.0006**	1.0000	0.8664	0.9986
BC vs CON	0.4575	0.9981	0.5240	0.9970
BC vs CP	0.7266	0.1302	0.4441	0.9993
BC vs EB	0.9015	0.9991	0.9862	0.9993
BC vs TC	0.9942	1.0000	0.9958	1.0000
BRB vs CB	**<0.0001**	0.9945	1.0000	1.0000
BRB vs CON	**0.0019**	1.0000	0.9981	1.0000
BRB vs CP	0.9999	0.3392	0.9936	1.0000
BRB vs EB	**0.0247**	0.9654	0.9999	1.0000
BRB vs TC	0.1019	1.0000	0.9994	0.9981
CB vs CON	0.3041	0.9795	0.9991	1.0000
CB vs CP	**<0.0001**	0.0551	0.9964	1.0000
CB vs EB	0.0544	1.0000	0.9983	1.0000
CB vs TC	**0.0116**	0.9989	1.0000	0.9998
CON vs CP	**0.0085**	0.3998	0.9306	1.0000
CON vs EB	0.9955	0.9140	0.8856	1.0000
CON vs TC	0.9143	1.0000	0.9997	0.9993
CP vs EB	0.0801	**0.0217**	0.9628	1.0000
CP vs TC	0.2564	0.9876	0.9309	0.9999
EB vs TC	0.9996	0.2244	1.0000	0.9999

Table S11. Adjusted *p*-values for effects of supplement for exposure group = 1 day and response time = R0 for F:B ratio (*Q5*).

Comparison	*p*-value
BB vs CP	0.9978
BC vs BB	0.9996
BC vs BRB	1.0000
BC vs CP	0.9416
BRB vs BB	1.0000
BRB vs CP	0.9699
CB vs BB	**0.0053**
CB vs BC	**0.0208**
CB vs BRB	**0.0145**
CB vs CON	1.0000
CB vs CP	**0.0008**
CB vs EB	0.0934
CB vs TC	**0.0370**
CON vs BB	**0.0102**
CON vs BC	**0.0379**
CON vs BRB	**0.0268**
CON vs CP	**0.0016**
CON vs EB	0.1538
CON vs TC	0.0653
EB vs BB	0.9510
EB vs BC	0.9985
EB vs BRB	0.9947
EB vs CP	0.6485
EB vs TC	0.9999
TC vs BB	0.9959
TC vs BC	1.0000
TC vs BRB	1.0000
TC vs CP	0.8643

Table S12. *P*-values for main effects and interactions of all experimental factors for alpha diversity.

	Alpha Diversity Test	
**Effect**	**Chao1**	**Shannon**
Supplement	**0.0232**	**0.0001**
Exposure group	0.6888	0.7515
Supplement*Exposure group	0.4976	**0.0023**
Response time	0.0849	**0.0083**
Supplement*Response time	0.1277	0.1142
Exposure group*Response time	0.6123	**0.0441**
Supplement*Exposure group*Response time	0.2984	0.3434

Table S13. Adjusted *p*-values for pairwise comparisons among exposure groups for alpha diversity, irrespective of response time or supplement.

	Alpha Diversity Test	
**Comparison**	**Chao1**	**Shannon**
1 day vs 3 days	0.8856	0.9520
1 day vs 7 days	0.9186	0.7332
3 days vs 7 days	0.6642	0.8928

Table S14. Adjusted *p*-values for pairwise comparisons among response times for alpha diversity, irrespective of exposure group or supplement.

	Alpha Diversity Test	
**Comparison**	**Chao1**	**Shannon**
Pre vs R0	0.2704	**0.0490**
Pre vs R1	0.8904	1.0000
Pre vs R3	1.0000	0.9569
Pre vs R7	0.9649	0.9492
R0 vs R1	0.8327	0.0660
R0 vs R3	0.3238	0.2539
R0 vs R7	0.0652	**0.0053**
R1 vs R3	0.9234	0.9718
R1 vs R7	0.5242	0.9351
R3 vs R7	0.9467	0.6156

Table S15. Adjusted *p*-values for pairwise comparisons among supplement groups for alpha diversity, irrespective of exposure group or response time.

	Alpha Diversity Test	
**Comparison**	**Chao1**	**Shannon**
BB vs BC	0.9664	0.9985
BB vs BRB	0.8996	0.9952
BB vs CB	0.5432	0.9981
BB vs CON	**0.0396**	0.2797
BB vs CP	0.1206	**0.0063**
BB vs EB	0.9995	0.9954
BB vs TC	0.9959	0.9977
BC vs BRB	1.0000	0.8719
BC vs CB	0.9905	0.9103
BC vs CON	0.4582	0.0692
BC vs CP	0.7281	**0.0006**
BC vs EB	0.9996	1.0000
BC vs TC	1.0000	0.9050
BRB vs CB	0.9988	1.0000
BRB vs CON	0.6182	0.7750
BRB vs CP	0.8564	0.0744
BRB vs EB	0.9948	0.8087
BRB vs TC	0.9993	1.0000
CB vs CON	0.9321	0.7081
CB vs CP	0.9932	0.0543
CB vs EB	0.8687	0.8580
CB vs TC	0.9410	1.0000
CON vs CP	0.2603	0.8768
CON vs EB	0.9999	**0.0460**
CON vs TC	0.5081	0.7238
CP vs EB	0.1661	**0.0003**
CP vs TC	0.3698	0.0589
EB vs TC	1.0000	0.8512

Table S16. Adjusted *p*-values for pairwise comparisons among exposure groups within each supplement group for alpha diversity (*Q1*).

	Supplement							
**Comparison**	**CON**	**BB**	**BC**	**EB**	**CB**	**BRB**	**TC**	**CP**
Chao1								
1 day vs 3 days	0.7304	0.7086	0.8970	0.1052	0.8035	0.1403	0.8236	0.1593
1 day vs 7 days	0.9993	0.7272	0.9979	0.3379	0.9990	0.3557	0.9025	0.3523
3 days vs 7 days	0.7556	0.9992	0.9197	0.8042	0.7861	0.8644	0.5621	0.8809
Shannon								
1 day vs 3 days	0.1837	0.2364	0.9280	0.6081	0.5594	0.0791	0.6044	**0.0198**
1 day vs 7 days	0.7966	0.0758	0.9535	0.1722	0.8535	0.0527	0.6311	**0.0392**
3 days vs 7 days	0.5059	0.8456	0.9970	0.6591	0.8825	0.9795	0.1519	0.9558

Table S17. Adjusted *p*-values for pairwise comparisons among response times within each supplement group for alpha diversity (*Q2*).

	Supplement							
**Comparison**	**CON**	**BB**	**BC**	**BRB**	**CB**	**CP**	**EB**	**TC**
Chao1								
Pre vs R0	0.7583	0.9946	0.1809	0.9328	0.6435	0.9981	0.8140	**0.0069**
Pre vs R1	0.9797	0.9902	0.3543	0.4825	0.6168	0.8752	0.9435	0.5040
Pre vs R3	0.1458	0.9840	0.5164	0.9876	0.8551	0.7842	0.9626	0.8263
Pre vs R7	0.8949	0.7751	0.9983	0.6364	0.9800	0.9568	1.0000	1.0000
R0 vs R1	0.9741	0.9147	0.9980	0.9123	1.0000	0.7212	0.9969	0.3404
R0 vs R3	0.7812	0.9999	0.9722	0.9983	0.9947	0.6038	0.9929	0.1262
R0 vs R7	0.9985	0.5345	0.0963	0.9727	0.9222	0.8545	0.8166	**0.0091**
R1 vs R3	0.4215	0.8689	0.9984	0.7825	0.9933	0.9997	1.0000	0.9841
R1 vs R7	0.9976	0.9650	0.2166	0.9993	0.9113	0.9988	0.9435	0.5604
R3 vs R7	0.6097	0.4647	0.3432	0.8952	0.9923	0.9909	0.9623	0.8673
Shannon								
Pre vs R0	0.9799	0.2190	0.7191	0.9982	0.9997	0.5488	0.0973	**0.0009**
Pre vs R1	0.9998	0.9960	0.7358	0.9931	0.8607	0.3865	1.0000	0.9478
Pre vs R3	1.0000	0.9606	0.6849	0.9887	0.9912	0.9833	0.9960	0.9522
Pre vs R7	1.0000	0.9866	0.9919	0.9712	1.0000	0.9057	0.7327	0.9999
R0 vs R1	0.9512	0.4536	1.0000	0.9999	0.7742	0.9984	0.0799	**0.0108**
R0 vs R3	0.9847	0.6184	1.0000	0.9997	0.9690	0.8693	**0.0403**	**0.0103**
R0 vs R7	0.9733	0.0810	0.4418	0.9975	0.9995	0.9655	**0.0038**	**0.0013**
R1 vs R3	0.9996	0.9984	1.0000	1.0000	0.9836	0.7305	0.9986	1.0000
R1 vs R7	0.9999	0.9143	0.4678	0.9997	0.8721	0.8836	0.7809	0.9742
R3 vs R7	1.0000	0.7714	0.4124	0.9999	0.9930	0.9976	0.9065	0.9769

Table S18. Adjusted *p*-values for effect of supplement within each exposure group for alpha diversity (*Q3*).

	**Exposure Group**					
	**1 day**		**3 days**		**7 days**	
**Comparison**	**Chao1**	**Shannon**	**Chao1**	**Shannon**	**Chao1**	**Shannon**
BB vs BC	1.0000	0.7869	0.9994	1.0000	0.9810	0.9980
BB vs BRB	0.9961	0.3750	0.5791	0.7949	0.9090	0.2192
BB vs CB	0.9901	0.9074	0.9862	0.9054	0.7640	0.8769
BB vs CON	0.7907	0.9993	0.7932	0.1790	0.3474	0.3408
BB vs CP	0.5443	0.2842	0.9609	0.8792	0.8272	0.2518
BB vs EB	0.9171	0.2887	0.8605	1.0000	0.9963	0.8670
BB vs TC	1.0000	0.9980	1.0000	1.0000	0.9333	0.2146
BC vs BRB	0.9677	0.9991	0.8876	0.8639	1.0000	0.6233
BC vs CB	0.9997	1.0000	1.0000	0.9476	0.9984	0.9969
BC vs CON	0.9428	0.9743	0.9763	0.2355	0.9135	0.7739
BC vs CP	0.7888	**0.0047**	0.9994	0.9288	0.9996	0.6769
BC vs EB	0.7839	0.9959	0.9900	1.0000	1.0000	0.9964
BC vs TC	1.0000	0.9871	1.0000	1.0000	1.0000	0.6245
BRB vs CB	0.7650	0.9864	0.9768	1.0000	1.0000	0.9592
BRB vs CON	0.3218	0.7405	1.0000	0.9713	0.9842	1.0000
BRB vs CP	0.1494	**0.0003**	0.9938	1.0000	1.0000	1.0000
BRB vs EB	0.9995	1.0000	0.9997	0.7103	0.9994	0.9595
BRB vs TC	0.9930	0.8101	0.7031	0.6975	1.0000	1.0000
CB vs CON	0.9974	0.9963	0.9984	0.9040	0.9987	0.9903
CB vs CP	0.9619	**0.0095**	1.0000	1.0000	1.0000	0.9750
CB vs EB	0.4277	0.9653	0.9997	0.8466	0.9897	1.0000
CB vs TC	0.9948	0.9988	0.9964	0.8348	0.9999	0.9620
CON vs CP	0.9999	0.0829	0.9999	0.9331	0.9946	1.0000
CON vs EB	0.1099	0.6413	1.0000	0.1262	0.8181	0.9905
CON vs TC	0.8384	1.0000	0.8822	0.1256	0.9693	1.0000
CP vs EB	**0.0407**	**0.0002**	1.0000	0.8128	0.9962	0.9752
CP vs TC	0.6083	0.0664	0.9859	0.8004	1.0000	1.0000
EB vs TC	0.8932	0.7207	0.9289	1.0000	0.9998	0.9623

Table S19. Adjusted *p*-values for effect of supplement within each response time for alpha diversity (*Q4*).

	Chao1				Shannon			
**Comparison**	**R0**	**R1**	**R3**	**R7**	**R0**	**R1**	**R3**	**R7**
BB vs BC	1.0000	0.9518	0.9143	0.6490	0.4359	1.0000	0.9998	0.9812
BB vs BRB	0.9954	1.0000	0.9849	0.4691	0.9970	1.0000	1.0000	0.5762
BB vs CB	0.9882	1.0000	1.0000	1.0000	1.0000	1.0000	1.0000	1.0000
BB vs CON	0.5599	0.3546	1.0000	0.9389	1.0000	0.7821	0.8715	0.9682
BB vs CP	0.9999	0.6399	0.9996	0.8107	0.7144	0.4789	0.8855	0.5827
BB vs EB	0.9977	1.0000	0.9781	0.9921	0.1026	1.0000	1.0000	0.9660
BB vs TC	0.3902	1.0000	0.9999	0.7898	0.1800	0.9968	0.9836	0.8238
BC vs BRB	0.9999	0.9720	1.0000	1.0000	0.8646	0.9999	1.0000	0.9859
BC vs CB	0.9342	0.9934	0.8732	0.8073	0.4863	1.0000	0.9978	0.9930
BC vs CON	0.3568	0.9675	0.7717	0.9988	0.3706	0.8984	0.6205	1.0000
BC vs CP	0.9971	0.9984	0.6647	1.0000	**0.0066**	0.6476	0.6466	0.9867
BC vs EB	1.0000	0.9917	1.0000	0.9823	0.9948	1.0000	0.9984	1.0000
BC vs TC	0.5973	0.9941	0.9925	1.0000	0.9996	0.9998	0.8747	0.9996
BRB vs CB	0.7407	1.0000	0.9713	0.6500	0.9986	0.9996	0.9995	0.6835
BRB vs CON	0.1566	0.4335	0.9254	0.9894	0.9918	0.6585	0.7140	0.9910
BRB vs CP	0.9513	0.7167	0.8582	0.9995	0.2736	0.3579	0.7369	1.0000
BRB vs EB	1.0000	1.0000	1.0000	0.9354	0.4081	1.0000	0.9997	0.9933
BRB vs TC	0.8492	1.0000	0.9998	0.9997	0.5703	0.9842	0.9259	0.9999
CB vs CON	0.9686	0.5749	1.0000	0.9842	1.0000	0.9144	0.9426	0.9868
CB vs CP	0.9997	0.8378	0.9999	0.9213	0.6652	0.6726	0.9501	0.6896
CB vs EB	0.7929	1.0000	0.9603	0.9992	0.1230	1.0000	1.0000	0.9855
CB vs TC	0.0612	1.0000	0.9994	0.9086	0.2109	0.9999	0.9964	0.8931
CON vs CP	0.7956	0.9999	1.0000	1.0000	0.8064	0.9997	1.0000	0.9916
CON vs EB	0.1957	0.5425	0.9037	1.0000	0.0813	0.7921	0.9347	1.0000
CON vs TC	**0.0028**	0.5844	0.9954	1.0000	0.1455	0.9911	0.9999	0.9999
CP vs EB	0.9678	0.8174	0.8263	0.9979	**0.0005**	0.4864	0.9430	0.9938
CP vs TC	0.1968	0.8444	0.9828	1.0000	**0.0013**	0.8946	0.9999	0.9999
EB vs TC	0.8267	1.0000	0.9995	0.9970	1.0000	0.9975	0.9954	0.9999

Table S20. Adjusted *p*-values for effects of supplement for exposure group = 1 day and response time = R0 for alpha diversity (*Q5*).

Comparison	Chao1	Shannon
BB vs BC	0.9917	0.2006
BB vs BRB	0.9156	0.1625
BB vs CB	0.9978	0.8839
BB vs CON	0.8521	0.9771
BB vs CP	0.9052	0.7980
BB vs EB	0.8874	**0.0225**
BB vs TC	0.7658	0.2573
BC vs BRB	0.9999	1.0000
BC vs CB	0.8248	0.9106
BC vs CON	0.3693	0.7485
BC vs CP	0.4473	**0.0048**
BC vs EB	0.9996	0.9785
BC vs TC	0.9942	1.0000
BRB vs CB	0.5676	0.8764
BRB vs CON	0.1730	0.6836
BRB vs CP	0.2228	**0.0035**
BRB vs EB	1.0000	0.9892
BRB vs TC	1.0000	1.0000
CB vs CON	0.9947	1.0000
CB vs CP	0.9984	0.1150
CB vs EB	0.5168	0.3804
CB vs TC	0.3635	0.9529
CON vs CP	1.0000	0.2369
CON vs EB	0.1476	0.2047
CON vs TC	0.0856	0.8217
CP vs EB	0.1920	**0.0003**
CP vs TC	0.1146	**0.0071**
EB vs TC	1.0000	0.9559

Table S21. R^2 and adjusted PERMANOVA p-values for pairwise comparisons among exposure groups for UniFrac beta diversity, irrespective of response time or supplement.

	Unweighted		Weighted	
**Comparison**	R^2	p-value	R^2	p-value
1 day vs 3 days	0.014	0.001	0.010	0.001
1 day vs 7 days	0.024	0.001	0.025	0.001
3 days vs 7 days	0.013	0.001	0.036	0.001

Table S22. R^2 and adjusted PERMANOVA p-values for pairwise comparisons among response times for UniFrac beta diversity, irrespective of exposure group or supplement.

	Unweighted		Weighted	
**Comparison**	R^2	p-value	R^2	p-value
Pre vs R0	0.0525	0.001	**0.1777**	**0.001**
Pre vs R1	0.0128	0.001	0.0147	0.003
Pre vs R3	0.0373	0.001	0.0426	0.001
Pre vs R7	0.0253	0.001	0.0659	0.001
R0 vs R1	0.0702	0.001	**0.1495**	**0.001**
R0 vs R3	0.0477	0.001	**0.1373**	**0.001**
R0 vs R7	0.0634	0.001	**0.2986**	**0.001**
R1 vs R3	0.0593	0.001	0.0390	0.001
R1 vs R7	0.0470	0.001	0.0945	0.001
R3 vs R7	0.0236	0.001	**0.1037**	**0.001**

Table S23. R^2 and adjusted PERMANOVA *p*-values for pairwise comparisons among supplements for UniFrac beta diversity, irrespective of exposure group or response time.

	Unweighted		Weighted	
**Comparison**	R^2	***p*-value**	R^2	***p*-value**
BB vs BC	0.019	0.0012	0.004	0.6520
BB vs BRB	0.031	0.0012	0.019	0.0187
BB vs CON	0.021	0.0012	0.013	0.0805
BB vs TC	0.015	0.0041	0.007	0.3398
BC vs BRB	0.026	0.0012	0.026	0.0056
CB vs BB	0.026	0.0012	0.018	0.0336
CB vs BC	0.022	0.0012	0.010	0.1857
CB vs BRB	0.046	0.0012	0.043	0.0035
CB vs CON	0.017	0.0032	0.007	0.3398
CB vs TC	0.016	0.0022	0.007	0.3433
CON vs BC	0.021	0.0012	0.010	0.1680
CON vs BRB	0.042	0.0012	0.034	0.0035
CP vs BB	0.026	0.0012	0.020	0.0178
CP vs BC	0.026	0.0012	0.032	0.0056
CP vs BRB	0.030	0.0012	0.019	0.0237
CP vs CB	0.032	0.0012	0.057	0.0035
CP vs CON	0.027	0.0012	0.037	0.0035
CP vs EB	0.025	0.0012	0.055	0.0035
CP vs TC	0.033	0.0012	0.032	0.0035
EB vs BB	0.016	0.0012	0.016	0.0336
EB vs BC	0.012	0.0260	0.008	0.2632
EB vs BRB	0.028	0.0012	0.044	0.0035
EB vs CB	0.017	0.0012	0.005	0.5310
EB vs CON	0.025	0.0012	0.011	0.1482
EB vs TC	0.019	0.0012	0.007	0.3745
TC vs BC	0.024	0.0012	0.005	0.5310
TC vs BRB	0.053	0.0012	0.032	0.0035
TC vs CON	0.021	0.0012	0.006	0.3998

Table S24. R^2 and adjusted PERMANOVA *p*-values for pairwise comparisons among exposure groups for UniFrac beta diversity within each supplement group, irrespective of response time (*Q1*).

	Unweighted (R^2, *p*-value)			Weighted (R^2, *p*-value)		
**Supp**	**1 d vs 3 d**	**1 d vs 7 d**	**3 d vs 7 d**	**1 d vs 3 d**	**1 d vs 7 d**	**3 d vs 7 d**
CON	0.066, 0.001	0.097, 0.001	0.061, 0.001	0.062, 0.009	0.049, 0.024	**0.117**, **0.003**
BB	0.038, 0.015	0.070, 0.003	0.037, 0.008	0.021, 0.296	0.040, 0.080	0.074, 0.003
BC	0.058, 0.002	0.044, 0.009	0.070, 0.002	0.032, 0.117	0.059, 0.011	0.083, 0.003
EB	0.025, 0.126	0.027, 0.126	0.024, 0.126	0.027, 0.157	0.053, 0.027	0.069, 0.012
CB	0.053, 0.002	0.050, 0.002	0.028, 0.102	0.052, 0.027	0.070, 0.006	0.009, 0.783
BRB	0.049, 0.003	0.057, 0.003	0.048, 0.007	0.042, 0.061	0.039, 0.061	0.039, 0.061
TC	0.045, 0.002	0.066, 0.002	0.048, 0.002	0.025, 0.213	**0.103**, **0.003**	0.052, 0.038
CP	0.033, 0.044	0.059, 0.003	0.047, 0.009	0.041, 0.065	0.087, 0.003	0.045, 0.044

Table S25. R^2 and adjusted PERMANOVA p-values for pairwise comparisons among response times for UniFrac beta diversity within each supplement group, irrespective of exposure group (*Q*2).

	Supplement (R^2, p-value)							
**Comparison**	**CON**	**BB**	**BC**	**EB**	**CB**	**BRB**	**TC**	**CP**
Unweighted								
Pre vs R0	0.028, 0.680	0.088, 0.002	**0.133, 0.003**	**0.140, 0.001**	0.062, 0.028	**0.193, 0.001**	**0.135, 0.002**	**0.111, 0.003**
Pre vs R1	0.043, 0.423	**0.135, 0.002**	0.067, 0.018	**0.135, 0.001**	**0.121, 0.003**	**0.130, 0.001**	0.082, 0.002	0.089, 0.003
Pre vs R3	0.054, 0.170	0.081, 0.002	0.030, 0.446	0.087, 0.001	0.071, 0.003	**0.117, 0.001**	0.082, 0.002	**0.135, 0.003**
Pre vs R7	0.063, 0.150	**0.101, 0.002**	0.068, 0.018	**0.115, 0.001**	**0.137, 0.003**	0.068, 0.007	**0.131, 0.002**	**0.137, 0.003**
R1 vs R0	0.020, 0.829	0.036, 0.331	0.078, 0.018	**0.122, 0.001**	0.034, 0.368	**0.109, 0.001**	0.049, 0.093	0.039, 0.216
R3 vs R0	0.022, 0.829	0.075, 0.004	**0.112, 0.003**	**0.118, 0.001**	0.026, 0.606	**0.211, 0.001**	0.097, 0.002	0.075, 0.015
R7 vs R0	0.040, 0.454	0.098, 0.002	**0.151, 0.003**	**0.171, 0.001**	0.059, 0.042	**0.239, 0.001**	**0.146, 0.002**	0.089, 0.004
R1 vs R3	0.035, 0.454	0.065, 0.014	0.033, 0.446	0.070, 0.007	0.042, 0.190	0.092, 0.001	0.042, 0.167	0.037, 0.303
R1 vs R7	0.031, 0.630	**0.107, 0.002**	**0.067, 0.026**	0.087, 0.003	0.059, 0.057	**0.129, 0.001**	0.095, 0.003	0.063, 0.026
R3 vs R7	0.018, 0.829	0.031, 0.381	0.032, 0.446	0.049, 0.047	0.021, 0.739	0.057, 0.011	0.046, 0.093	0.014, 0.899
Weighted								
Pre vs R0	**0.137, 0.006**	**0.221, 0.002**	**0.144, 0.002**	**0.169, 0.003**	**0.286, 0.002**	**0.260, 0.001**	**0.186, 0.003**	**0.240, 0.002**
Pre vs R1	**0.264, 0.006**	**0.362, 0.002**	**0.289, 0.002**	**0.341, 0.003**	**0.351, 0.002**	**0.390, 0.001**	**0.356, 0.003**	**0.329, 0.002**
Pre vs R3	**0.129, 0.006**	**0.201, 0.002**	**0.195, 0.002**	**0.189, 0.003**	**0.195, 0.002**	**0.221, 0.001**	**0.287, 0.003**	**0.187, 0.002**
Pre vs R7	**0.148, 0.006**	**0.166, 0.002**	**0.160, 0.002**	**0.133, 0.004**	**0.208, 0.002**	**0.142, 0.002**	**0.219, 0.003**	**0.152, 0.002**
R1 vs R0	0.070, 0.066	**0.159, 0.002**	**0.183, 0.003**	**0.161, 0.003**	0.010, 0.916	**0.277, 0.001**	**0.134, 0.010**	**0.140, 0.003**
R3 vs R0	0.015, 0.853	0.079, 0.033	**0.152, 0.002**	0.063, 0.082	0.076, 0.030	**0.217, 0.001**	0.073, 0.060	**0.120, 0.007**
R7 vs R0	0.011, 0.853	0.077, 0.025	**0.124, 0.003**	0.079, 0.043	0.088, 0.021	**0.181, 0.001**	0.046, 0.180	**0.127, 0.002**
R1 vs R3	0.086, 0.018	**0.104, 0.010**	0.041, 0.263	**0.120, 0.004**	**0.111, 0.012**	**0.138, 0.001**	0.049, 0.180	0.059, 0.106
R1 vs R7	**0.120, 0.006**	**0.161, 0.002**	0.086, 0.024	**0.135, 0.004**	**0.143, 0.002**	**0.186, 0.001**	0.088, 0.047	0.065, 0.081
R3 vs R7	0.033, 0.408	0.025, 0.491	0.028, 0.480	0.031, 0.350	0.018, 0.720	0.044, 0.170	0.028, 0.399	0.011, 0.861

Table S26. R^2 and adjusted PERMANOVA *p*-values for pairwise comparisons among supplements for UniFrac beta diversity within each exposure group, irrespective of response time (*Q3*).

	Unweighted (R^2, *p*-value)			Weighted (R^2, *p*-value)		
**Comparison**	**1 day**	**3 days**	**7 days**	**1 day**	**3 days**	**7 days**
CON vs BB	0.039, 0.020	0.035, 0.017	0.064, 0.002	0.009, 0.786	0.019, 0.366	0.033, 0.177
CON vs BC	0.049, 0.005	0.052, 0.004	0.059, 0.002	0.015, 0.501	0.060, 0.049	0.008, 0.803
CON vs BRB	0.063, 0.003	0.043, 0.007	0.056, 0.002	0.064, 0.042	0.038, 0.151	0.030, 0.177
CON vs CB	0.046, 0.014	0.051, 0.003	0.033, 0.019	0.025, 0.267	0.031, 0.175	0.023, 0.277
CON vs CP	0.032, 0.064	0.050, 0.004	0.044, 0.002	0.047, 0.073	0.034, 0.166	0.018, 0.431
CON vs EB	0.026, 0.131	0.038, 0.008	0.067, 0.002	0.023, 0.270	0.046, 0.117	0.059, 0.019
CON vs TC	0.041, 0.013	0.043, 0.007	0.057, 0.003	0.009, 0.766	0.064, 0.049	0.024, 0.277
BB vs BC	0.035, 0.022	0.045, 0.004	0.076, 0.002	0.021, 0.331	0.034, 0.166	0.043, 0.086
BB vs BRB	0.042, 0.011	0.078, 0.003	0.041, 0.006	0.026, 0.267	0.057, 0.062	0.016, 0.445
BB vs CB	0.060, 0.003	0.036, 0.023	0.069, 0.002	0.065, 0.016	0.043, 0.140	0.035, 0.177
BB vs CP	0.066, 0.003	0.050, 0.003	0.084, 0.002	0.031, 0.156	0.032, 0.175	0.073, 0.011
BB vs EB	0.035, 0.027	0.035, 0.025	0.049, 0.002	0.049, 0.048	0.015, 0.480	0.073, 0.012
BB vs TC	0.078, 0.003	0.036, 0.021	0.044, 0.003	0.099, 0.007	0.028, 0.224	0.024, 0.277
BC vs TC	0.064, 0.003	0.060, 0.003	0.051, 0.002	0.043, 0.052	0.039, 0.159	0.031, 0.177
BRB vs BC	0.050, 0.005	0.059, 0.003	0.076, 0.002	0.189, 0.007	0.025, 0.265	0.064, 0.020
BRB vs CP	0.049, 0.003	0.058, 0.003	0.098, 0.002	0.091, 0.009	0.035, 0.166	0.088, 0.011
BRB vs TC	0.048, 0.010	0.061, 0.003	0.073, 0.002	0.036, 0.125	0.038, 0.166	0.067, 0.020
CB vs BC	0.085, 0.003	0.066, 0.003	0.070, 0.002	0.048, 0.048	0.028, 0.231	0.066, 0.012
CB vs BRB	0.049, 0.003	0.057, 0.003	0.063, 0.002	**0.119, 0.007**	0.025, 0.307	0.070, 0.011
CB vs CP	0.052, 0.003	0.033, 0.029	0.069, 0.002	0.049, 0.044	0.034, 0.166	0.115, 0.011
CB vs TC	0.036, 0.036	**0.105, 0.003**	**0.107, 0.002**	0.083, 0.009	0.065, 0.049	0.018, 0.441
CP vs BC	0.045, 0.006	0.069, 0.003	0.030, 0.049	0.048, 0.062	0.020, 0.354	0.016, 0.445
CP vs TC	0.025, 0.170	0.039, 0.013	0.045, 0.005	0.020, 0.382	0.004, 0.958	0.030, 0.177
EB vs BC	0.045, 0.009	0.041, 0.007	0.041, 0.005	0.037, 0.086	0.021, 0.345	0.079, 0.011
EB vs BRB	0.037, 0.017	0.042, 0.007	0.095, 0.002	0.031, 0.162	0.032, 0.166	0.022, 0.308
EB vs CB	0.052, 0.006	0.025, 0.105	0.057, 0.003	0.016, 0.501	0.020, 0.340	0.032, 0.177
EB vs CP	0.046, 0.006	0.045, 0.004	0.047, 0.004	**0.125, 0.007**	0.056, 0.049	0.032, 0.177
EB vs TC	0.044, 0.009	0.029, 0.053	0.041, 0.006	0.050, 0.052	0.021, 0.345	0.087, 0.014

Table S27. R^2 and adjusted PERMANOVA *p*-values for pairwise comparisons among supplements for UniFrac beta diversity within each response time, irrespective of exposure group (*Q4*).

	Unweighted (R^2, *p*-value)				Weighted (R^2, *p*-value)			
**Comparison**	**R0**	**R1**	**R3**	**R7**	**R0**	**R1**	**R3**	**R7**
BB vs BC	**0.136, 0.001**	0.019, 0.833	0.035, 0.442	0.020, 0.957	0.026, 0.440	0.007, 0.983	0.030, 0.771	0.014, 0.986
BB vs BRB	**0.161, 0.001**	0.060, 0.056	0.062, 0.062	0.028, 0.957	**0.119, 0.010**	0.079, 0.067	0.039, 0.771	0.013, 0.986
BB vs CB	0.063, 0.005	0.053, 0.084	0.036, 0.432	0.034, 0.957	**0.151, 0.005**	0.028, 0.730	0.006, 0.964	0.014, 0.986
BB vs CON	0.095, 0.001	0.087, 0.012	0.030, 0.458	0.016, 0.957	0.084, 0.044	0.031, 0.637	0.020, 0.796	0.012, 0.986
BB vs CP	0.072, 0.004	0.062, 0.034	0.040, 0.322	0.027, 0.957	0.088, 0.025	**0.140, 0.020**	0.046, 0.771	0.010, 0.986
BB vs EB	**0.108, 0.001**	0.036, 0.284	0.034, 0.442	0.016, 0.957	0.065, 0.083	0.022, 0.854	0.017, 0.804	0.020, 0.986
BB vs TC	**0.115, 0.001**	0.055, 0.056	0.028, 0.562	0.023, 0.957	0.061, 0.113	0.010, 0.960	0.045, 0.771	0.018, 0.986
BC vs BRB	**0.125, 0.001**	0.043, 0.267	0.035, 0.456	0.017, 0.957	**0.110, 0.007**	**0.106, 0.025**	0.033, 0.771	0.020, 0.986
BC vs CB	**0.105, 0.001**	0.029, 0.595	0.033, 0.458	0.032, 0.957	**0.190, 0.004**	0.019, 0.884	0.022, 0.796	0.010, 0.986
BC vs CON	**0.177, 0.001**	0.054, 0.080	0.025, 0.649	0.014, 0.957	**0.110, 0.015**	0.029, 0.730	0.024, 0.796	0.008, 0.986
BC vs CP	**0.122, 0.001**	0.047, 0.123	0.046, 0.250	0.027, 0.957	**0.108, 0.011**	**0.146, 0.008**	0.069, 0.771	0.018, 0.986
BC vs EB	0.083, 0.001	0.024, 0.713	0.016, 0.899	0.018, 0.957	0.065, 0.080	0.014, 0.914	0.018, 0.804	0.013, 0.986
BC vs TC	**0.103, 0.001**	0.026, 0.686	0.033, 0.458	0.024, 0.957	0.056, 0.121	0.006, 0.983	0.023, 0.796	0.017, 0.986
BRB vs CB	**0.184, 0.001**	0.090, 0.009	0.067, 0.062	0.040, 0.957	**0.334, 0.004**	**0.135, 0.008**	0.026, 0.796	0.023, 0.986
BRB vs CON	**0.270, 0.001**	**0.122, 0.009**	0.062, 0.062	0.014, 0.957	**0.238, 0.004**	**0.119, 0.007**	0.021, 0.796	0.015, 0.986
BRB vs CP	**0.156, 0.001**	0.088, 0.009	0.056, 0.120	0.017, 0.957	**0.115, 0.005**	**0.143, 0.007**	0.025, 0.796	0.015, 0.986
BRB vs EB	**0.148, 0.001**	0.071, 0.033	0.043, 0.250	0.041, 0.957	**0.229, 0.004**	**0.132, 0.007**	0.042, 0.771	0.009, 0.986
BRB vs TC	**0.199, 0.001**	**0.133, 0.009**	0.075, 0.062	0.031, 0.957	**0.200, 0.004**	**0.101, 0.028**	0.038, 0.771	0.024, 0.986
CB vs CON	0.033, 0.359	0.059, 0.049	0.047, 0.202	0.028, 0.957	0.047, 0.180	0.014, 0.909	0.015, 0.873	0.008, 0.986
CB vs CP	0.056, 0.021	0.068, 0.016	0.067, 0.062	0.044, 0.957	**0.295, 0.004**	**0.199, 0.008**	0.044, 0.771	0.030, 0.986
CB vs EB	0.099, 0.001	0.057, 0.047	0.025, 0.621	0.032, 0.957	0.099, 0.012	0.019, 0.868	0.012, 0.888	0.004, 0.986
CB vs TC	0.088, 0.001	0.058, 0.046	0.052, 0.123	0.020, 0.957	**0.100, 0.010**	0.016, 0.884	0.031, 0.771	0.017, 0.986
CON vs CP	**0.101, 0.002**	0.065, 0.033	0.034, 0.456	0.025, 0.957	**0.184, 0.005**	**0.152, 0.011**	0.029, 0.771	0.019, 0.986
CON vs EB	**0.163, 0.001**	**0.107, 0.009**	0.029, 0.468	0.033, 0.957	0.062, 0.102	0.037, 0.452	0.021, 0.796	0.011, 0.986
CON vs TC	**0.154, 0.001**	0.063, 0.034	0.047, 0.250	0.029, 0.957	0.040, 0.236	0.019, 0.868	0.029, 0.771	0.039, 0.986
CP vs EB	**0.104, 0.001**	0.080, 0.009	0.045, 0.250	0.029, 0.957	**0.202, 0.004**	**0.212, 0.007**	0.062, 0.771	0.027, 0.986
CP vs TC	0.097, 0.001	0.074, 0.016	0.063, 0.093	0.020, 0.957	**0.147, 0.004**	**0.141, 0.020**	0.052, 0.771	0.012, 0.986
EB vs CON	0.085, 0.001	0.056, 0.049	0.038, 0.383	0.017, 0.957	0.038, 0.271	0.02, 0.868	0.038, 0.771	0.015, 0.986

Table S28. R^2 and adjusted PERMANOVA *p*-values for pairwise comparisons among supplement for UniFrac beta diversity with exposure group = 1 day and response time = R0 (*Q5*).

	Unweighted		Weighted	
**Comparison**	R^2	***p*-value**	R^2	***p*-value**
BB vs BC	0.190	0.0758	0.084	0.5105
BRB vs BB	0.180	0.0758	0.122	0.2853
BRB vs BC	**0.198**	**0.0280**	0.059	0.7010
BRB vs CB	**0.212**	**0.0331**	**0.299**	**0.0467**
BRB vs CP	**0.239**	**0.0366**	0.243	0.0665
BRB vs EB	0.130	0.1893	0.144	0.2122
BRB vs TC	**0.234**	**0.0331**	0.111	0.3518
CB vs BB	0.103	0.3184	**0.287**	**0.0467**
CB vs BC	**0.209**	**0.0327**	**0.280**	**0.0467**
CON vs BB	**0.194**	**0.0327**	**0.342**	**0.0467**
CON vs BC	**0.296**	**0.0280**	**0.318**	**0.0467**
CON vs BRB	**0.375**	**0.0280**	**0.309**	**0.0480**
CON vs CB	0.150	0.0758	0.045	0.8860
CON vs CP	**0.220**	**0.0331**	**0.448**	**0.0467**
CON vs EB	**0.257**	**0.0280**	0.225	0.1087
CON vs TC	**0.192**	**0.0331**	0.281	0.0653
CP vs BB	0.110	0.2585	0.122	0.3169
CP vs BC	**0.250**	**0.0331**	**0.262**	**0.0467**
CP vs CB	0.116	0.2352	**0.406**	**0.0467**
CP vs TC	0.177	0.0758	**0.244**	**0.0467**
EB vs BB	0.144	0.1358	**0.235**	**0.0467**
EB vs BC	0.083	0.5000	0.145	0.2128
EB vs CB	0.158	0.1076	**0.227**	**0.0480**
EB vs CP	**0.214**	**0.0366**	**0.409**	**0.0467**
EB vs TC	0.117	0.2310	0.148	0.1820
TC vs BB	0.135	0.2113	0.108	0.3675
TC vs BC	0.159	0.1076	0.093	0.4469
TC vs CB	0.119	0.2310	**0.242**	**0.0467**
